# A systematic meta-review of systematic reviews on attention deficit hyperactivity disorder

**DOI:** 10.1192/j.eurpsy.2023.2451

**Published:** 2023-11-17

**Authors:** Ashmita Chaulagain, Ingvild Lyhmann, Anne Halmøy, Tarjei Widding-Havneraas, Olav Nyttingnes, Ingvar Bjelland, Arnstein Mykletun

**Affiliations:** 1 Centre for Research and Education in Forensic Psychiatry, Haukeland University Hospital, Bergen, Norway; 2 Department of Clinical Medicine, University of Bergen, Bergen, Norway; 3 Division of Psychiatry, Haukeland University Hospital, Bergen, Norway; 4 Division for Health Services, Norwegian Institute of Public Health, Oslo, Norway; 5 Department of Community Medicine, UiT – The Arctic University of Norway, Tromsø, Norway; 6 Centre for Work and Mental Health, Nordland Hospital, Bodø, Norway

**Keywords:** Child and adolescent psychiatry, ADHD, Systematic reviews, Epidemiology, Public Health

## Abstract

**Background:**

There are now hundreds of systematic reviews on attention deficit hyperactivity disorder (ADHD) of variable quality. To help navigate this literature, we have reviewed systematic reviews on any topic on ADHD.

**Methods:**

We searched MEDLINE, PubMed, PsycINFO, Cochrane Library, and Web of Science and performed quality assessment according to the Joanna Briggs Institute Manual for Evidence Synthesis. A total of 231 systematic reviews and meta-analyses met the eligibility criteria.

**Results:**

The prevalence of ADHD was 7.2% for children and adolescents and 2.5% for adults, though with major uncertainty due to methodological variation in the existing literature. There is evidence for both biological and social risk factors for ADHD, but this evidence is mostly correlational rather than causal due to confounding and reverse causality. There is strong evidence for the efficacy of pharmacological treatment on symptom reduction in the short-term, particularly for stimulants. However, there is limited evidence for the efficacy of pharmacotherapy in mitigating adverse life trajectories such as educational attainment, employment, substance abuse, injuries, suicides, crime, and comorbid mental and somatic conditions. Pharmacotherapy is linked with side effects like disturbed sleep, reduced appetite, and increased blood pressure, but less is known about potential adverse effects after long-term use. Evidence of the efficacy of nonpharmacological treatments is mixed.

**Conclusions:**

Despite hundreds of systematic reviews on ADHD, key questions are still unanswered. Evidence gaps remain as to a more accurate prevalence of ADHD, whether documented risk factors are causal, the efficacy of nonpharmacological treatments on any outcomes, and pharmacotherapy in mitigating the adverse outcomes associated with ADHD.

## Introduction

There are hundreds of systematic reviews on attention deficit hyperactivity disorder (ADHD) of variable quality and with partly or fully overlapping scope. This literature is increasingly difficult to navigate for clinicians, researchers, and policymakers. We aim to make this large literature on ADHD more available by systematically reviewing the published systematic reviews on ADHD and highlighting recent reviews of high quality where there are overlaps.

## Methods

We performed a meta-review [1] to systematically appraise systematic reviews and meta-analyses published on ADHD-related topics by adopting Preferred Reporting Items for Systematic Reviews and Meta-analyses (PRISMA) guidelines [2] (Supplementary Table S1) and the Joanna Briggs Institute (JBI) methodology for umbrella review [3]. The study protocol was pre-registered with the International Prospective Register of Systematic Reviews (PROSPERO; CRD42020165638).

### Search strategy and selection criteria Table 1

We searched MEDLINE, PubMed, PsycINFO, Cochrane Library, and Web of Science for studies, using specific keywords (ADHD, systematic review, meta-analysis, see Supplementary 1 for a description of the search strategy) with no language restrictions. The search included all years and final search was completed in December 2021. Reference lists of included publications were also searched. All references from the literature search were imported to Endnote X7.2 [4] and then to Covidence [5]. Two reviewers (A.C. with I.L. or O.N.) independently performed title and abstract screening of all articles identified through database search and full-text screening of more than 95% of articles. Any discrepancies in assessments were resolved in consensus or by consulting the third reviewer or the last author (A.M.). To avoid overlap, when systematic reviews studied identical topics and included more than 50% of the same primary articles, we included only the latest reviews with more studies. The included latest systematic reviews and meta-analysis were of similar or high quality compared to the older reviews of same topics (Supplementary Table S2).

**Table 1.** Selection criteria

Inclusion criteria	Exclusion criteria
1. Systematic reviews with or without meta-analyses on ADHD published in peer-reviewed journals 2. Search performed in more than one database including at least PubMed or Medline 3. Involvement of two or more reviewers at any stage of the systematic review	1. Letter/Erratum/Protocols 2. No clear description of article selection process 3. Quality assessment of included studies not performed 4. Same article published in different journal 5. No full text available (after rejection from authors)

### Quality appraisal and data extraction

The JBI guideline for quality appraisal and form for data extraction of systematic review and meta-analysis [6] was amended and piloted for the purpose of this meta-review (Supplementary 1). The quality appraisal checklist consists of nine items. Reviews that scored less than six on low risk of bias were categorized as low quality and excluded. Reviews scoring six to seven, and eight to nine were categorized as moderate and high quality reviews, respectively, and were included. Two reviewers (A.C., I.L.) independently assessed the quality of 35% of the systematic reviews to ensure consistency in the quality assessment rating. There was good agreement in quality assessment, and consequently, the remaining 65% of included studies were scored for quality by one author only. A similar process was followed for data extraction, where three reviewers were involved (A.C. with I.L. or O.N.). For each eligible article, pre-defined information was extracted, including topic studied, objective, timeframe of database search, main findings with key estimates, implications for clinical practice and future research, and conclusions. Further details are in Supplementary 1.

### Data presentation

We present the objective, main findings, and conclusions of each included systematic reviews and meta-analyses in tables dividing the literature into nine topics of ADHD. In the text, we describe the literature in terms of a narrative synthesis, where for some reviews we have also presented effect estimates with 95% confidence intervals (CI) for some key findings. For overlapping reviews, we highlight results based on recency and quality. As the result section is dense, we have also included summary table that include major findings, limitations, and recommendation for future systematic reviews and meta-analyses for each topic.

## Results

**Figure 1.**
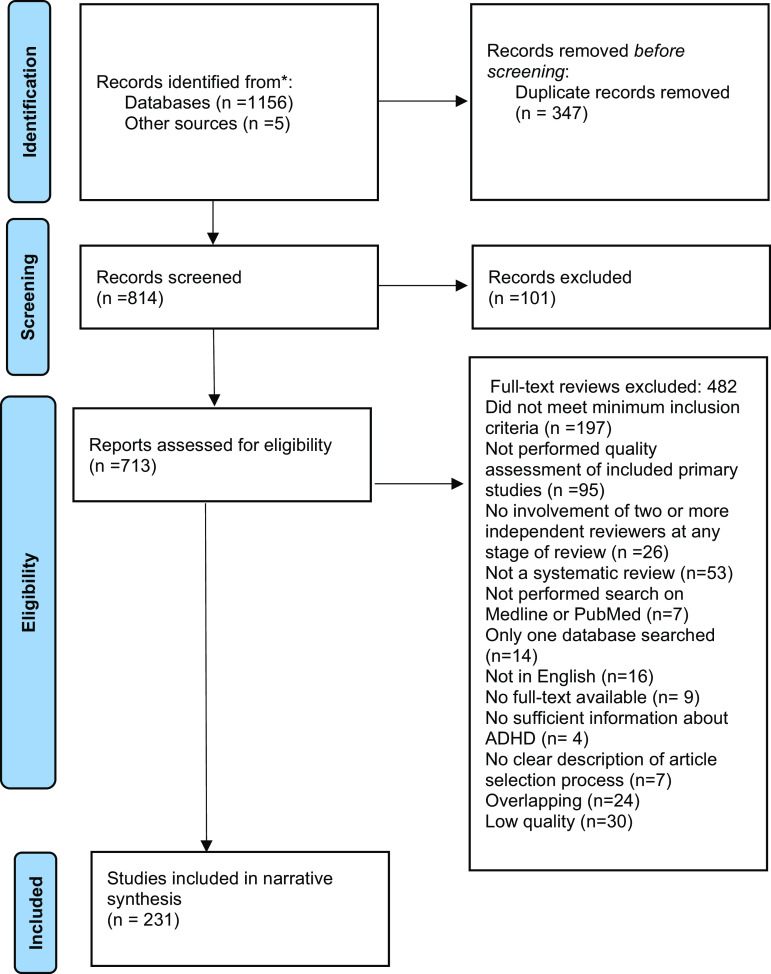
PRISMA flow diagram.

A total of 1,161 systematic reviews and meta-analyses were identified, where 231 were eligible for inclusion (Figure 1). The reasons for exclusions of each article selected for full-text review are presented in Supplementary Table S3.

### Characteristics and quality of included systematic reviews and meta-analyses

**Figure 2.**
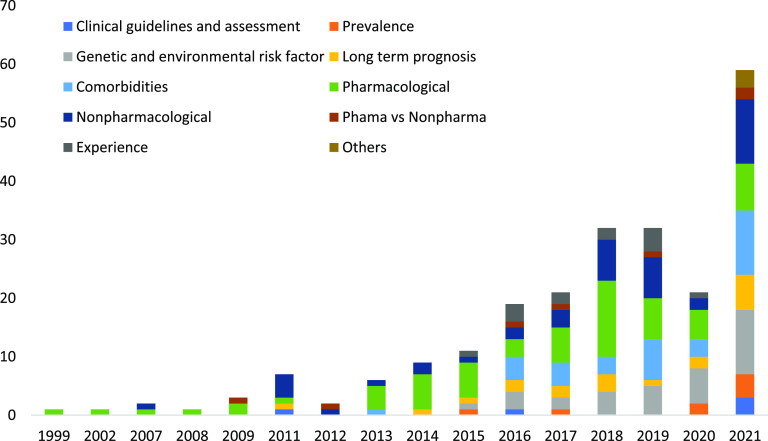
Number of systematic reviews and meta-analyses published per year on different topics.

There has been an increase in published reviews annually with a very high number of reviews published in 2021. The most common topic for reviews was pharmacological interventions (28%) (Figure 2). Most of the studies included in reviews were conducted in Europe and North America (34 and 33%, respectively). Of the total included reviews, 59% were of high quality (Tables 3–11).

### Overview of findings

The included reviews were categorized into nine different topics and the major findings for each topic are presented in Table 2.

**Table 2.** Summary table of major findings

Topics	Major findings	Limitations	Further reviews needed
Clinical guidelines and assessment	Good agreement across guidelines for stimulants as first-line medication Psychosocial interventions was recommended despite lower degree of evidence	Although guidelines recommend rating scales as part of the assessment, they vary in their emphasis and recommendation of which to use	Future reviews of rating scales should include head-to-head comparisons of different rating scales, sex-specific symptom profiles, and functional aspects of ADHD beyond core symptoms
Prevalence of ADHD	The pooled prevalence of ADHD was 7.2% in children and adolescents ADHD is twice as prevalent in boys The prevalence of ADHD is 2.5% in adults	Estimates vary between studies, regions, and according to study design and methods	Meta-analyses addressing the effect of bias (for example effect of study design, geographical location, and assessment tools) on prevalence are needed
Genetic and environmental risk factors associated with ADHD	ADHD was associated with several biological and social factors including for example genes/polygenic risk scores, maternal health in pregnancy, nutrition, repeated general anesthesia in early childhood, age at school start	Despite a huge literature on risk factors, research designs are mostly correlational rather than allowing for causal inference	Reviews of risk factors of ADHD allowing for causal inference are called for
Long-term prognosis and life trajectories in ADHD	ADHD was associated with school performance and school dropout, work participation, welfare dependency, smoking, drug and alcohol addiction, injuries, suicidal spectrum behavior, crime, and comorbidities, in directions as expected	The literature describes a rather bleak prognosis in ADHD, but this may be exaggerated due to the lack of adjustment for potential and residual confounding factors. The described outcomes of ADHD may thus not be causally linked to ADHD	Reviews of studies of prognosis in ADHD properly accounting for confounding are needed. The degree of confounding in this literature may also be subject of a meta-analysis, comparing adjusted and un-adjusted studies
ADHD and comorbidities	ADHD was associated with a high degree of comorbid somatic conditions (e.g., obesity, asthma, headache/migraine, sleep problems) and psychiatric disorders (e.g., other neurodevelopmental-, affective-, anxiety-, and eating disorders)	Studies are mostly correlational and lack data on temporality. The reviews do not sufficiently distinguish comorbidities from side-effects of medication. The causal mechanisms in the comorbidities are not addressed	Reviews addressing these limitations are needed
Pharmacological treatment	Stimulants were recommended as first-line medication both for children (methylphenidate preferred) and adults (amphetamine preferred) Nonstimulants were also found effective in treating ADHD in children (atomoxetine, guanfacine) and adults (atomoxetine, bupropion), though less effective than stimulants Side-effects were common and include reduced appetite, sleep problems, headache, increased heart rate and increased blood pressure	Most trials have short follow-up time (weeks and months rather than years) and focus mainly on core symptoms of ADHD as outcome There is no review evidence of pharmacological treatment effect on life-trajectory in ADHD over more than few months follow-up	Reviews of pharmacological treatment effects in ADHD on long-term prognosis with real-life outcomes including educational attainment, welfare dependency and employment, criminality, injuries, and mortality are urgently needed
Nonpharmacological treatment	Behavioral interventions, parental training, dietary interventions (like omega-3), and mindfulness were found to have small but positive effects in children However, the evidence for nonpharmacological treatment effects was more mixed than for pharmacological treatment Further, there are also fewer original studies and fewer reviews In adults, cognitive behavioral therapy and meditation-based therapies have shown positive effects	Most of the reviews address core ADHD symptoms and there were only few reviews that focuses on real-life outcomes and functional outcomes including for example social skills, peer relationships, school performance	Reviews of efficacy studies with outcomes beyond core ADHD symptoms are needed Reviews addressing effects of biases in this literature are needed. Biases include publication biases in favor of positive findings, the effect of nonblinded assessments should be subject for meta-analyses
Pharmacological versus nonpharmacological treatment in ADHD	Stimulants were superior to nonpharmacological treatment in reducing core symptoms among children and adolescents with ADHD While medications improved ADHD symptoms, psychosocial treatments were beneficial for academic and organizational skills in adolescents Pharmacological treatment was found to be cost-effective compared to nonpharmacological treatment or no treatment	Reviews compared medication with groups of different nonpharmacological intervention rather than comparing medication with one particular nonpharmacological intervention There is no review evidence of long-term effect of pharmacological versus nonpharmacological treatment on ADHD	Reviews comparing the long-term effects of pharmacological versus nonpharmacological treatment on ADHD are needed
Patients’ and caregivers’ experience of ADHD beyond symptoms	There was good evidence for a negative impact of ADHD on quality of life (QoL) both physically, emotionally and socially across the lifespan ADHD in children also increased parental stress. ADHD medications were chosen as a last resort and both patients and caregivers were concerned about its long-term side effects and financial costs	Although some reviews include positive aspect of having ADHD, no reviews have addressed how treatment or other interventions may influence QoL over times	Reviews assessing the impact of pharmacological or nonpharmacological interventions on QoL of patients over time are needed Reviews of qualitative studies including in-depth experiences of patients and caregivers in long-term are needed

### Narrative synthesis

#### Clinical guidelines and assessment (number of studies, n = 5, Table 3)

In a recent systematic review of five clinical practice guidelines, all guidelines rated stimulants as the first-line pharmacological intervention and recommended the inclusion of psychosocial intervention in the treatment [7].

A meta-analysis of sex differences in ADHD symptoms showed that boys with ADHD are more hyperactive than girls and have more difficulties in terms of motor response inhibition and cognitive flexibility [8].

For screening for ADHD in children, the Child Behavior Checklist-Attention Problem and the Conner’s Rating Scale–Revised had moderate sensitivity and specificity [9]. Conner’s Rating Scale and Strengths and Weaknesses of ADHD – Symptoms and Normal-Behaviors were found as valid and time-efficient measures to assess ADHD symptoms in the classroom [10].

In adults, the Conners Adult ADHD Rating Scale and the Wender Utah Rating Scale short version showed the best screening properties [11].

**Table 3.** Clinical guidelines and assessment

References	Objective	Keywords	Timeframe of database search	*k*	Sample size^a^	Analytical design	Main findings (Effect estimates, 95% CI)^b^	Conclusion and comments from the authors	Quality assessment
Razzak et al. [7]	Perform comprehensive review of clinical practice guidelines mainly emphasizing on diagnosis, evaluation and management recommendations for ADHD	Practice guidelines, Diagnosis	Not given	5	NA	Narrative synthesis	– The highest total score was achieved by the National Institute for Health and Care Excellence guidelines (91.4%) followed by the CPGs from the Scottish Intercollegiate Guidelines Network – Good agreement across guidelines about the conceptualization of ADHD, and for stimulants as the preferred first pharmacological choice of treatment – Emphasis on rating scales, psychoeducational assessment and laboratory tests varied across guidelines	Improvements in the applicability of guidelines are needed to enhance its clinical use and relevance	Moderate (7/9)
Loyer Carbonneau et al. [8]	Identify sex differences among children and adolescents with ADHD on the primary symptoms of ADHD and on executive and attentional functioning	Sex difference, Symptoms, Cognitive deficits	1997–October 2017	54	Unclear	Random-effect meta-analysis	– Included studies were of moderate quality – Boys expressed more hyperactivity symptoms than girls did (*g* = −0.15, −0.33 to 0.03) and have more difficulties in terms of motor response inhibition and cognitive flexibility – Significant heterogeneity (*I* ^2^ > 80%) were observed across analyses	Future research should refine the profile of girls with ADHD and develop diagnostic criteria adapted to each sex	Moderate (7/9)
Chang et al. [9]	Examine the diagnostic accuracy of ADHD rating scales CBCL-AP and CRS-R in children and adolescents	Rating scales, Children and adolescents	Not given	25	Ranged from 18 to 763	Random-effect meta-analysis	– Of the total included studies,11 were of high quality, the rest of poor quality – CBCL-AP yielded moderate sensitivity and specificity of 0.77 (0.69–0.84) and 0.73 (0.64–0.81) respectively – Moderate sensitivities of 0.75 (0.64–0.84), 0.72 (0.63–0.79), and 0.83 (0.59–0.95) and moderate specificities of 0.75 (0.64–0.84), 0.84 (0.69–0.93), and 0.84 (0.68–0.93) were found for CPRS-R, CTRS-R and Conners ASQ, respectively – Moderate heterogeneity (*I* ^2^ > 50%) was observed across analysis	Future meta-analyses comparing the diagnostic performance of two different tools should be conducted on the basis of studies that have directly compared the targeted tools by applying both tools to each participant or by randomizing each participant to undergo assessment by using one of the tool	High (9/9)
Staff et al. [10]	Determine the validity of teacher rating scales for assessing ADHD symptoms in the classroom	CTRS-R, SWAN, Clinical interview	1980–January 2020	4: Clinical interviews scores, 18: Structured observation scores	Clinical interviews scores: 1,744 children, Structured observation scores: 2,203 children	Random-effect meta-analysis	– Majority of studies were of high quality – Results showed convergent validity for rating scale scores, with the strongest correlations (*r* = 0.6, 0.5–0.7) for validation against interviews, and for hyperactive–impulsive behavior – Divergent validity was confirmed for teacher ratings validated against interviews, whereas validated against observations this was confirmed for inattention only – Significant heterogeneity (*I* ^2^ > 80%) were observed across analyses except for the meta-analytic correlations between rating scales and interview measures assessing inattention	Further studies with psychometric properties are needed to confirm validity of teacher rating scales	Moderate (7/9)
Taylor et al. [11]	Describe and evaluate the properties of different ADHD diagnostic rating scales in adults	Rating scales, Adults	Database inception to June 2010	35	Not given	Narrative synthesis	– Majority of studies were of poor quality – CAARS and WURS, and the WURS, short version had the best psychometric properties among 14 included scales	Additional good quality research on CAARS and WURS including larger sample size are needed	High (9/9)

Abbreviations: ASQ, Abbreviated Symptom Questionnaire; CAARS, The Conners Adult ADHD Rating scale; CBCL-AP, Child Behavior Checklist-Attention Problem; CPRS-R, Conners Parent Rating Scale – Revised; CRS-R, Conners Rating Scale – Revised; CTRS-R, Conners Teacher Rating Scale-Revised; *k*, Total number of primary studies included; NA, not applicable; SWAN, strengths and weaknesses of ADHD – Symptoms and Normal-Behaviors; WURS, Wender Utah Rating Scale.

a Total participants included in the systematic review and meta-analysis unless otherwise indicated.

b For findings from meta-analysis, if given effect estimates with 95% CI are presented unless otherwise indicated.

### Prevalence of ADHD (n = 8, Table 4)

#### Prevalence of ADHD in children and adolescents

The prevalence of ADHD among children and adolescents was assessed in four different meta-analyses [12–15] and one systematic review [16]. Internationally, the pooled prevalence of ADHD in children and adolescents was estimated to be 7.2% (95% CI: 6.7–7.8) in a meta-analysis of 175 studies including more than 1 million participants [12]. Most of the included studies were conducted within school populations (74%), and few used a whole-population approach (10%). In a multi-variable analysis, the prevalence estimate was 2 percentage points lower in studies conducted in Europe compared to North America after adjusting for the edition of diagnostic manual and measurement tools which included clinical interviews, symptom-only criteria, and reports of ADHD diagnosis [12]. Further, a meta-analysis of prevalence studies from Africa reported a pooled prevalence of 7.47% (6.0–9.26). As expected, gender differences were found with a male: female ratio of 2.0:1.0 [13]. The prevalence in China was 6.26% (5.36–7.22) [14], while it was 13.87% (9.59–19.64) among black individuals in the USA [15]. All included reviews reported that significant heterogeneity in the prevalence attributed to the source of study population, geographical location, and source of data was found across included studies.

A systematic review found substantial evidence of overdiagnosis of ADHD. The authors reported that ADHD diagnoses have consistently increased between 1989 and 2017, and that the majority of new cases were on the milder end of the ADHD spectrum [17].

#### Prevalence of ADHD in adults

The worldwide pooled prevalence among adults was 4.61% for persistent adult ADHD and 8.83% for symptomatic adult ADHD [18]. By adjusting for the “global demographic structure,” the prevalence of persistent adult ADHD was 2.58% (95% CI:1.51–4.45) and symptomatic adult ADHD 6.76% (4.31–10.61), translating to 139.84 and 366.33 million affected adults in 2020 globally. The meta-analysis found that the prevalence of ADHD decreased with age [18]. The prevalence among adults aged ≥50 was 2.2% based on validated scales applied in the general population, and 0.2% when based on clinical diagnosis [19].

**Table 4.** Prevalence of ADHD

References	Objective	Keywords	Timeframe of database search	*k*	Sample size^a^	Analytical design	Main findings (Effect estimates, 95% CI)^b^	Conclusion and comments from the authors	Quality assessment
**Children and adolescents**									
Thomas et al. [12]	Estimate worldwide prevalence of ADHD using DSM-criteria	Prevalence, DSM editions	1977–2013	175	1,023,071	Random-effect meta-analysis	– Majority of studies (75%) were of moderate or high quality – Pooled prevalence of ADHD was 7.2% (6.7–7.8) – In multivariate analyses, after adjusting for measurement and region, prevalence estimates for ADHD, was 2% point lower when DSM-III revised was applied as compared to DSM-IV – Significant heterogeneity (*I* ^2^ > 96%) were observed across analysis	Underscore that the estimated prevalence can be considered a “benchmark” – that is, deviating prevalence rates indicates over-/under diagnosis	High (8/9)
Ayano et al. [13]	Assess the prevalence of ADHD in Africa	Prevalence, Africa	Database inception – (not mentioned last date of search)	12	11,465	Random-effect meta-analysis	– Majority of studies were of high quality – Pooled prevalence of ADHD was 7.47% (6.0–9.26), with a greater prevalence among boys than girls – The prevalence rate was highest for predominantly inattentive subtype (ADHD-I) in both boys (4.05%, 3.11–5.27) and girls (2.21%, 1.61–3.03) – Significant heterogeneity (*I* ^2^ > 90%) were observed across analyses	Reasons for gender differences in ADHD needs to be explored	High (8/9)
Wang et al. [14]	Identify the prevalence of ADHD in China	Prevalence, China	Database inception – March 2016	67	275,502	Random-effect meta-analysis	– Majority of studies were of moderate quality – The prevalence rate of ADHD was 6.26% (5.36–7.22), with ADHD-I being the most common subtype – Prevalence rate varied between studies due to “geographical location” and “information sources” differences – Significant heterogeneity (*I* ^2^ > 95%) were observed across analyses	Estimated prevalence provides a “benchmark” to evaluate the disease burden of ADHD in China. However, a nation-wide research is needed to identify more “accurate estimate”	High (8/9)
Cénat et al. [15]	Estimate prevalence of ADHD among US Black individuals	Prevalence, US Black individuals	Database inception – October 2019	21	154,818	Random-effect meta-analysis	– Included studies were of moderate to high quality – Overall prevalence of ADHD was 14.5% (10.6–19.6), for individuals less than 18 years 13.9% (9.6–19.6) – Significant heterogeneity (*I* ^2^ = 99.7% were observed across analyses	Assessment and monitoring of ADHD among black individuals needs to be increased and research on ADHD prevalence across different ethnic groups in other western countries are needed	High (8/9)
Shooshtari et al. [16]	Provide an up to date prevalence of ADHD in Iran	Prevalence, Iran	January 1990–December 2018	36	33,621 children, adolescents, and adults	Narrative synthesis	– Prevalence estimates varied substantially across the studies and provided a range of heterogeneous data – Total prevalence of ADHD ranged between 11.0–25.8% among pre-school children; between 3.1–17.3% among school children, and between 3.9–25.1% among adults	Comparing prevalence estimates across studies was difficult due to differences in assessment methods and samples. Therefore, prevalence studies need to apply well-defined diagnostic criteria	Moderate (7/9)
Kazda et al. [17]	Systematically evaluate, and synthesize the evidence on overdiagnosis of ADHD in children and adolescents utilizing published 5-question framework for detecting over diagnosis in noncancer conditions	Overdiagnosis	1979–August 2020	334	Not given	Narrative Synthesis	– One-third of the studies were of high quality – Substantial evidence of a reservoir of ADHD was reported by 104 studies, which suggest that number of diagnosis could increase in future – 45 studies provided an evidence that the actual ADHD diagnosis had increased – 25 studies reported that these additional cases may be on the milder end of the ADHD spectrum, and – 83 studies suggested that pharmacological treatment of ADHD was increasing	High quality studies are needed to identify the long-term benefits and harms of diagnosing and treating ADHD in young people with milder symptoms	Moderate (7/9)
**Adults**									
Song et al. [18]	Assess the global prevalence of adult ADHD in the general population	Prevalence, Worldwide	January 2000–December 2019	40	107,282 for persistent adult ADHD and 50,098 for symptomatic adult ADHD	Random-effect meta-analysis	– Majority of studies were of moderate to high quality – Based on data published from 2005 to 2019, the pooled prevalence was 4.61% (3.41–5.99) and 8.83% (7.23–10.57) for persistent and symptomatic adult ADHD respectively – The most common age group for adult ADHD cases was 18–24 – LMICs showed a higher prevalence of persistent adult ADHD than HICs, while WHO regions showed different rates of symptomatic adult ADHD – Significant heterogeneity (*I* ^2^ > 97%) were observed across analyses	To better understand the global epidemiology of both persistent and symptomatic adult ADHD, there is still a need for well-defined diagnostic procedures and more large-scale international studies with minimal methodological heterogeneity	High (9/9)
Dobrosavljevic et al. [19]	Assess ADHD prevalence among older adults ≥50 according to different assessment methods	Prevalence, Older adults	Database inception – June 2020	20	20,999,871	Random-effect meta-analysis	– Included studies were of moderate quality – The prevalence of ADHD was higher 2.18% (1.51–3.16) for validated scales in community sample compared to the prevalence assessed based on clinical diagnosis 0.23% (0.12–0.43), and treatment 0.09% (0.06–0.15) – Significant heterogeneity (*I* ^2^ > 87%) were observed across analyses	Prevalence studies in community samples should use more comprehensive assessment tools to explore whether individuals with elevated ADHD symptoms meet established diagnostic criteria. They should examine the possible reasons behind high levels of ADHD symptoms reported via validated scales	Moderate (7/9)

Abbreviations: DSM, diagnostic statistic model; HICs, high-income countries; *k*, total number of primary studies included; LMICs, lower and middle-income countries; WHO, World Health Organization.

a Total participants included in the systematic review and meta-analysis or otherwise specified.

b For findings from meta-analysis, if given effect estimates with 95% CI are presented or otherwise specified.

### Genetic and environmental risk factors associated with ADHD (n = 41, Table 5)

In this section, we use terms like risk, correlation, protective, and association according to reports in the systematic reviews, without indicating causality. Generally, this literature did not bring evidence for conclusions on causality, which will be discussed later.

**Table 5.** Genetics and environmental risk factors associated with ADHD

References	Objective	Keywords	Timeframe of database search	*k*	Sample size^a^	Analytical design	Main findings (Effect estimates, 95% CI)^b^	Conclusion and comments from the authors	Quality assessment
Ronald et al. [20]	Review if ADHD polygenic risk score is associated with ADHD and related traits	Genetics, Polygenic risk score	Not given	44	>14,000	Narrative synthesis	– Majority (80%) of included studies were of high quality – Strong evidence was found for the of associations between ADHD PRS and ADHD, ADHD traits, brain structure, education, externalizing behaviors, neuropsychological constructs, physical health, and socioeconomic status – PRS associated with ADHD had an OR of 1.22 to 1.76%; variance explained in dimensional assessments of ADHD traits ranged from 0.7 to 3.3%	The review suggest that the ADHD PRS is robust and reliable, associating not only with ADHD but many outcomes and challenges known to be linked to ADHD	High (8/9)
**Prenatal factors**									
Li et al. [21]	Clarify the association between maternal pre-pregnancy overweight/obesity and risk of ADHD in offspring	Obesity, Confounding	1975–2018	14 (8 in meta-analysis)	784, 804 mother–child pairs	Random-effect meta-analysis	– Included studies were of moderate to high quality – Maternal overweight (RR = 1.31,1.25–1.38) and obesity (RR = 1.92, 1.84–2.00) both increased the risk of ADHD in offspring – However, the association was significantly attributable to unmeasured familial confounding and was not a causal – No significant heterogeneity were observed across analysis	Future studies using robust methodological design that considers unmeasured familial confounders, and genetic and environmental origin of such confounders and includes various populations are required	High (8/9)
Ai et al. [22]	Assess the association between antibiotic exposure and the risk of ADHD in childhood	Antibiotic ADHD, Microbiome	Database inception – January 2021	11	2,238,348	Random effects meta-analysis	Included studies were of moderate to high quality – Maternal antibiotic exposure during pregnancy was associated with an increased risk of ADHD in offsprings (OR = 1.14; 1.10–1.18) – The included studies had insufficient adjustment for confounders – Moderate heterogeneity (*I* ^2^ = 64%) was observed across analysis	Future research should examine whether different types, courses, and durations of antibiotic use affect ADHD risk and adjust for potential confounders	Moderate (7/9)
Gou et al. [23]	Evaluate the association between maternal acetaminophen use during pregnancy and the risk of ADHD in children	Acetaminophen, Pregnancy	Database inception – November 2018	8	244,940	Random-effect meta-analysis	– Majority of included studies were of high quality – There was an association between maternal acetaminophen use during pregnancy and the risk of ADHD in offspring (Rrat = 1.25, 1.17–1.34) with a greater risk among offspring who were exposed to acetaminophen at the third trimester and for longer duration, that is, 28 days or more – No significant heterogeneity were observed across analyses	Caution must be taken while interpreting the result as this observed association might be due to potentially unidentified or inadequately controlled confounders. Hence, further studies are required	Moderate (7/9)
Man et al. [24]	Assess the association between antidepressant exposure during pregnancy and ADHD in offspring	Antidepressant, Pregnancy	January 1946–July 2017	8	2,886,502	Random-effect meta-analysis	– Included studies were of high quality – Increased risk of ADHD among those with prenatal exposure to antidepressants compared to nonexposure was observed (Rrat = 1.39, 1.21–1.64) – However, the meta-analysis result of three studies that used sibling-matched analyses yielded a nonsignificant association – No significant heterogeneity were observed across analyses	Association between prenatal antidepressants exposure and ADHD in children is likely to be confounded by other factors	High (9/9)
Dan et al. [25]	Assess the relationship between maternal pregestational or gestational diabetes and occurrence of ADHD in children	Maternal diabetes, ADHD, offspring	Database inception – January 2019	7	3,169,529 out of which, 148,374 children exposed to maternal diabetes, and 3,021,155 belonging to the reference	Random-effect meta-analysis	– Majority of the studies were of moderate quality – Maternal pregestational diabetes increased the risk of ADHD in offspring by 44% (1.32–1.57) with no significant heterogeneity – -However, no association was observed between gestational diabetes and ADHD (RR = 1.19, 0.99–1.42) with moderate heterogeneity (*I* ^2^ = 68.7%)	Given the limited availability of reliable information, further cohort studies are required to assess this relationship more comprehensively	Moderate (7/9)
Rowland et al. [26]	Explore the association between gestational diabetes and ADHD	Pregnancy, Gestational diabetes	Database inception – April 2021	15	Children of 132,458 mothers with gestational diabetes, 857,623 control. 401 children with ADHD, 1,828 controls	Random effects meta-analysis	– Majority of included studies were of poor quality – No significant difference was observed between children of mothers with gestational diabetes and controls – Moderate heterogeneity (*I* ^2^ > 45%) was observed across analysis	Not mention for ADHD	High (9/9)
Dong et al. [27]	Assess the association between prenatal exposure to MSDP and ADHD in offspring	Prenatal exposure, Maternal smoking during pregnancy	Database inception – June 2017	27	3,076,173	Random-effect meta-analysis	– Included studies were of high quality – Significant association was observed between prenatal exposure to MSDP or maternal smoking cessation during the first trimester and ADHD in offspring, while there was no such association for maternal smoking cessation before pregnancy – Significant heterogeneity (*I* ^2^ > 80%) was observed across analysis for MSDP	More studies with robust designs, more effective exposure assessment, and large sample size are needed to clarify the causal relationship between MSDP and ADHD in offspring	High (8/9)
Schwartz et al. [28]	Determine the association between POE and ADHD symptoms in children and adolescents	Prenatal opioid exposure	January 1950–October 2019	7	319 children with POE and 1,308 nonexposed children	Random-effect meta-analysis	– POE was associated with higher rates of hyperactivity (SMD:1.4, 0.49–2.31), inattention (SMD: 1.35, 0.69–2.01) and combined ADHD symptoms scores (SMD: 1.27, 0.79–1.75) – POE was associated with ADHD symptoms both at preschool and school age – Significant heterogeneity (*I* ^2^ > 87%) were observed across analyses	Future research should clarify the relationship between biological, environmental, and social risk factors, respectively, and ADHD symptoms in children with POE	Moderate (7/9)
Qu et al. [29]	Explore the relationship between maternal exposure to perfluoroalkyl substances and early ADHD in children	Perfluoroalkyl substances, Maternal exposure	Database inception – October 2020	15 (9 in meta-analysis	17,565	Narrative synthesis and random effects meta-analysis	– Majority of included studies were of high quality – No statistical significant differences was found for early ADHD and exposure to perfluoroalkyl substances (perfluorooctanoic acidperfluorooctane sulfonate, perfluorohexane sulfonate, perfluorononanoic acid, perfluorodecanoic acid) – Subgroup analysis showed a positive association between PFOS concentration in children’s blood and early ADHD (OR = 1.05, 1.02–1.08) – Subgroup analysis showed a positive association between perfluorononanoic acid level in maternal blood and ADHD in children (OR = 1.42, 1.04–1.81) – Subgroup analysis showed a positive association between perfluorooctane sulfonate level and ADHD in children in America (OR = 1.05, 1.02–1.08 – Moderate to significant (*I* ^2^ > 54.7–87.2) heterogeneity were observed across analyses before subgroup analysis	To better understand the pathogenesis of ADHD, epidemiological studies are needed in several regions, particularly on PFAS exposure types	Moderate (7/9)
**Perinatal factors**									
Zhu et al. [30]	Evaluate the association between perinatal hypoxic–ischemic conditions and future ADHD	Hypoxia, ischemia, Risk factor	Database inception – before September 2015	10	Cases: 45,821 Controls: 9 2,07,363	Fixed or random-effect meta-analysis	– Included studies were of moderate quality – Perinatal hypoxic–ischemic conditions like preeclampsia (OR = 1.31, 1.26–1.37), Apgar score < 7 at 5 minutes (OR = 1.31,1.12–1.54), breech/transverse presentations (OR = 1.14,1.06–1.23), and prolapsed nuchal cord (OR = 1.10,1.06 – 1.15) were all associated with increased risk of future ADHD – Moderate heterogeneity (*I* ^2^ = 63%) was observed for analysis of Apgar score < 7 at 5 minutes	Given the limited number of studies, more well-designed studies are needed to confirm this association	High (8/9)
Franz et al. [31]	Determine the association between preterm and LBW and future ADHD, both for categorical diagnosis and dimensional symptomatology, compared with controls	Very Preterm, Very Low Birth Weight, Extreme Preterm, Extreme Low Birth Weight	Database inception – 2017	34	5,291	Random-effect meta-analysis	– Included studies were of moderate quality – VP/VLBW and EP/ELBW individuals were at an increased risk of a categorical diagnosis of ADHD with highest OR for the most extreme cases – This was supported by a meta-analysis based on ADHD symptomatology showing a significant association for VP/VLBW and symptoms of inattention (SMD = 1.31,0.66–1.96), hyperactivity and impulsivity (SMD = 0.74, 0.35–1.13), and combined (SMD = 0.55, 0.42–0.68) as compared to controls – Significant heterogeneity (*I* ^2^ > 90%) was observed across analysis except for the combined dimension in the VP/VLBW group (moderate *I* ^2^ = 54%)	Specific causal determinants associated with prematurity and LBW and subsequent ADHD needs to be studied	High (9/9)
Serati et al. [32]	Review the literature on obstetric and neonatal complications and future risk of ADHD	Perinatal complications, Child development	Database inception – December 2016	40	57–1,772,548 per study	Narrative synthesis	– Majority of included studies were of moderate quality – LBW (Cohen’s *d* effect size range = 0.31–1.64) and preterm birth (range *d* = 0.41–0.68) were identified as an important risk factors for future ADHD	PB and LBW children should be carefully monitored for an early diagnosis of ADHD	Moderate (6/9)
Curran et al. [33]	Investigate the impact of CS compared to vaginal delivery and the odds of subsequent ADHD in children	ADHD, Cesarean section	Database inception – February 2014	4	Not given	Random-effect meta-analysis	– Included studies were of poor to moderate quality – Unadjusted estimates showed a positive association between CS and ADHD in offspring – However, the only two studies reporting adjusted risk estimates showed that this relation was not significant (OR = 1.07,0.86–1.33) – No heterogeneity were observed across analyses	Included studies were unable to provide a clear description of the possible association. Hence, future studies need to adjust for potential key confounders and effect modifiers (type of CS, sex of the child, maternal obesity, socioeconomic status, maternal age) to assess the relationship between CS delivery and ADHD	Moderate (7/9)
Xu et al. [34]	Examine the association between CS and ADHD in children	ADHD, Cesarean section, Confounders	Database inception – December 2018	9	More than 2.5 million	Fixed or random‐effect meta-analysis	– Included studies were of high quality – CS was associated with a small increase in the later risk of ADHD in offspring (OR = 1.14, 1.11–1.17) – However, the meta-analysis result of data from sibling analyses showed the association as marginally significant (OR = 1.06, 1.00–1.13), suggesting the association was due to confounders – No heterogeneity were observed across analyses	The observed association may have been overestimated as the included primary studies could not control for important confounding factors. Hence, more prospective cohort studies with large sample size and data on potential confounders/predictors are required	High (8/9)
Min et al. [35]	Explore the association between parental age at delivery and ADHD risk in offspring	Parental age; Children	Database inception – April 2021	11	Cases: 111,101 Controls: 4,306,047	Random effects meta-analysis	– Majority of included studies were of moderate quality – Compared with the reference groups, the lowest parental age category was associated with an increased risk of ADHD in the offspring (OR = 1.49, 1.19–1.87) and (OR = 1.75,1.31–2.36) for mother and father, respectively – No significant association was found between the highest parental age and ADHD – Significant heterogeneity (*I* ^2^ > 95%) were observed across analyses	A causal relationship and mechanisms between parental age and the risk of ADHD in offspring need to be explored in future research	High(8/9)
**Postnatal factors**									
Tseng et al. [36]	Examine the relationship between breastfeeding and ADHD in children, taking into account of important factors such as the duration and methods	Breastfeeding, Nutrition, Risk	Database inception – September 2017	11	Cases: 4,107 Controls: 90,392	Random effects meta-analysis	– Majority of included studies were of moderate quality – Children with ADHD had significantly less breastfeeding duration than controls (Hedges’ g = −0.36, −0.61 to –0.11) – Association was found between nonbreastfeeding and ADHD children (ajOR = 3.71, 1.94–7.11) – Moderate heterogeneity (*I* ^2^ = 42.5%) was observed across analysis	Additional longitudinal studies are needed to confirm/refute the association and to explore possible mechanisms underlying this association	Moderate (7/9)
Zeng et al. [37]	Investigate the association between maternal breastfeeding and ADHD in offspring	Breastfeeding, Nutrition, Protective factor	Unclear	12	ADHD Cases: 3,686 Controls: 106,907	Random-effect meta-analysis	– Majority of included studies were of moderate quality – Maternal breastfeeding (of any duration) may reduce the risk of future ADHD in children (OR = 0.70, 0.52–0.93) compared to those who were never breastfed – Moderate heterogeneity (*I* ^2^ > 70%) was observed across analysis	Prospective studies should adjust for potential confounders and apply standardized diagnostic methods to examine the causal relationship	Moderate (6/9)
Huang et al. [38]	Explore the association between postnatal exposure to SHS and future risk of ADHD	Second-hand smoking, Postnatal	Database inception – January 2020	9	ADHD cases: 6,663 Controls: 93,825	Random-effect meta-analysis	– Majority of included studies were of high quality – Children exposed to SHS were found to be at the increased risk of ADHD (OR = 1.60, 1.37–1.87) – Moderate heterogeneity (*I* ^2^ = 42.5%) was observed across analysis	Causal relationship between SHS and ADHD needs to be determined by fully adjusting for potential confounders	Moderate (7/9)
**Environmental factors**									
Asarnow et al. [39]	Investigate ADHD diagnoses in children and adolescents following concussions and mild, moderate, or severe TBI	Injury, Traumatic Brain Injury	1981–December 2019	24	TBI cases: 12,374 Controls: 43,491	Random-effect meta-analysis	– Majority of included studies were of moderate quality – Children with TBI were at increased risk of getting ADHD compared with those with other injuries (1 year: OR = 4.81,CrI: 1.66–11.03) and (>1 year OR = 6.70,2.02–16.82) and noninjured controls (1 year OR = 2.6, 0.7–6.6), and (>1 year OR = 6.25, 2.06–15.04), as well as those with mild TBI (1 year OR = 5.69,1.46–15.67), (>1 year OR: 6.65, 2.14–16.44) – Of 5, 920 children with severe TBI, 35.5% had ADHD more than 1 year postinjury	Further studies with large sample size particularly for concussion and other injuries are needed	Moderate (6/9)
Sun et al. [40]	Examine the evidence for a relationship between general anesthesia induced in childhood and the risk of ADHD	Attention, Behavior, Cognitive, Surgery, Anesthesia	Database inception – October 2020	7	ADHD cases: 49,141 Controls: 251, 246	Random effects meta-analysis	– Majority of included studies were of high quality – Exposure to general anesthesia in childhood was associated with a risk of ADHD in later life (RR = 1.24,1.11–1.38) – Subgroup analysis showed that a single anesthetic exposure was not associated with risk for ADHD – Two or more exposures was associated with risk for ADHD – Moderate heterogeneity (*I* ^2^ = 74.8%) was observed for two or more exposure	To confirm these findings, more prospective cohort studies with larger sample sizes are needed	High (9/9)
Daneshparvar et al. [41]	Review relevant literature related to lead exposure and ADHD symptoms in children	Blood Lead Level, Lead Poisoning	Database inception – May 2014	18	12,195	Narrative synthesis	– Majority of included studies were of high quality – Blood lead level of < 10 g/dL have a significant effect on at least one ADHD subtype	Recommends revising the present threshold (less than 10 g/dL) for permissive blood levels and measuring the BLL in children to reduce the harm caused by prolonged exposure to lead	Moderate (6/9)
Kalantary et al. [42]	Examine the association between ADHD symptoms and exposure to PAH exposure during the prenatal and postnatal periods in children of nonsmoking mothers	PAHs, Children	Database inception – September 2018	6	2,799	Fixed and random-effect meta-analysis	– Majority of included studies were of high quality – Although four of six studies (all by the same author) found a significant association between PAH exposure and later ADHD, no association was observed between prenatal and postnatal exposure to PAH and future ADHD in children when including adjusted analyses from all the six included studies (OR = 1.99,0.96–4.11) – No heterogeneity was observed across analysis	Additional research needs to be conducted in different countries	Moderate (7/9)
Zhang et al. [43]	Determine the association between exposure to air pollutants and development of ADHD in children	PAHs, Nox, Particulate matter	Unclear	9	>98,000	Random-effect meta-analysis	– Majority of included studies were of high quality – No significant association was found between exposure to PAHs (RR = 0.98, 0.82–1.17), NOx (RR = 1.04, 0.94–1.15, and PM (RR = 1.11, 0.93–1.33) and an increased risk of ADHD in children – Moderate heterogeneity (*I* ^2^ = 60.1%) was observed NOx and PM	Further prospective studies to determine causal relationship between exposure to particles and ADHD are needed	High (8/9)
Aghaei et al. [44]	Synthesize relationship between exposure to air pollutants and risk of ADHD in children	Ambient air pollutants, particulate matter, ADHD	Database inception – 2018	28	140,159	Narrative synthesis	– Due to the significant variation in methodology used in included studies, no firm conclusion can be drawn about the exposure to ambient gaseous and particulate matters and the risk of ADHD in children	Further studies need to apply accurate exposure and outcome assessment method and consider all possible confounders	High (8/9)
Lam et al. [45]	Assess association between developmental exposure to PBDE and ADHD	Polybrominated diphenyl ether, ADHD	Database inception – September 2016	9	62–622 mother–child pairs	Narrative synthesis	– Due to limited data, no firm conclusion can be drawn about the exposure to PBDE and the risk of ADHD in children	Further studies of good quality are needed	High (9/9)
Caye et al. [46]	Determine the relationship between age and ADHD diagnosis	Relative age, Immaturity	Database inception – December 2018	25	8,076,570	Random-effect meta-analysis	– Majority of the included studies were of high quality – Children born in the last 4 months of the school calendar year were at higher risk of receiving ADHD diagnosis compared to their relatively older class peers (RR = 1.34,1.26–1.43) – Significant heterogeneity (*I* ^2^ = 96.7%) was observed across analysis	Relative maturity and developmental age should be consistently considered while making an ADHD diagnosis	High (9/9)
Russell et al. [47]	Assess the association between SES and ADHD	Socioeconomic status, Health inequalities	1999–2013	42	53 to 842 per study	Random-effect meta-analysis	– The association between socioeconomic disadvantage and ADHD was a consistent finding, but can be mediated by parental mental health, maternal smoking during pregnancy, or other risk factors that are more prevalent in families with low SES	Further research that considers possible mechanism between SES and ADHD is warranted	Moderate (7/9)
Langevin et al. [48]	Disentangle the association between CSA and ADHD	Child sexual abuse, Predictor	Database inception – January 2020	28	75,306	Narrative synthesis	– Variation in quality of included studies and lack of longitudinal studies restricted from untangling the association between CSA and ADHD	Rigorous longitudinal studies including those that assess the confounding role of other maltreatment forms and trauma-related symptoms are needed	Moderate (7/9)
Del-Ponte et al. [49]	Examine the association between dietary patterns and ADHD	Diet, Dietary pattern	Unclear	14	375–16,831 participants per study	Random-effect meta-analysis	– Majority of included studies were of moderate quality – Children and adolescents consuming healthy diets have lower odds of having ADHD (OR = 0.65, 0.44–0.97) compared to those consuming unhealthy diet (OR = 1.41, 1.15–1.74) – Moderate heterogeneity (*I* ^2^ > 73%) were observed across analysis	Longitudinal studies are needed to strengthen the evidence about the relationship between diet and ADHD	Moderate (6/9)
Farsad-Naeimi et al. [50]	Determine relationship between sugar consumption and the development of ADHD symptoms	Sugar, Soft drink	Database inception – March 2020	7	25,945	Fixed- and random-effect meta-analysis	– Majority of included studies were of high quality – A positive relationship was found between sugar and soft drink consumption and ADHD (*d* = 1.27, 1.02–1.42) – Significant heterogeneity (*I* ^2^ = 81.9%) was observed across analysis	Longitudinal studies that examine a potential causal relationship between sugar and soft drink consumption, and ADHD are needed	High (8/9)
Khashbakht et al. [51]	Synthesize the available literature on the relation between vitamin D status and ADHD	Vitamin D, Children, Adolescents	Database inception – June 2017	13	Cases: 33–1,331 Controls: 20–6,492; Cohort:3,733	Random-effect meta-analysis	– Majority of included studies were of moderate quality – Children and adolescents with ADHD had a lower mean concentration of serum 25 (OH)D than healthy controls – The studies which reported ORs also showed a significant association between lower vitamin D status and ADHD (OR = 2.57; 1.09–6.04) – Further, a meta-analysis from prospective studies also showed an inverse relationship between perinatal vitamin D and ADHD (RR = 1.40, 1.09–1.81) – Significant heterogeneity (*I* ^2^ > 80%) was observed across analysis	To understand the causal association between vitamin D status and the risk of developing ADHD, prospective cohort studies with large sample size, including different population and even population-based intervention studies that consider the maximum number of possible confounders should be conducted	High (8/9)
La Chance et al. [52]	Estimate the relationship between blood ratio of Omega-6 and Omega-3 fatty acids (n6/n3) and AA/EPA, to ADHD symptoms	Omega-3 fatty acids, Omega-6 fatty acids	Database inception – April 2014	5	Not given	Random-effect meta-analysis	– Included studies were of high quality – Children and youth with ADHD have higher n6/n3 fatty acid ratios (SMD = 1.97,0.90–3.0)4, and higher AA/EPA ratios (SMD = 8.25,5.94–10.56) than those in controls – Significant heterogeneity (*I* ^2^ = 83%) was observed for n6/n3 fatty acid ratios	Future research could assess whether fatty acid ratios could be used as biomarkers to identify which children with ADHD who may specifically benefit from treatment with essential fatty acids to normalize the n6/n3 or AA/EPA ratios	High (8/9)
Huang et al. [53]	Determine the relationship between magnesium level and ADHD in children	Magnesium, Trace element, Nutrition	Database inception – October 2018	12	Cases: 2,872 Controls: 2,838	Random-effect meta-analysis	– Included studies were of high quality – ADHD children have significantly lower peripheral blood magnesium level (Hedges’ *g* = −0.5,−0.8 to −0.2) and lower hair magnesium levels (Hedges’ *g* = −0.7,−1.3 to −0.1) compared to controls – Significant heterogeneity (*I* ^2^ = 85%) was observed for hair magnesium levels	Prospective studies with a large sample size are required to determine the causal relationship between magnesium level and the pathophysiology of ADHD	High (8/9)
Effatpanah et al. [54]	Examine the relationship between magnesium status and ADHD	Magnesium, Trace element	Database inception – August 2018	7	Cases: 9–1,331 Controls: 11–1,331	Random-effect meta-analysis	– Included studies were of moderate quality – Children and adolescents with ADHD have 0.10 mmol/l (−0.18−0.02) lower serum magnesium levels than controls indicating an inverse relationship between magnesium level and ADHD – Significant heterogeneity (*I* ^2^ > 95%) was observed across analysis	Further observational studies using standard diagnostic measurement are required to draw meaningful conclusions about magnesium level and ADHD	High (9/9)
Shih et al. [55]	Assess the association between peripheral manganese level and ADHD	Manganese, Pediatric psychiatry	Database inception – March 2018	4	Cases: 175 Controls: 999	Random-effect meta-analysis	– Included studies were of moderate quality – Meta-analysis found significantly higher peripheral manganese levels in ADHD children compared to controls when including studies with either blood or hair levels) (Hedges’ *g* = 0.30,0.02–0.58) – However, the association was no longer significant when only blood manganese level was analyzed separately (Hedges’ *g* = 0.32, −0.03 − 0.69) – Moderate heterogeneity (*I* ^2^ > 50%) was observed across analysis	Additional primary studies are warranted to understand better the relationship between peripheral manganese level and ADHD	Moderate (7/9)
Degremont et al. [56]	Examine whether children with ADHD have lower serum and brain iron concentrations, compared with healthy control subjects	Brain iron, Serum iron, Iron status, Serum ferritin	2000–June 2019	20	Cases: 2,209 Controls: 2,982	Narrative synthesis	– Majority of included studies were of moderate quality – Serum ferritin concentrations varied between studies, with 10 of 18 studies finding higher concentration in patients with ADHD compared to healthy controls – For serum iron, 7 of 10 studies showed no difference, 2 studies showed lower concentrations in patients with ADHD, and 1 study showed higher concentration. – 3 studies reported lower brain iron in patients with ADHD – Study methods and participants were heterogeneous	There is need for longitudinal studies and larger MRI studies using magnetic field correlation to measure brain iron concentration in different regions of the brain	Moderate (7/9)
Cortese et al. [57]	Summarize the evidence about iron level and ADHD	Iron, Ferritin, Trace element	Database inception – July 2012	22	500–2,000	Narrative synthesis	– Most of the studies assessing iron status in children with ADHD used serum ferritin as a measure, with mixed findings (i.e., both significant and nonsignificant associations)	More research based on other measures for assessment than serum ferritin are required to elucidate the relationship between iron status and ADHD	Moderate (7/9)
Tseng et al. [58]	Identify the association between iron status and ADHD	Iron, Ferritin	Database inception – August 2017	17	>10,000	Random-effect meta-analysis	– Majority of included studies were of high quality – Children with ADHD have lower peripheral serum ferritin levels (Hedges’ *g* = −0.24,−0.44 to −0.05), but not iron or transferrin levels compared to healthy controls – Children with iron deficiency have more severe ADHD symptoms than ADHD children without ID (Hedges’ *g* = 0.88, 0.32–1.45) – Significant heterogeneity (*I* ^2^ = 82.9%) was observed for peripheral serum ferritin level	Prospective studies are required to provide better insight into the relationships between iron status and ADHD symptoms and to clarify the potential pathophysiological mechanisms	High (8/9)
Ghoreisy et al. [59]	Estimate the association between hair and serum/plasma zinc levels and ADHD	Zinc, Trace elements	Database inception – October 2020	22	Cases: 1,280 Controls: 1,200	Random effects meta-analysis	– Majority of included studies were of high quality – Serum or plasma zinc levels in subjects with ADHD were not statistically different compared to controls (WMD = − 1.26 μmol/L,−3.72–1.20) – After removing one study which showed a significant higher levels of serum/plasma zinc in subjects with ADHD compared to the controls, zinc levels were lower in ADHD patients (WMD = − 2.49 μmol/L,−4.29 to −0.69) – Further, hair zinc levels in cases with ADHD were not statistically different compared to controls (WMD: − 24.19 μg/g; −61.80 – 13.42) – Significant heterogeneity (*I* ^2^ > 98%) were observed across analyses	Further well-designed studies are needed to clarify the role of zinc in the etiology of ADHD	High (8/9)
Luo et al. [60]	Explore the available evidence on the correlation between zinc and ADHD	Zinc, ADHD	Database inception – April 2019	11	1,517	Random-effect meta-analysis	– Majority of the included studies were of good quality – No significant difference in zinc level in blood (SMD = −0.91,−1.88–0.07) or hair (SMD = 1.4,−4.49–7.33) was found between children and adolescents with and without ADHD – Significant heterogeneity (*I* ^2^ > 95%) were observed across analysis	Additional studies with a large sample size and robust methodology are required	Moderate (7/9)

Abbreviations: AA, arachidonic acid; CrI, credible interval; CS, cesarean section; CSA, child sexual abuse; *d*, effect size; EA, eicosapentaenoic acid; ID, iron deficiency; *k*, total number of included studies; MSDP, maternal smoking during pregnancy; Nox, nitrogen oxides; OR, odds ratio; PAH, polycyclic aromatic hydrocarbon; PB, preterm birth; PBDE, polybrominated diphenyl ether; PM, particulate matter; POE, prenatal opioid exposure; RR, relative risk; Rrat, risk ratio; SES, socioeconomic status; SHS, second-hand smoke; SMD, standardized mean difference; TBI, traumatic brain injury; VLBW/ELBW, very/extreme low birth weight; VP/EP, very and extreme preterm; WMD, weighted mean difference.

a Total participants included in the systematic review and meta-analysis or otherwise specified.

b For findings from meta-analysis, if given effect estimates with 95% CI are presented or otherwise specified.

#### Genetic factors

One systematic review found strong evidence that the common genetic variants underlying ADHD, as measured by the ADHD polygenic risk score, were associated not only with diagnosed ADHD but also with more dimensional ADHD traits [20].

#### Maternal factors

Maternal pre-pregnancy overweight [21], use of antibiotics [22], acetaminophen [23], and antidepressants [24] during pregnancy, and maternal pregestational diabetes [25], but not gestational diabetes [26], were associated with increased rates of ADHD in offspring. However, the authors stated that associations may be due to unmeasured confounding and thus not causal (e.g., the association with maternal overweight was explained by familial confounding). Maternal smoking during pregnancy (odds ratio (OR) = 1.56, 95% CI: 1.41–1.72) or smoking cessation during the first trimester was associated with ADHD in offspring [27]. Similarly, prenatal opioid exposure was associated with higher ADHD symptom scores (standardized mean difference (SMD) =1.27, 0.79–1.75) [28]. Maternal exposure to perfluoroalkyl substances was not associated with ADHD in their children [29].

Perinatal complications like maternal preeclampsia [30], very preterm birth/very low birth weight (OR = 3.04, 95% CI: 2.19–4.21) [31], or low birth weight [32] were associated with offspring ADHD. Likewise, the caesarian section was reported to be associated with later ADHD diagnosis in unadjusted analyses [33], but a later review reported this association to be partly or entirely accounted for by residual confounding [34].

A nonlinear relation between parental age and risk for ADHD in the offspring was found with the highest risk for parents below 20 years and lowest risk for parents in the mid-thirties [35].

While maternal breastfeeding was associated with a reduced risk of ADHD in children [36, 37], postnatal exposure to second-hand smoking was associated with increased risk (OR = 1.60, 95% CI: 1.30–1.80) [38].

Compared to children with other injuries or without injuries, children with severe traumatic brain injuries had an increased risk of being diagnosed with ADHD at less or more than 1 year, respectively, after the injuries [39]. Likewise, two or more exposures to general anesthesia were associated with an increased risk of ADHD in later life (relative risk RR = 1.84, 1.14–2.97) [40]. Blood lead level was associated with higher ADHD rates in children and adolescents [41]. No significant association was found between polycyclic aromatic hydrocarbon exposure and ADHD in children [42, 43], and there were inconclusive evidence for an association between exposure to air pollution [44] or polybrominated diphenyl ethers [45] and ADHD.

According to two included reviews, children being relatively younger than classmates had higher rates of ADHD diagnosis [17, 46], with one reporting the relative risk of 1.34 (95% CI: 1.26–1.43) for the youngest children [46]. Further, two reviews suggested an association between socioeconomic disadvantage and risk of ADHD [15, 47], with one suggesting it to be mediated by factors such as parental mental health and maternal smoking during pregnancy [47]. The evidence regarding child sexual abuse as a predictor for ADHD was unclear [48].

#### Dietary pattern, nutrition, and trace elements

Children and adolescents consuming healthy diet had lower risk of having ADHD compared to those consuming unhealthy diet [49]. Positive relationship was indicated between total sugar intake from soft drinks and dietary sources and ADHD symptoms in children and adolescents [50]. Other nutritional factors associated with higher rates of ADHD among children and adolescents were low serum concentration of 25-hydroxyvitamin D, lower perinatal and childhood vitamin D status [51], elevated ratios of both blood omega-6 to omega-3 and arachidonic acid to eicosapentaenoic acid fatty acids [52]. Two systematic reviews reported significantly lower serum manganese levels in children with ADHD [53, 54]. Another review revealed higher peripheral manganese levels in both blood and hair in children and adolescents with ADHD compared to healthy controls [55].

A review from 2021 suggested that brain iron concentrations, specifically in the thalamus, were lower in children with ADHD than in healthy controls [56]. However, mixed results were reported for systemic iron level [56, 57]. In contrast, a review from 2018 concluded that low serum iron levels were associated with ADHD [58]. There was no difference in zinc levels in blood, serum, plasma [59] or hair between children and adolescents with ADHD and healthy controls [60].

### Long-term prognosis and life trajectories in ADHD (n = 19, Table 6)

#### Education and employment

ADHD was associated with lower educational attainment [61], including failure to complete high school (OR = 3.7, 95% CI: 2.0–7.0) and failure to attend tertiary education (OR = 6.47, 4.58–9.14) [62]. Further, a negative association was found between ADHD and mathematical ability [63]. Individuals with ADHD were more prone to experience occupational challenges [61], for example, they were more often dismissed from work (OR = 3.92, 2.68–5.74), unemployed (OR = 1.97, 1.01–3.85) [62], and more likely to receive public welfare payments [61].

#### Alcohol, smoking, and substance use

Childhood ADHD was significantly associated with alcohol use disorder [64], including the development of alcohol use disorder by early adulthood (OR = 1.35, 95% CI: 1.11–1.64) and nicotine use by middle adolescence (OR = 2.36, 1.71–3.27) [65]. Smokers with ADHD in childhood smoked significantly more cigarettes as adolescents than smokers without childhood ADHD [66]. There was an association between ADHD and substance use disorder [64], with an earlier meta-analysis reporting an odds ratio of 1.73(1.24–2.41) [62]. The estimated average prevalence of cocaine use in adults with ADHD was 26.0% (18.0–35.0) [67].

#### Injuries, poisoning, and suicidal spectrum behavior

Both children and adults with ADHD were at higher risk of injuries (OR = 1.96, 95% CI: 1.63–2.37) [68], including bone fracture [69] and unintentional physical injuries (OR = 1.53, 1.40–1.67) [70], but not for sports-related concussions [71]. Children and adolescents with ADHD also had a higher risk of poisoning than controls (RR = 3.14, 2.23–4.42) [72]. Suicidal spectrum behavior was higher in ADHD, including suicidal ideations (OR = 3.53, 2.94–4.25), suicidal plans, attempts (OR = 2.37, 1.64–3.43), and completed suicide [73].

#### Psychotic disorders

Childhood ADHD was associated with an increased risk of subsequent psychotic disorders (OR = 4.74, 95% CI: 4.11–5.46) [74].

#### Criminal offenses and domestic violence

The pooled prevalence of ADHD among individuals in detention settings was 26.2% (95% CI: 22.7–29.6) [75]. There was a significant association between childhood ADHD and adolescent and adulthood arrests (RR = 2.2, 1.3–3.5), convictions (RR = 3.3, 2.1–5.2), and incarcerations (RR = 2.9, 1.9–4.3) with a younger age of onset of antisocial involvement and an increased risk of criminal recidivism [76]. However, there was no conclusive evidence for an association between ADHD and domestic violence [77].

#### Pregnancies and postpartum risk

Adolescent girls with ADHD had an increased risk of teenage and unintended pregnancies, and women with ADHD had a higher risk of pregnancy and birth complications, such as pre-eclampsia, infection, and cesarean section [78].

#### Global economic burden of ADHD

The per person annual economic burden of ADHD ranged from $US832 to 20,539 for patients with ADHD, and from $US2,670 to 4,120 for family members of patients with ADHD [79].

**Table 6.** Long-term prognosis and life-trajectories in ADHD

References	Objective	Keywords	Timeframe of database search	*k*	Sample size^a^	Analytical design	Main findings (Effect estimates; 95% CI)^b^	Conclusion and comments from the authors	Quality assessment
Christiansen et al. [61]	Synthesize evidence of an association between ADHD diagnosis in childhood and later education, earnings and employment, compared to children without an ADHD diagnosis	Life-course, Occupation, Work, Employment	Database inception – November 2020	6	Cases: 1,380 Controls: 888	Narrative synthesis	– Majority of the included studies were of moderate quality-ADHD was associated with lower quality employment, lower lifetime income, and more part time and unskilled work and with lower educational attainment measured in several ways	There is a need for further high-quality research evaluating factors and interventions that reduce the long-term vocational impacts of childhood ADHD, especially ADD, which is not addressed in the present literature	High (9/9)
Erskine et al. [62]	Explore the potential outcomes associated with ADHD diagnosis	ADHD, Long-term prognosis	1980–March 2015	101	71 to slightly less than 2 million	Random-effect meta-analysis	– Included studies were of moderate to high quality – ADHD was associated with a range of negative life outcomes, including failure to complete high school (OR = 3.70, 1.96–6.99), use of education services (OR = 6.37, 2.58–15.73), dismissal from work (OR = 3.92, 2.68–5.74), substance dependence (OR = 2.45,1.44–4.17), violence – related arrest (OR = 3.63, 2.31–5.70) – Significant heterogeneity (*I* ^2^ = 83.1%) was observed for substance dependence	A better comprehension of the underlying mechanism for the association between ADHD and several long-term outcomes are required to prevent or reduce these long-term adverse outcomes	High (9/9)
Tosto et al. [63]	Determine the relationship between ADHD and mathematics	Mathematical ability, Mathematics achievement	Database inception – February 2015	34	2,000–10,000	Narrative synthesis	– Majority of the included studies were of high quality – There was clear evidence for a negative relationship between ADHD and mathematical ability, which was stronger for inattentive compared to hyperactivity-impulsivity symptoms	More longitudinal research is required to gain a better understanding of the mechanism behind this association	Moderate (6/9)
Di Lorenzo et al. [64]	Present the clinical and social outcomes among adults who suffered from ADHD in childhood or adolescence	Alcohol-related disorders, Antisocial personality disorder, Problem behavior	2015–2020	27	Unclear	Weighted Mean Difference	– Majority of included studies were of high quality – ADHD persisted into adulthood with a mean rate of 43% – ADHD before adulthood was associated with substance or alcohol use disorders and antisocial behavior in adulthood, and, to a lesser degree, with anxiety and depressive disorders	New studies with uniform diagnostic criteria and more studies on nonwestern region are needed	High (9/9)
Charach et al. [65]	Quantify the association between childhood ADHD and future risk of alcohol, nicotine and substance use	Substance use disorder, Alcohol use disorder, Nicotine use	Database inception – October 2009	13	2,000–10,000	Random-effect meta-analysis	– Majority of included studies were of high quality – Children with ADHD were more likely to develop alcohol use disorder in their early adulthood (OR = 1.35, 1.11–1.64) and nicotine use in their middle adolescence (OR = 2.36, 1.71–3.27) compared to controls – However, the magnitude of the association with future psychoactive SUD and nonalcohol drug use disorder was unclear due to the influence of a single study – No heterogeneity were observed across analyses	For better evidence, multiple cohort studies that use survival analysis to assess time to initial substance use outcomes are warranted as it might help to increase power and to determine baseline risk factors	Moderate (6/9)
Fond et al. [66]	Compare the smoking behavior of adult smokers with a *childhood history of ADHD* (CH) to adult smokers without CH	Smoking, Nicotine Dependence	Database inception – 2013	9	365 smokers with CH and 1,708 smokers without	Random-effect meta-analysis	– Included studies were of high quality – Adolescents with CH were found to consume more cigarettes (SMD = 0.15, 0.01–0.28) during adolescence and started smoking at earlier age (SMD: −0.28, −0.49 to −0.07) – However, such associations were not found among adults – Moderate heterogeneity (*I* ^2^ > 60%) were observed across analyses	Additional studies from different countries and in adolescents are needed for better clarification. Research should also investigate the smoking behavior pattern in adulthood	High (9/9)
Oliva et al. [67]	Determine the prevalence of cocaine use and cocaine use disorder among ADHD adults	Cocaine, Cocaine use disorder	Database inception – July 2019	12	3,329	Random-effect meta-analysis	– Majority of the included studies were of high quality – Prevalence of cocaine use was 26.0% (18.0–35.0) and for cocaine use disorder 10.0% (8.0–13.0) – Significant heterogeneity (*I* ^2^ > 95%) were observed across analyses	Studies on effects of stimulants and other treatment for ADHD on cocaine use disorder among ADHD adults are required	High(8/9)
Amiri et al. [68]	Estimate the association between ADHD and injuries	Injuries, Accidents	2000–2014	35	49,200	Random-effect meta-analysis	– Included studies were of moderate quality – Meta-analysis results from all comparative studies showed that individuals with ADHD were nearly two times more likely to be injured (OR = 1.96, 1.63–2.37) – Likewise the odds ratio from cohort studies was (OR = 2.18, 1.46–3.24) – Significant heterogeneity (*I* ^2^ > 95%) were observed across analyses	Individuals with ADHD and caregivers should be informed about the potential risk of injuries, and necessary measures aimed to reduce such risk at the community level should be taken	Moderate (7/9)
Seens et al. [69]	Assess the prevalence of bone fractures among children and adolescents with ADHD	Bone fracture, Injury	Database inception – (date of last search not given)	5	53,849	Random-effect meta-analysis	– Prevalence of bone fractures among children and adolescents with ADHD was 4.83% (3.07–6.58)	Prospective study designs that can follow children and adolescents with ADHD over time to determine the type of fractures, source of injuries and age of fracture are needed	High (8/9)
Ruiz-Goikoetxea et al. [70]	Examine the association between ADHD and risk of unintentional physical injuries in children/adolescents	Unintentional physical injuries, Child Psychiatry	Database inception – June 2017	14	Cases: 371,301 Controls:4,957,511	Random-effect meta-analysis	– Majority of the included studies were of moderate quality – For unintentional injuries, OR was 1.53 (1.40–1.67), while the HR based on the result of four studies was 1.39 (1.06–1.83) – Significant heterogeneity (*I* ^2^ > 82%) were observed across analyses	Further studies should assess the association between different subtypes of ADHD and unintentional injuries	High (9/9)
Cook et al. [71]	Explore the association between ADHD and clinical outcome from sport-related concussion	Brain trauma, Outcome research	Database inception – February 2019	14	Cases: 359 Controls: 3,264	Narrative synthesis	– Methodological weakness of the included studies restricted from making any firm conclusion about the ADHD and the risk of sport related concussion	Longitudinal studies that include a large sample size and adjust for potential confounders are required	Moderate (7/9)
Ruiz-Goikoetxea et al. [72]	Determine the risk of poisoning and compare the magnitude of the risk of unintentional physical injuries and poisoning	Poisoning, Unintentional injuries, Children and adolescents	Database inception – November 2017	35	Cases:84,756 Controls: 1,398,946	Random-effect meta-analysis	– Included studies were of poor quality – Children and adolescents with ADHD have more than three times greater risk of poisoning compared to the control group (RR = 3.14, 2.23–4.42), which was significantly higher than that of unintentional physical injuries (Beta coefficient = 0.68, 0.16–1.20) – Significant heterogeneity (*I* ^2^ > 88%) were observed across analyses	Studies should examine the influence of medication and comorbidities on the risk of poisoning	High(9/9)
Septier et al. [73]	Examine the association between ADHD and SSB	Suicide, Children, Adults	Database inception – April 6, 2018	57	90,805 participants with ADHD and 239,778 without ADHD	Random-effect meta-analysis	– Included studies were of poor quality – An overall significant association between ADHD and SSB was found; suicidal attempt (OR = 2.37,1.64–3.43); suicidal ideations (OR = 3.53, 2.94–4.25); suicidal plans (OR = 4.54, 2.46–8.37), and completed suicide (OR = 6.69, 3.24–17.37) – Significant heterogeneity (*I* ^2^ > 87%) were observed for suicidal attempts and complete suicide, while moderate heterogeneity (*I* ^2^ = 73.73%) for suicidal ideations	Recommend screening for SSB in ADHD patients by clinicians at each visit	Moderate (7/9)
Nourredine et al. [74]	Assess the association between ADHD and the risk of subsequent PD	Childhood ADHD, Psychotic Disorder	Database inception – July 7, 2020	15 (12 in meta-analysis)	1.85 million	Meta-regression using random-effect model	– Majority of the included studies were of high quality – Significant association was found between childhood ADHD and subsequent PD (OR = 4.74, 4.11–5.46) – No significant between-group differences were found for subgroup analyses according to PD (OR = 5.04, 4.36–5.83) or schizophrenia (OR = 4.59, 3.83–5.50) outcomes, cohort (OR = 4.64, 4.04–5.34) or adjusted (OR = 4.72, 4.11–5.46) or unadjusted (OR = 3.81, 1.39–10.49) estimates – No heterogeneity were observed across analyses	Future studies need to determine the mechanisms linking these common conditions	High (9/9)
Baggio et al. [75]	Determine the prevalence of ADHD in detention settings	Prevalence, Prisons	Database inception to January 2, 2018	102	69,997	Meta-regression using random-effect model	– Majority of included studies were of high quality – The prevalence of ADHD among adults was 26.2% (22.7–29.6), and the prevalence of childhood ADHD was 41.1% (34.9–47.2) while measured retrospectively – No significant difference was found between prevalence assessed by screening and those by clinical interviews	Adequate screening, diagnosis, and treatment of ADHD among those living in prisons are warranted	High(8/9)
Mohr-Jensen et al. [76]	Examine the long-term effect of ADHD on criminal outcomes	ADHD, Crime, Longitudinal studies	Database inception – August 2015	11	15,442	Random-effect meta-analysis	– Majority of the included studies were of high quality – Childhood ADHD was associated with a long-term higher risk of convictions (RR = 3.3, 2.1–5.2) followed by incarcerations (RR = 2.9, 1.9–4.3) and arrest (RR = 2.2, 1.3–3.5) – Potential predictors for antisocial outcomes were early behavior problems, childhood maltreatment, sex, and IQ	Prospective studies with a large sample size that clarifies the role of sex, IQ, SES, other mental disorder and childhood maltreatment while assessing the long-term risk of ADHD on criminal activities are needed	High (8/9)
Buitelaar et al. [77]	Summarize the evidence for ADHD and risk of being perpetrator of DV and IPV	Domestic violence, Intimate partner violence	Database inception – January 2015	7	64–11,238 participants per study	Narrative synthesis	Heterogeneity in methodology and outcome measures across studies restricted from making any firm conclusion about the association between ADHD and IPV or DV	Longitudinal studies should include a large sample size and assess the role of potential confounders	High (8/9)
Kittel-Schneider et al. [78]	Analyze current evidence for association between maternal ADHD including stimuland treatment and pregnancy risk, birth outcomes, health behavior in pregnancy, and early parent–child interaction and early child development in the first 3 years	Mother, Father, Pregnancy, Lactation, Breastfeeding, Methylphenidate, Atomoxetine, Guanfacine, Lisdexamfetamine, Amphetamine, Modafinil	Database inception – November 2021	32 (4 to 12 for each of four research questions)	Several different subgroups and methods, including 1,951,940 participants in studies of healthy behavior in pregnancy, 1,266 families or child–parent dyads, and 29,282 exposed and 11,452,476 controls for six medication exposures	Narrative synthesis	– Majority of included studies were of moderate quality – ADHD in girls was associated with teenage and unintended pregnancies, and with pregnancy and birth complications – ADHD in parents was associated with increased parenting stress, risk of ADHD in children, increased aggression in children, and reduced effects of parent training – Maternal ADHD was associated with parenting warmth in some studies – Use of ADHD medications was associated with increased risk for some birth defects, but the increase was low	Further studies with a higher quality are needed to assess the causal mechanism	Moderate (6/9)
Chhibber et al. [79]	Synthesize current evidence on the economic burden of ADHD on the global scale, focusing on describing the methodological variations of studies estimating this burden	Economic burden, High income countries	Database inception – December 2020	44	NA	Narrative synthesis	– Included studies were of high quality – ADHD leads to a substantial economic burden on society – Estimates based on marginal costs ranged from $US244.15 to 18,751.00 for per person estimates and from $US12.18 million to 141.33 billion for national estimates – Studies that calculated economic burden across multiple domains of direct, indirect, and education and justice system costs for both children and adults with ADHD reported higher costs and translated gross domestic product than did studies that captured only a single domain or age group	There is an urgent need to conduct cost-of-illness research in LMICs	High (8/9)

Abbreviations: DV, domestic violence; HR, hazard ratio; IPV, intimate partner violence; *k*, total number of included studies; LMICs, low and middle-income countries; OR, odds ratio; PD, psychotic disorder; RR, relative risk; SSB, suicidal spectrum behaviors.

a Total participants included in the systematic review and meta-analysis unless otherwise indicated.

b For findings from meta-analysis, if given effect estimates with 95% CI are presented unless otherwise indicated.

### ADHD and comorbidities (n = 33, Table 7)

#### ADHD and other mental and neurological disorders

Children and adolescents with ADHD had significantly higher rates of autism spectrum disorder (ASD) (SMD = 1.23, 95% CI: 0.94–1.51) [80] and pragmatic language difficulties than healthy controls [81]. ADHD in adults was strongly related to negative emotionality and low conscientious inhibition [82]. Similarly, the prevalence of bipolar disorder among adults with ADHD was 7.95% (5.31–11.06), and with 4 years earlier age of onset than in bipolar disorder without ADHD [83]. ADHD was associated with eating disorders (OR = 3.82, 2.34–6.24) [84], with similar findings reported in several systematic reviews [85, 86]. Reviews have also shown a link between ADHD and gaming disorder [87] and internet addiction (OR = 3.76, 2.75–5.15) in adolescents and young adults [88].

#### Sleep

While a recent meta-analysis found no significant difference in sleep parameters between individuals with ADHD and healthy controls as measured by polysomnography [89], previous meta-analyses have reported impaired sleep among children with ADHD [90], including those measured by polysomnography [91] or actigraphy [92]. In adults, a meta-analysis showed longer sleep onset latency and lower sleep efficiency among adults with ADHD than without ADHD [93]. Clinically, adults with ADHD had an increased risk of nearly all types of sleep disorders, including insomnia and circadian rhythm disorders [94, 95] and sleep bruxism [96].

#### Obesity

Associations between ADHD and obesity have been found in children, adolescents, and adults [94, 97], with one review reporting the association also after adjustments for possible confounders (children OR = 1.20, 95% CI: 1.05–1.37; adults OR = 1.55, 1.32–1.81) [98]. A review on bariatric surgery showed that patients with ADHD responded equally well as patients without ADHD in terms of change in body mass index after surgery [99].

#### Somatic conditions

Associations with ADHD were reported for headache (OR = 1.98, 95% CI: 1.60–2.45) [100], asthma (OR = 1.52, 1.42–1.63) [101] and (OR = 1.53, 1.41–1.65) [102], eczema (OR = 1.32, 1.20–1.45), and allergic rhinitis (OR = 1.52, 1.43–1.63) [103]. Associations between ADHD and other atopic conditions have also been found [94, 104]. ADHD patients had reduced vagally-mediated heart rate variability (Hedge’s *g* = 0.20, 0.01–0.40) [105], but no associations between adult ADHD and diseases of the circulatory system were found [94].

#### Other associated conditions

Children with ADHD had two times higher risks of having nocturnal enuresis than healthy controls. [106]. Reviews suggested that the findings were inconsistent for the relation between early signs of deviating motor functioning or development and later ADHD [107, 108] and on anxiety and social functioning in children and adolescents with ADHD [109].

ADHD in children was associated with a reduction in global retinal nerve fiber layer thickness (SMD = −0.23, 95% CI: −0.46 to −0.01) [110] and individuals with ADHD showed more oculomotor inhibition failure than control groups [111]. Similarly, altered electrophysiological performance monitoring (i.e., reduced error-related negativity and the error positivity amplitude) during cognitive tasks, indicative of difficulties in evaluating errors in performance, have been reported both in children and adults with ADHD [112].

**Table 7.** ADHD and comorbidities

References	Objective	Keywords	Timeframe of database search	*k*	Sample size^a^	Analytical design	Main findings (Effect estimates, 95% CI)^b^	Conclusion and comments from the authors	Quality assessment
Holingdale et al. [80]	Estimate the prevalence of ASD among children and adolescents with ADHD	Autism Spectrum Disorder, Comorbidity	2007–2017	28	61,985	Random-effect meta-analysis	– Majority of the included studies were of moderate quality −21% of children and adolescents with ADHD also fulfill criteria for ASD – Children with ADHD also had more dimensionally measured ASD traits compared to controls. – There were no significant differences between community and clinical samples or the US and non-US studies – Significant heterogeneity (*I* ^2^ > 87%) were observed across analyses	Future studies should include a large sample size and standard methods. Research assessing the effect of medication on ADHD should also consider the presence of ASD among ADHD patients	High (8/9)
Carruthers et al. [81]	Compare the pragmatic language profiles of children with ADHD to those of TD children and those with autism	Communication, Pragmatic language	Database inception – October 2019	34	Cases: 1,407; Controls: 1,058 Autism: 38	Narrative Synthesis	– Majority of the included studies were of moderate quality – Children with ADHD were found to have higher rates of pragmatic difficulties than their TD peers with specific difficulties linked to inappropriate initiation, presupposition, social discourse, and narrative coherence – Children with ADHD appear to differ from those with autism in the degree of their pragmatic language impairments	Good quality research that assess the differences in profile of pragmatic language impairments in the children with ADHD and those with autism are required	High (8/9)
Jacobsson et al. [82]	Review the associations between adult ADHD and personality traits, organized within a maladaptive five factor framework	Personality disorder, Personality traits	January 2000 – date of last search not given	13	Cases: 2,023 Controls: 16,835	Random-effect meta-analysis	– Majority of the included studies were of moderate quality – Effect size for Negative Emotionality and Conscientious Inhibition were greater and significant (*d* = 1.11 and −0.89, respectively) – Effect sizes for Agreeable Inhibition and Positive Emotionality were moderate and significant (*d* = −0.39 and −0.43, respectively) – Significant heterogeneity (*I* ^2^ > 84%) were observed across analyses	Further research that more specifically delineate how ADHD can be fit into the personality/psychopathology hierarchy are needed	Moderate (6/9)
Schiweck et al. [83]	Investigate the comorbidity of ADHD and BD in adults	Adult bipolar disorder, Prevalence, Comorbidity	Database inception – October 2020	71	646,766	Random-effect meta-analysis	– Prevalence of BD among ADHD patients was 7.95% (5.31–11.06), with earlier age of onset of BD than for persons without ADHD – There was however significant variation observed across the different diagnostic systems, geographic locations, sample size – Significant heterogeneity (*I* ^2^ > 94%) was observed across analyses	The effect of different versions of the specific diagnostic systems requires further clarification, and clinicians should consider the potential presence of BD among adults with ADHD	High (8/9)
Nazar et al. [84]	Assess the comorbidity of ADHD and ED	Eating disorder, ADHD	Not given	22	>45,753	Random-effect meta-analysis	– ADHD patients were nearly four times more likely to have ED compared to controls (OR = 3.82, 2.34–6.24), with significant variation observed across assessment methods – Clinical assessment yielded significantly higher risk (OR = 5.89, 4.32–8.04) than self-report instrument (OR = 2.23, 1.23–4.03) – Significant heterogeneity (*I* ^2^ > 94%) was observed across analysis	There is a need to address whether ADHD patients with a comorbid ED have a different prognosis, course and treatment response when compared to patients with either disorder alone	Moderate (6/9)
Kaisari et al. [85]	Examine the association between ADHD and DEB	Eating disorders, Disordered eating behavior	Database inception – May 2016	72	15,418	Narrative synthesis	– Majority of the included studies were of moderate quality – Existing literature showed moderate evidence for the positive association between ADHD and DEB, particularly overeating behavior	Further studies should include a large representative sample, use uniform assessment techniques and also control for health characteristics, stimulant medication and ADHD-related comorbidities to clarify the nature of the relationship between ADHD and disordered eating behavior	Moderate (7/9)
Curtin et al. [86]	Summarize the evidence for association between ADHD and ED among youth	Adolescents, Eating Disorders, Eating Pathology	1971–2012	8	ADHD: 2,032 Control: 5,173	Narrative synthesis	– Included studies were of moderate quality – The result from available few primary studies showed a positive association between ADHD and ED	Additional studies are required to confirm this finding	Moderate (7/9)
Dullur et al. [87]	Review the association between ADHD and GD	Gaming disorder, Mental health comorbidity	Not given	29	56,650	Narrative synthesis	– Included studies were of moderate quality – ADHD symptoms were consistently associated with GD, with more frequent associations displayed with inattention than other ADHD subscales – No conclusive findings were available regarding the type of game on severity of either condition, or on completion of treatment	Future studies needs to elucidate the direction of the relationship between ADHD and GD	Moderate (6/9)
Wang et al. [88]	Determine the association between IA and ADHD	Internet addiction, ADHD	Database inception – June 2016	15	12,774	Random-effect meta-analysis	– Quality of included studies ranged from poor to moderate – Those with IA were 3.76 times more likely to be diagnosed with ADHD and had more severe symptoms of ADHD compared to those without IA – Moderate heterogeneity (*I* ^2^ > 60%) was observed across analysis	The causal relation between IA and ADHD needs to be studied	High (9/9)
Keenan et al. [89]	Explore potential overlaps and distinctions in objective measures of sleep between PTD and ADHD	Objective sleep measures, Polysomnography	Database inception – September 2020	ADHD only (*N* = 16) ADHD +PTD (*N* = 3)	ADHD-only (*N* = 316) PTD + ADHD (*N* = 79)	Random-effect meta-analysis	– Majority of the included studies were of high quality – Compared to controls, no significant effect was found for. Sleep efficiency, sleep onset latency in ADHD-only populations – A small effect size was found in PTD + ADHD populations with significantly lower sleep efficiency compared to controls (SMD = −0.25,−0.46 to −0.04) and increased sleep onset latency (SMD = 0.33, 0.01–0.66) – Moderate heterogeneity (*I* ^2^ > 40%) was observed across analyses for ADHD only	Greater emphasis should be placed on confounding variables including treatment, symptom severity, cooccurring disorders, and age. In particular, more highly controlled studies with unmedicated ADHD populations are warranted	Moderate (7/9)
Lee et al. [90]	Assess the association between short or long sleep duration and the risk for ADHD	Short sleep duration, Long sleep duration	1971–2019	10	5,963	Random-effect meta-analysis	– Majority of the included studies were of high quality – Short sleep duration was associated with ADHD and particularly with hyperactivity (OR/RR = 1.60,1.18–2.17) – Significant heterogeneity (*I* ^2^ > 90%) was observed across analysis	Prospective studies with adequate power that control for potential confounders are needed to clarify the association	High (8/9)
Diaz-Roman et al. [91]	Examine the association between sleep physiology and ADHD in some sleep parameters	Sleep, ADHD	1987–March 2014	11	500–2,000	Fixed-effect meta-analysis	– Included studies were of high quality – By using polysomnography, it was found that children with ADHD had a lighter sleep than those without ADHD (SMD = 0.32, 0.08–0.55) – No heterogeneity was observed across analysis	Additional studies are needed to confirm this finding	High (8/9)
DeCrescenzo et al. [92]	Determine the role of actigraphy in monitoring of changes in motor activity and in sleep patterns in ADHD	Actigraphy, Sleep	Database inception – July 2014	24	2,179	Random-effect meta-analysis	– Included studies were of high quality – Although sleep duration did not differ, children with ADHD had moderate mean motor activity (SMD = 0.65, 0.45–0.84), higher sleep latency (SMD = 0.51, 0.10–0.92) and lower sleep efficiency (SMD = −0.69,−1.32 to −0.05) compared to typically developing controls – No heterogeneity was observed across analysis	Actigraphy may be a clinically useful tool to monitor motor activity and sleep patterns in children with ADHD, particularly to monitor the effect of medical treatment	High (9/9)
Diaz-Roman et al. [93]	Understand the sleep parameters among adults with ADHD	Sleep, Adults, Actigraphy	Database inception – August 2017	13	Cases: 652 Controls: 769	Random-effect meta-analysis	– Included studies were of high quality – Adults with ADHD had longer sleep onset latency (SMD = 0.67, 0.41–0.92), lower sleep efficiency (SMD = −0.55, −0.83 to −0.27), and higher daytime sleepiness (SMD = 0.75, 0.29–1.21) – Actigraphic measures also showed similar results – Significant heterogeneity (*I* ^2^ = 86%) was observed for daytime sleepiness, while moderate (*I* ^2^ = 54%) for sleep onset latency	Further studies should assess the impact of ADHD subtype and comorbid psychiatric disorders	High (8/9)
Instanes et al. [94]	Review evidence about ADHD and several somatic condition	Obesity, Asthma, Migraine	1994–2015	126	>17,007,576	Narrative synthesis	– Majority of the included studies were of moderate quality – Evidence for an association between adult ADHD and somatic conditions like obesity, sleep disorders, and asthma was found, while preliminary evidence existed for migraine and celiac disease – No association was found for ADHD and cardiovascular disease	Registry-based studies will help to provide better insight into the association by adjusting for familial and unfamilial confounders. Future studies should also use uniform diagnostic assessment	Moderate (7/9)
Lugo et al. [95]	Review sleep disturbances in adults with ADHD	Adults, Sleep	1994–February 2019	39	3,382	Narrative synthesis and random-effect meta-analysis	– Included studies were of high quality – Sleeping problems like large sleep onset latency, poor sleep efficiency, great number of night awakenings, and decreased sleep quality were found more often in ADHD adults than in controls – Significant heterogeneity (*I* ^2^ > 80%) was observed across analysis	Additional studies with a large sample size and with adequate controlling for other covariates like medication and comorbid mental disorder are needed	Moderate (7/9)
Souto-Souza et al. [96]	Examine the relationship between ADHD and bruxism	Bruxism, ADHD	Database inception – April 2019	32	ADHD: 2,629 Bruxism: 1,739	Random-effect meta-analysis	– Majority of the included studies were of moderate quality – Children and adolescents with ADHD were nearly three times more likely to have both sleep bruxism (OR = 2.77, 1.90–4.03), and awake bruxism was 10.64 (2.41–47.03) – Moderate heterogeneity (*I* ^2^ > 60%) was observed across analysis	High quality studies with robust methodology are warranted	High (8/9)
Li et al. [97]	Estimate the global prevalence of obesity, overweight and underweight among ADHD patients	ADHD, Obesity, Prevalence	Database inception – June 2020	48	Not given	Random-effect meta-analysis	– Majority of the included studies were of moderate quality – A high prevalence of overweight (20.9% (18.5–23.3)) and obesity (14.7% (12.9–16.4)) was found among individuals with ADHD	Well-designed studies assessing causality between ADHD and obesity and overweight are required	High (9/9)
Cortese et al. [98]	Examine the association between obesity/overweight and ADHD	Obesity, Overweight, ADHD	Databases inception – August 2015	42	Cases: 48,161 Controls: 679,975	Random-effect meta-analysis	– Majority of the included studies were of moderate quality – A significant association exists between ADHD and obesity among children/adolescents and adults with ADHD – Moderate heterogeneity (*I* ^2^ > 50%) was observed across analyses	The underlying mechanism for the relationship between ADHD and obesity needs to be established	High (9/9)
Mocanu et al. [99]	Assess the impact of ADHD on outcomes of bariatric surgery	Bariatric surgery, Obesity, ADHD	1946–August 2018	5	492	Random-effect meta-analysis	– Included studies were of moderate quality – Patients with ADHD did not have a statistically significant mean BMI difference compared to patients without ADHD following bariatric surgery – However, they had a statistically significant reduction in postoperative follow-up time (months) versus non-ADHD patients (MD = −7.28, −13.83 to −0.73). – Significant heterogeneity (*I* ^2^ = 84%) was observed for mean BMI change	Studies that elucidate the neurophysiologic mechanisms of obesity and their impact on associated mental health disorders are needed	Moderate (6/9)
Pan et al. [100]	Investigated the cooccurrence of headache in children with ADHD	Headache, Comorbidity	Database inception – June 2020	13	Cases: 267,556 Controls: 2,464,878	Random-effect meta-analysis	– Majority of the included studies were of high quality – The pooled prevalence of headaches in children with ADHD was 26.6% – Significant heterogeneity (*I* ^2^ > 93%) was observed across analyses	For example, no eligible longitudinal studies aiming to disentangle the temporal relationship between the emerging of attention problems and headache symptoms were identified	High (8/9)
Kaas et al. [101]	Investigate the association between asthma and ADHD	ADHD, asthma	Database inception – March 2019	25	Not given	Random-effect meta-analysis	– Majority of the included studies were of moderate quality – Significant association was found between asthma and ADHD (OR = 1.52, 1.42–1.63) – Moderate heterogeneity (*I* ^2^ = 60%) was observed	Clinicians should be aware of such association to aid an early diagnosis and treatment of such comorbidity	Moderate (6/9)
Cortese et al. [102]	Assess the association between ADHD and asthma	ADHD, asthma	Not given	84	Cases: 210,363 Controls: 3,115,168	Random-effect meta-analysis	– Included studies were of high quality – Significant association found between asthma and ADHD (unadjusted OR = 1.66, 1.22–2.26), and (adjusted OR = 1.53, 1.41–1.65) – Significant heterogeneity (*I* ^2^ = 99.47%) was observed for unadjusted analysis, while moderate heterogeneity (*I* ^2^ = 50.76%) was observed for adjusted analysis	Further cohort studies are required to examine the causal relationship between ADHD and asthma	High (8/9)
Schans et al. [103]	Investigate the association between atopic disorders and ADHD in children and adolescents	Asthma, atopic eczema, allergic rhinitis	Database inception – September 2015	28	166,375	Narrative synthesis and random-effect meta-analysis	– Majority of the included studies were of moderate quality – ADHD was significantly associated with atopic disorders like asthma (OR = 1.34, 1.24–1.44), atopic eczema (OR = 1.32, 1.20–1.45), and allergic rhinitis (OR = 1.52,1.43–1.63) – Significant heterogeneity (*I* ^2^ = 82%) was observed for allergic rhinitis, while for other analysis no heterogeneity was observed	Cohort studies that perform multiple measurements for atopic disorder symptoms among ADHD individuals are warranted to clarify the association	High (8/9)
Miyazaki et al. [104]	Examine the association between ADHD and allergic diseases in children	Allergic diseases, ADHD	Database inception – November 2015	5	61,811	Random-effect meta-analysis	– Majority of the included studies were of moderate quality – Children with ADHD were nearly two times more likely to have asthma (OR = 1.80, 1.57–2.07) than control – Significant associations were found between ADHD and allergic rhinitis (OR = 1.59, 1.13–2.32), atopic dermatitis (OR = 1.43, 1.09–1.88), and allergic conjunctivitis (OR = 1.69, 1.04–2.76) compared to controls – No significant difference was found between ADHD and control group for food allergies (OR = 1.13, 0.88–1.47) – Significant heterogeneity (*I* ^2^ > 86%) was observed for allergic rhinitis, atopic dermatitis, allergic conjunctivitis, while moderate heterogeneity (*I* ^2^ = 60%)was observed for asthma analysis	Longitudinal studies that adjust for potential confounders are needed	High (9/9)
Robe et al. [105]	Estimate the effect of ADHD on HRV	Heart rate variability, Vagally-mediated HRV	Database inception – January 2018	13	Cases:856 Controls: 905	Random-effect meta-analysis	– Majority of the included studies were of high quality – Low vagally-mediated HRV was noted among individuals with ADHD (Hedge’s *g* = 0.20, 0.01–0.40) compared to healthy subjects – Moderate heterogeneity (*I* ^2^ = 77%)was observed across analysis	Additional studies to further clarify this association are required	High (8/9)
Oliveira et al. [106]	Assess the cooccurrence of ADHD and nocturnal enuresis	Nocturnal enuresis, Enuresis	Database inception – June 2020	25 (13 in meta-analysis)	Cases: 16,000 Controls: 59,600	Random-effect meta-analysis	– Included studies were of moderate quality – ADHD children had more than two times higher risk of having enuresis than controls (OR = 2.49, 2.12–2.93) – Children with enuresis had two times higher risk of having ADHD than those without enuresis (OR = 2.03, 1.20–3.42) – Moderate heterogeneity (*I* ^2^ = 75%)was observed for the association between enuresis and ADHD	Additional studies with larger sample and with uniform terminology, are required	Moderate (6/9)
Athanasiadou et al. [107]	Investigate early motor signs of ADHD	Early motor signs, Infancy, ADHD	Database inception – January 2017	9	7,383	Narrative synthesis	– Included studies were of high quality – Small sample size and studies focusing on group reports rather than individuals limited power to show association between early motor signs and later ADHD	Cohort studies with large sample size and that use more precise measurements are required	High (8/9)
Havmoeller et al. [108]	Assess relationship between early motor development before 3 years of age in children and later ADHD	ADHD, Early motor development	Database inception – February 2018	5	65,016	Narrative synthesis	– Included studies were of high quality – Variation in results from individual studies restricted from drawing any firm conclusion about early motor development and ADHD	Cohort studies with a large population and clinically diagnosed children, that consider clinical features of ADHD and other potential covariates like sex, comorbid mental disorder need to be conducted	Moderate (7/9)
Bishop et al. [109]	Assess the association between anxiety and social functioning (social problems, peer status, and social skills/ competence) in children and adolescents with ADHD	ADHD, Anxiety, Social functioning	Database inception – August 2018	31	5,116	Narrative synthesis	– Included studies were of high quality – The association between anxiety and social functioning among individuals with ADHD varied substantially across individual studies	Future research that controls for potential confounders are needed to elucidate the association between anxiety and social functioning among individuals with ADHD	Moderate (6/9)
Li et al. [110]	Examine the relationship between ADHD and associated neuro retinal features (i.e., RNFL thickness and GCL thickness)	Ocular biomarker, Retinal Nerve Fiber Layer, Ganglion Cell Layers	Unclear	4	Cases:164 Controls: 150	Random-effect meta-analysis	– Included studies were of moderate quality – Meta-analysis revealed that ADHD in children was associated with a reduction in global RNFL thickness (SMD = − 0.23, −0.46 to −0.01) – The global GCL thickness between ADHD children and controls was not statistically significant – Significant heterogeneity (*I* ^2^ > 75%) were observed across analysis	Further larger-scale, multi-ethnic, and longitudinal studies are needed to confirm this association	Moderate (7/9)
Chamorro et al. [111]	Assess the effects reported in studies comparing oculomotor inhibition in ADHD patients and healthy control subjects	Biological markers, Oculomotor inhibition	Not given	31	Cases: 766 Controls: 801	Random-effect meta-analysis	– Majority of included studies were of moderate quality – Among inhibitory outcomes, direction errors in AS, showed a moderate effect (*g* = 0.57, 0.27–0.88), while anticipatory saccades in memory-guided saccade and saccades during prolonged fixation showed a large effect (*g* = 0.86, 0.64–1.08) and (*g* = 1.11, 0.56–1.65) respectively – Significant heterogeneity (*I* ^2^ > 74%) were observed across analysis	Further research to integrate findings across different pathologies would help to identify what mechanisms are specific for each condition	Moderate (7/9)
Bellato et al. [112]	Investigate electrophysiological correlates of performance monitoring (error-related negativity, ERN; error positivity, Pe; feedback-related negativity, FRN; feedback-P3) in ADHD individuals	Electroencephalography, Electrophysiological correlates	Database inception – February 2021	46 (28 in meta-analysis)	Cases: 1,050 Controls: 1,076	Random-effect meta-analysis	– Majority of the included studies were of poor quality – ERN and Pe amplitude were significantly reduced in ADHD compared to controls (*g* = −0.47, −0.67 to −0.26) and (*g* = −0.50, −0.69 to −0.32) respectively	Larger samples, collecting, and processing EEG data under conditions suitable for data pooling across studies will help to systematically investigate the cross-study heterogeneity	High (9/9)

Abbreviations: ASD, autism spectrum disorder; BD, bipolar disorder; BMI, body mass index; DEB, disorder eating behavior; ED, eating disorder; ERN, error-related negativity; FRN, feedback-related negativity; GCL, ganglion cell layers; GD, gaming disorder; HRV, heart rate variability; IA, internet addiction; *k*, total number of included studies; OR, odds ratio; PTD, persistent tic disorder; RNFL, retinal nerve fiber layer thickness; RR, relative risk; SMD, standardized mean difference; TD, typically developing.

a Total participants included in the systematic review and meta-analysis unless otherwise indicated.

b For findings from meta-analysis, if given effect estimates with 95% CI are presented unless otherwise indicated.

### Pharmacological treatment (n = 65, Table 8)

#### Efficacy, acceptability, and tolerability of ADHD medication

##### Children and adolescents

Systematic reviews have shown efficacy for pharmacological treatment of ADHD, with SMDs between 0.6 and 0.8 and consistently stronger efficacy for stimulants than nonstimulants [113, 114]. All-cause treatment discontinuation was lower with pharmacological treatment than placebo (OR = 0.68, 95% CI: 0.58–0.79) [114].

##### Stimulants

In terms of efficacy, acceptability, and tolerability, evidence exists for methylphenidate (MPH) as the preferred first choice for pharmacological treatment of ADHD [115–120]. For instance, in a network meta-analysis of 82 randomized controlled trials (RCTs) including more than 14,000 children and adolescents, at time point closest to 12 weeks, with clinician’s rating, MPH was found to be efficacious in reducing ADHD core symptoms as compared to placebo (SMD = –0.78,–0.93 to −0.62) [115]. MPH was the only drug that had better acceptability than placebo, and with similar tolerability as placebo [115]. A systematic review of four prospective or naturalistic studies and three RCTs showed MPH immediate-release (MPH-IR) as efficacious also for periods longer than 12 weeks (parent ratings for inattention and hyperactivity/impulsivity: SMD = 0.96, 0.60–1.32 and 1.12, 0.85–1.39, respectively) [116].

Lisdexamfetamine (LDX) was also efficacious in reducing ADHD symptoms compared to placebo [121, 122], for example, as reported in a meta-analysis of 28 double-blind, placebo-controlled RCTs with around 4,700 participants (SMD = –1.28, −1.84 to −0.71) [121].

However, according to the earlier Cochrane review and meta-analyses, the efficacy of stimulants like MPH [123] and amphetamine [124] cannot be unequivocally established, due to methodological limitations. The authors point to small number of trials, short follow-up time, low-quality data, high risk of bias in several domains, including lack of sufficient blinding and selective outcome reporting, and heterogeneity between studies. This quite divergent conclusion is probably not due to different evidence included in the systematic reviews, but subjectively different interpretation of the evidence by the authors of different reviews.

##### Nonstimulants

Guanfacine was reported as safe and efficacious in treating ADHD compared to placebo, pooled (OR = 3.18, 95% CI: 2.44–4.13) [125]. Compared to placebo, atomoxetine (ATX) was also reported as safe and efficacious in reducing ADHD symptoms. [121, 126]. A meta-analysis from 1999 also suggested similar findings for clonidine [127]. However, as for stimulants, meta-analyses and Cochrane review stated that the efficacy of some of the nonstimulants cannot be established due to short follow-up time, low quality, limited number of studies, sample sizes, and heterogeneity [128–130]. There is thus disagreement on how to interpret the literature also for nonstimulants.

#### Adults

##### Stimulants

A network meta-analysis of 52 RCTs, including more than 10,000 adults, supported amphetamines as the preferred first pharmacological choice for treatment of ADHD both regarding efficacy (clinicians’ ratings, SMD = –0.79, 95% CI: −0.99 to −0.58) and tolerability (OR = 3.26, 1.54–6.92) [115]. Other reviews have also shown the efficacy of LDX, with one meta-analysis of 19 RCTs with more than 5,500 participants, reporting an SMD of −0.89 (−1.09 to −0.70) [131] and another suggesting an SMD of −0.97 (−1.15 to −0.78) [132] for short-term treatment, compared to placebo. MPH also showed efficacy in reducing ADHD symptoms as compared to placebo (OR = 2.66, 2.12–3.33), and in terms of treatment discontinuation no significant difference was found between MPH and placebo (OR = 1.19, 0.82–1.74) [133]. An indirect comparison meta-analysis of placebo-controlled trials from 2008 suggested shorter-acting stimulants, specifically IR-MPH as efficacious in reducing ADHD symptoms in adults [134]. There was disagreement on how to interpret the original efficacy studies for adults as well. Cochrane reviews suggested that due to high risk of bias, limited sample sizes and number of studies, and heterogeneity in findings, the quality of the evidence for the efficacy of immediate-release MPH (compared to placebo or lithium) [135] and amphetamine (compared to placebo) [136] was low to very-low.

##### Nonstimulants

In a meta-analysis of 12 RCTs with around 3,400 participants, ATX was found to have small efficacy in reducing the severity of ADHD symptoms (SMD = –0.33, 95% CI: −0.43 to −0.23) and increased rates of discontinuation compared to placebo (OR = 1.39, 1.17–1.64) [137]. Cochrane review found low-quality evidence to conclude about the efficacy of bupropion in reducing ADHD symptoms severity and its tolerability compared to placebo [138]. In another review, bupropion was found to be as efficacious as MPH, but due to limited number of studies, the authors suggested the findings as preliminary [128].

#### ADHD pharmacotherapy as a class

A network meta-analysis suggested that, despite a positive class effect of ADHD medication relative to placebo in improving clinical response, the quality of evidence was low to very low about the efficacy of different ADHD drugs on treating ADHD symptoms in adults [139].

#### Adverse effect of ADHD medication

##### Cardiovascular

Patients in all age groups showed significant increases in heart rate and systolic blood pressure (SBP) from pre- to post-treatment when treated with MPH as compared to placebo (for SBP, SMD = 1.61, 95% CI: 0.81–2.41 for children and adolescents and SMD = 1.40, 0.62–2.18 for adults) [140]. Furthermore, children and adolescents treated with ATX had a significant increase in SBP compared to those treated with MPH [140]. However, no association was found between ADHD medication and the number of serious adverse medical events including sudden death, arrhythmia, stroke, myocardial infarction, and all-cause mortality [141].

##### Sleep

Longer sleep latency (effect size = 0.54, 95% CI: 0.28–0.81) and shorter sleep duration (effect size = −0.59, −0.84 to –0.35) were noted in children and adolescents that used stimulants [142]. Similar findings were noted for children treated with MPH as measured by actigraphy for both longer sleep latency and shorter sleep duration [143].

##### Headache

Children and adolescents that used ADHD drugs were at increased risk of having headache during treatment period compared to placebo (for guanfacine, OR = 1.43, 95% CI: 1.12–1.82; for MPH, OR = 1.33, 1.09–1.63) [100].

##### Appetite

Short-term MPH treatment decreased appetite relative to placebo (RR = 3.66, 95% CI: 2.56–5.23) [144]. This was also found for extended-release mixed amphetamine salts and ATX [145].

##### Pregnancy and postpartum-related side effects

No evidence of adverse offspring consequences of ADHD medication during pregnancy was found [78, 146, 147]. MPH was reported as safe to use during breastfeeding, while the reported stimulating effect of guanfacine on prolactin secretion was considered to affect breastfeeding negatively [78].

#### Adverse events of MPH treatment

A meta-analysis showed low quality and reliability of evidence for adverse events of both short and long-term MPH treatment in children and adolescents and young adults [148].

#### Efficacy of medication on ADHD symptoms in patients with comorbid conditions

Seven systematic reviews reported about the efficacy of medication on ADHD symptoms in patients with comorbid disorders [149–156]. For example, both MPH and ATX were efficacious in reducing ADHD symptoms also in youth with ASD [149]. MPH was further found to decrease ADHD symptoms compared to placebo in children and adolescents with borderline intellectual functioning or intellectual disability (ID) (Hedges’ *g* = 0.87, 95% CI: 0.61–1.14) [150]. However, another review stated that the quality of evidence to conclude MPH as efficacious in treating ADHD symptoms in ID as poor [151]. No sufficient evidence was found to conclude about the effect of either amphetamine [157] or risperidone [158] in the treatment of ADHD symptoms in people with ID.

#### Efficacy of medication on comorbidity and other consequences of ADHD

In general, pharmacotherapy for ADHD was found to be efficacious (SMD = 0.40, 95% CI: 0.25–0.55) in reducing both ADHD symptoms and substance use in patients with both ADHD and substance use disorder [159].

ADHD medications generally lowered the risk of injury (rate ratio = 0.76, 0.61–0.93) in individuals with ADHD, based on within-individual analysis [160]. However, a recent meta-analysis revealed higher risk of bone fracture among individuals treated with nonstimulants for ADHD (OR = 1.37, 1.20–1.58) whereas lower, but nonsignificant risk was observed for individuals treated with stimulants [161].

Youths treated with stimulants had lower smoking rates than untreated youths with ADHD (OR = 0.51, 0.32–0.80) [162]. Further, long-term use (>90 days) of stimulants among ADHD individuals was associated with lower risk of suicide attempt (RR = 0.75, 0.66–0.84), based on within-individual analysis [163].

In-school assignment was increased by 15% and on-task behavior by 14% or more among ADHD children treated with stimulants [164]. Likewise, MPH improved instrumental learning in children with ADHD [165]. Compared to placebo adult ADHD patients using MPH scored better on several neurocognitive measures and tests of driving ability (Hedge’s *g* = 0.17, 0.05–0.28) [166].

#### Effect modification of comorbid conditions and drug effects on cooccurring symptoms

Comorbid anxiety did not change the efficacy of ATX on ADHD in children, adolescents, and adults [167]. While ADHD medications like MPH and ATX showed smaller effects, LDX was moderately efficacious in treating emotional dysregulation in adults with ADHD (SMD = –0.50; 95% CI: −0.80–0.21) [168]. Compared to placebo, stimulants were efficacious in reducing both overt and covert aggression in children and adolescents with ADHD [169]. Similarly, risperidone reduced aggressive behavior in youth with ADHD [170].

#### Pharmacogenetics and animal models

While noradrenergic gene polymorphisms were associated with improved MPH response in children with ADHD [171], low-quality evidence exists regarding the impact of SLC6A3 polymorphisms in response to MPH in children with ADHD [172]. No effect was found for MPH on hypertensive rats as an animal model of ADHD [173].

#### Pharmacological treatment rate

The first systematic review on undertreatment, overtreatment, and misuse of ADHD medication reported that 19.1% (95% CI: 11.5–29.9) of school-aged children and adolescents having ADHD were treated with medication for the disorder, and 0.9% (0.5–1.7) of individuals without the diagnosis used medication for ADHD. Their findings indicated both overtreatment and misuse of drugs in individuals without ADHD, and undertreatment of ADHD drugs in youths with the disorder [174].

**Table 8.** Pharmacological treatment

References	Objective	Keywords	Timeframe of database search	*k*	Sample size^a^	Analytical design	Main findings (Effect estimates, 95% CI)^b^	Conclusion and comments from the authors	Quality assessment
**Efficacy, acceptability, and tolerability in children and adolescents**									
**Children and adolescents**									
**Stimulants**									
Cerrillo-Urbina et al. [113]	Examine the efficacy and safety of stimulant and nonstimulant medication	Children and adolescents, Stimulants, Nonstimulant	Database inception – August 31, 2017	15	4,648	Random-effect meta-analysis	Included studies were of high quality Stimulant medications showed greater efficacy (SMD = −0.83,−1.11 to −0.54) than nonstimulants (SMD = −0.58,−0.69 to −0.46) in reducing ADHD-RS-IV score Similar results were found on inattention and hyperactivity-impulsivity subtypes The most frequent adverse effects for stimulants and nonstimulant, respectively, were decreased appetite and somnolence Significant heterogeneity (*I* ^2^ > 80%) was observed across analysis except for nonstimulants	Future research should assess the long-term efficacy of these medications and consider the effect of dosage, frequency, use of different outcome measurement	High (9/9)
Riera et al. [114]	Assess all-cause treatment discontinuation and efficacy of pharmacological treatment	Efficacy, Discontinuation, Children and adolescents	Not given	63	11,788	Random-effect meta-analysis and meta-regression	Included studies were of moderate quality Patients receiving pharmacological treatment were less likely to discontinue treatment than those receiving placebo (OR = 0.68, 0.58–0.79) Pharmacological treatment showed great to moderate efficacy in reducing ADHD symptom severity irrespective of raters; Clinician SMD = 0.74 (0.65–0.84), teacher SMD = 0.75 (0.64–0.86), parent SMD = 0.63 (0.54–0.72) Studies sponsored by industries showed greater clinician-rated efficacy Moderate heterogeneity (*I* ^2^ > 50%) was observed for discontinuation and efficacy analysis	Effects of several covariates like age, gender, ADHD subtype, comorbidity, types of drug, dosage, frequency, nonpharmacological interventions, and sponsorship should be considered by future multi-treatment meta-analysis and RCTs	High (8/9)
Cortese et al. [115]	Compare efficacy and tolerability of oral medications for ADHD	Oral medication, ADHD, Children and adolescents	1987–2017	133 of which 82 in children and adolescents	14,346	Network meta-analysis	Majority of the included studies were of moderate quality For ADHD core symptoms rated by clinicians closest to 12 weeks, all included drugs showed greater efficacy than placebo (SMD = −1.02,−1.19 to −0.85) for AMP, (SMD = −0.78, −0.93 to −0.62) for MPH, (SMD = −0.56, −0.66 to −0.45) for ATX) However, for available comparisons based on teachers ratings, only MPH (SMD = −0.82, −1.16 to −0.48) and modafinil (SMD = −0.76, −1.15 to −0.37) showed greater efficacy than placebo In terms of tolerability,AMP (OR = 2.30, 1.36–3.89) and gunfacine (OR = 2.64,1.20–5.81) showed smaller tolerability than placebo	Further, research on the long-term effects of medications is urgently needed	High (8/9)
Maia et al. [116]	Determine the long-term effects (>12 weeks) of MPH-IR	Children and adolescents, Methylphenidate immediate release	Database inception – April 2014	7	500–2,000	Random-effect meta-analysis and meta-regression	Included studies were of moderate quality MPH-IR showed greater efficacy than placebo regardless of ADHD subtype and raters SMD for inattention using parents rating was 0.96 (0.60–1.32), and with teacher rating 0.98 (0.09–1.86) Similarly, SMD for hyperactivity/impulsivity using parents rating was 1.12 (0.85–1.39), and for teacher rating 1.25 (0.70–1.81) Significant heterogeneity (*I* ^2^ > 80%) was observed for inattention, while moderate heterogeneity was observed for hyperactivity/impulsivity (*I* ^2^ > 64%)	Additional comparative meta-analyses should be performed to determine the pooled head-to-head long-term efficacy of available ADHD medications	Moderate (7/9)
Rezaei et al. [117]	Compare the efficacy of ATX and MPH	Children and adolescents, Atomoxetine, Methylphenidate	Database inception – 2015	11	2,772	Random-effect meta-analysis	Included studies were of moderate quality No significant difference was found in efficacy between immediate-release MPH and ATX However, in subgroup analysis OROS MPH was found moderately efficacious than ATX (SMD = 0.31, 0.16–0.47) in reducing ADHD symptoms Significant heterogeneity (*I* ^2^ > 63%) was found across analysis	Studies with long-term follow-up are needed	Moderate (7/9)
Liu et al. [118]	Compare the efficacy and safety of atomoxetine (ATX) and methylphenidate (MPH)	Children and adolescents, ATX, MPH	Database inception – April 2016	11	3,317	Random-effect meta-analysis	Included studies were of high quality MPH showed increase response rate (RR = 1.14, 1.09–1.20), reduced inattention symptoms (SMD = −0.13, −0.25 to −0.01), and decreased risk of drowsiness (RR = 0.17, 0.11–0.26) nausea (RR = 0.49, 0.29–0.85) and vomiting (RR = 0.41, 0.27–0.63), than ATX However, ADHD patients taking MPH have more than twice the risk of insomnia, than those taking ATX (RR = 2.27, 1.63–3.15) No heterogeneity was observed across analysis	Additional clinical trials with a large sample size and long-term follow-up are required	Moderate (7/9)
Maneton et al. [119]	Examine the efficacy, acceptability, and tolerability of d-MPH in children and adolescents with ADHD	Children and adolescents, dex-methylphediate	January 2002–February 2015	12	1,124	Random-effect meta-analysis	Included studies were of high quality Greater efficacy of d-MPH than placebo (SMD = −1.20, −1.73 to −0.67) was found There was no significant difference in overall discontinuation and discontinuation due to adverse events between the d-MPH and placebo group Moderate to significant heterogeneity was found across analysis	Suggest caution in interpreting the findings due to significant heterogeneity, industry sponsor studies, variation in study settings and recommends further study to address these limitations	High (9/9)
Punja et al. [120]	Summarize the efficacy and safety of long-acting versus short-acting MPH	Children and adolescents, MPH	Database inception – 2012	13	882	Random-effect meta-analysis	Majority of the included studies were of poor quality Long-acting MPH was found to have small but significant efficacy in reducing hyperactivity/impulsivity symptoms on parents ratings(SMD = −0.30, −0.51 to −0.08), while short-acting MPH had small but significant efficacy in reducing hyperactivity symptoms using teacher ratings (SMD = 0.29, 0.05–0.52) Significant high heterogeneity (*I*^2^ > 83% ) was found across analysis	Apart from assessing risk to benefit ratio, future clinical trials should also assess the cost-to-benefit ratio of these medications and perform analysis according to ADHD subtype	Moderate (7/9)
Stuhec et al. [121]	Compare the efficacy and acceptability of bupropion, ATX, LDX, and MPH	Children and adolescents, Bupropion, Atomoxetine, Lisdexamfetamine, MPH	Database inception – April 2014	28	4,699	Random-effect meta-analysis	Majority of the included studies were of poor quality LDX was found to have greater efficacy (SMD = −1.28,−1.84 to −0.71), followed by MPH (SMD = −0.75,−0.98 to −0.52) and ATX (SMD = −0.68, −0.76 to 0.59), and bupropion (SMD = 0.32, 0.69–0.05), compared to placebo Patients receiving MPH were 0.35 times less likely to discontinue medication than those receiving placebo (OR = 0.35, 0.24–0.52) However, no significant difference was observed for treatment discontinuation between ATX, LDX, bupropion, and placebo Significant heterogeneity (*I* ^2^ = 80%) was observed for the efficacy of MPH analysis, while moderate heterogeneity (*I* ^2^ > 60%) was observed for LDX and ATX	Research using high-quality methodology is required to assess the efficacy of bupropion	High (8/9)
Manetton et al. [122]	Assess the efficacy, acceptability, and tolerability of LDX	Children and adolescents, Lisdexamfetamine	January 2007–September 2014	5	1,016	Random-effect meta-analysis	Majority of the included studies were of moderate quality Compared to placebo, LDX showed greater efficacy in reducing ADHD symptom severity (WMD = −15.20, −19.95 to −10.46) No significant difference was observed for overall discontinuation rate and discontinuation due to adverse events between LDX and placebo group No heterogeneity was observed across the analysis	The small number of evidence in primary studies limits the relevance of findings from this review, and hence, further clinical trials are needed to provide conclusive evidence	High (9/9)
Storebø et al. [123]	Assess the effect of MPH in treating ADHD	Children and adolescents, Methylphenidate	Database inception – March 2015	185	5,111	Random-effect meta-analysis	The presence of high risk of bias in all included trials and methodological, clinical, and statistical heterogeneity restricted from making any firm conclusion about the beneficial effect of MPH However, MPH was found to cause nonserious adverse events like loss of appetite, sleeping problems	Recommends conducting “nocebo trials” as MPH was found to be associated with serious adverse events making it difficult for blinding of the participants and outcome assessors	High (9/9)
Punja et al. [124]	Review the evidence for efficacy and safety of amphetamines	Children and adolescents, Amphetamines	Database inception – August 2015	23	2,675	Random-effect meta-analysis	The presence of a high risk of bias in most of the studies, low to very low quality of evidence restricted from making any firm conclusion about the efficacy and safety of amphetamine	Studies that assess the long-term effect of amphetamines and perform subgroup analysis for important factors like age, gender, ADHD subtype, dose, and frequency are needed	High (8/9)
**Nonstimulants**									
Ruggiero et al. [125]	Examine the efficacy and safety of guanfacine	Children and adolescents, Guanfacine	Database inception – May 2014	7	1,752	Random-effect meta-analysis, Sensitivity analysis using fixed-effect model	Majority of the included studies were of moderate quality Compared to placebo guanfacine was found to be efficacious in treating ADHD symptoms (OR = 3.2, 2.4–4.1) Somnolence, headache, and fatigue were the most common adverse event associated with the use of guanfacine Moderate heterogeneity (*I* ^2^ = 74%) was observed for analysis of treatment effect	Head-to-head trials are needed to identify the efficacy of guanfacine compared to other ADHD medications. Such trials should consider the efficacy of medication against comorbidities for ADHD	Moderate (7/9)
Cheng et al. [126]	Assess the efficacy and safety of atomoxetine	Atomoxetine, Efficacy, Children and adolescents	January 1985–September 2006	9	1,828	Random-effect meta-analysis	Majority of the included studies were of moderate quality ATX was efficacious in reducing ADHD symptoms (SMD = −0.64,−0.75 to −0.53) No significant heterogeneity was observed across the analysis	ATX is efficacious in reducing ADHD symptoms	High (8/9)
Connor et al. [127]	Assess the efficacy of clonidine	Clonidine, Children and adolescents	Database inception – 1999	11	209	Random-effect meta-analysis	Majority of the included studies were of moderate quality Clonidine showed moderate efficacy (Hedge’s *g* = 0.58 ±0.16, 0.27–0.89) on symptoms of ADHD in children and adolescents with ADHD and ADHD cornorbid with conduct disorder, developmental delay, and tic disorders	Further high-quality randomized controlled trial are needed to understand the efficacy and safety of clonidine in reducing ADHD symptoms and comorbid conditions	Moderate (7/9)
Manetton et al. [128]	Compare efficacy, acceptability, and tolerability of bupropion and MPH	Children and adolescents, Bupropion, Methylphenidate	Database inception – January 2014	2	62	Random-effect meta-analysis	Insufficient data from the small number of studies and the small sample size restricted from making any firm conclusion about the possible differences in efficacy, acceptability, and tolerability between these medications	Further clinical trials should be conducted to provide firm conclusion	High (8/9)
Otasowie et al. [129]	Evaluate the efficacy of Tricyclic antidepressant	Children and adolescents, Tricyclic antidepressant	Database inception – September 2013	6	216	Random-effect meta-analysis	Less number of trials, small sample size, and heterogeneity in outcome measures restricted from making any firm conclusion about the efficacy of desipramine	Longitudinal multicenter observational studies are needed as their result can also be generalized to clinical population	High (9/9)
Matsui et al. [130]	Summarize the beneficial effect of azapirones (buspirone) compared to other medications	Children and adolescents	Database inception – October 2015	8	499	Narrative synthesis and random-effect meta-analysis	Limited data restricted from making any firm conclusion about the efficacy of buspirone in the treatment of ADHD	Studies assessing the long-term efficacy and safety of azapirone, including larger sample, are needed	High (8/9)
**Adults**									
**Stimulants**									
Cortese et al. [115]	Compare efficacy and tolerability of oral medications for ADHD	Oral medication, Adult	1987–2017	52	10,296	Network meta-analysis	Majority of included studies were of moderate quality For ADHD core symptoms rated by clinicians, closest to 12 weeks, AMP (SMD = −0.79, −0.99 to −0.58), MPH (SMD = −0.49, −0.64 to −0.35), and ATX (SMD = −0.45, −0.58 to −0.32), showed greater efficacy than placebo AMP had greater tolerability (OR = 3.26,1.54–6.92), and ATX (OR = 2.33, 1.28–4.25), MPH (OR = 2.39; 1.40–4.08), and modafinil (OR = 4.01; 1.42–11.33) were less well tolerated than placebo in adults	Future network meta-analysis should include individual patient data for a more reliable estimation of predictors of individual response. Further, research on the long-term effects of medications is urgently needed	High (8/9)
Stuhec et al. [131]	Assess the efficacy, acceptability, and tolerability of lisdexamfetamine, mixed amphetamine salts, modafinil, and methylphenidate with placebo in adults	ADHD, Adults, Psychostimulants	Database inception – May 2016	20 (19 in quantitative synthesis)	5,528	Random-effect meta-analysis	Majority of the included studies were of high quality LDX showed greater efficacy (SMD = −0.89,−1.09 to −0.70), while moderate efficacy was shown for amphetamine salts (SMD = −0.64,−0.83 to −0.45) and MPH (SMD = −0.50, −0.58 to −0.41), in reducing ADHD symptoms compared to placebo No significant effect was found for modafinil No heterogeneity was observed across the analysis	Meta-analyses of head-to-head trials of stimulants and nonstimulants are required. Additional clinical trials are needed to assess the efficacy of modafinil	High (9/9)
Maneeton et al. [132]	Evaluate the efficacy, acceptability, and tolerability of lisdexamfetamine in adults	Lisdexamfetamine, Placebo	Database inception – January 2014	5	822	Random-effect meta-analysis	Included studies were of moderate quality LDX showed greater efficacy (SMD = −0.97, −1.15 to −0.78) in reducing severity of ADHD symptoms compared to placebo Overall response rate was significantly greater for LDX than placebo (RR = 1.99, 1.50–2.63) No significant difference was found for overall discontinuation rate and adverse event-induced discontinuation No heterogeneity was observed across the analysis	Further clinical trials on LDX are warranted	High (9/9)
Castells et al. [133]	Compare all-cause treatment discontinuation rate between MPH and placebo in adults	Methylphenidate, All-cause treatment discontinuation	Database inception – January 2012	12	2,496	Random-effect meta-analysis	Included studies were of moderate quality No statistically significant difference was found between MPH and placebo for all-cause treatment discontinuation However, results showed a statistically significant difference when one outlier study was removed from the analysis (OR = 1.44, 1.14–1.82) implying higher treatment discontinuation for MPH Moderate heterogeneity (*I* ^2^ = 64%) was observed for treatment discontinuation	Long-term follow-up studies with large sample size are needed	High (8/9)
Peterson et al. [134]	Assess the effectiveness and safety of nonstimulants and longer-acting stimulants compared to conventional shorter-acting stimulants	Stimulants, Nonstimulants, Adults	Database inception – March 2007	22	2,203	Fixed and random-effect meta-analysis	Majority of included studies were of moderate quality Relative benefit of clinical response for IR-MPH, was greater (RR = 3.26, 2.03–5.22) than for patients taking longer-acting stimulants and also (RR = 2.24, 1.23–4.08) than longer-acting forms of bupropion	Additional high-quality, head-to-head trials are urgently needed to confirm the findings	High (9/9)
Cândido et al. [135]	Determine efficacy and adverse events of IR-MPH in adults	Immediate release methylphenidate, Adults	Database inception – January 2020	12	497	Narrative synthesis and random-effect meta-analysis	High risk of bias and significant heterogeneity between studies restricted from making any firm conclusion about the efficacy of IR-MPH, relative to placebo in treating ADHD symptoms	The long-term efficacy and risks of IR methylphenidate needs to be studied	High (9/9)
Castells et al. [136]	Assess the efficacy and safety of AMP in adults	Amphetamines, Placebo	Database inception – August 2017	19	2,521	Random-effect meta-analysis	High risk of bias, and significant heterogeneity restricted from making any firm conclusion about the efficacy and safety of AMP	In addition to studies that assess the long-term effect of amphetamines, head-to-head trials of AMP versus other ADHD medications in adults are also warranted	High (9/9)
**Nonstimulants**									
Cunill et al. [137]	Compare the efficacy and all-cause treatment discontinuation rate of ATX and placebo in adults	Atomoxetine, Placebo	Database inception – June 2012	12	3,375	Fixed and random-effect meta-analysis and meta-regression	ATX had small but significant efficacy in reducing ADHD symptoms regardless of who the raters were patient (SMD = −0.33,−0.43 to −0.23) or clinician (SMD = −0.40, −0.48 to −0.32) However, patients using ADX had greater odds for treatment discontinuation (OR: 1.39, 1.17–1.64) and discontinuation rate due to adverse events (OR: 2.57, 1.78–3.71) No heterogeneity was observed across the analysis	Long-term follow-up studies with enough statistical power are warranted	High (8/9)
Verbeeck et al. [138]	Assess the efficacy and safety of bupropion for the treatment of adults with ADHD	Bupropion, Placebo	Database inception – February 2017	6	438	Fixed effect meta-analysis	Included studies were of low quality Low-quality evidence was found for that bupropion decreased the severity of ADHD symptoms (SMD = 0.50, −0.86 to −0.15), and increased the proportion of participants achieving clinical improvement	Further research is needed to reach more definite conclusions as well as to assess the long-term outcome	High (9/9)
Manetton et al. [128]	Compare efficacy, acceptability, and tolerability of bupropion MPH	Bupropion, Methylphenidate	Database inception – January 2014	2	20	Random-effect meta-analysis	Insufficient data from the small number of studies and small sample size restricted from making any firm conclusion about possible differences in efficacy, acceptability, and tolerability between these medications	Further clinical trials should be conducted to provide firm conclusion	High (8/9)
**ADHD pharmacotherapy as a class**									
Elliott et al. [139]	Evaluate the relative effects of pharmacologic treatments for ADHD in adults	ADHD pharmacotherapy, Adults	Not clear	81	12,423	Network meta-analysis	ADHD pharmacotherapy, as a class, improved patient and clinical response relative to placebo However, no such efficacy was observed when including studies with low risk of bias Low quality of evidence and insufficient reporting of long-term adverse event, restricted from making any firm conclusion about the efficacy of the ADHD medications	Observational studies that assesss the long-term effects of ADHD medications in adults are needed	Moderate (7/9)
**Adverse effect of ADHD medication**									
Liang et al. [140]	Analyze the effects of ATX and MPH on HR, SBP, and a number of adverse cardiac events in children and adults	Atomoxetine, Methylphenidate, Heart rate; Systolic blood pressure	Database inception – May 31, 2016	22	46,107	Random-effect meta-analysis	Majority of the included studies were of moderate quality Significant increase in post-versus-pre-treatment HR was observed for children and adolescents (SMD = 1.56, 0.71–2.41) and adults (SMD = 1.40, 0.62–2.18) receiving MPH Similarly, children and adolescents treated with ATX had a greater increase in post-versus pre-treatment HR, compared to those treated with MPH (SMD = 0.86, 0.11–1.62) Significant heterogeneity (*I* ^2^ > 90%) was observed across the analysis	Additional research is needed that compare the long-term effects of these medications on heart rate and blood pressure	High (8/9)
Liu et al. [141]	Assess the association between ADHD medications and the risk of CVD cardiovascular diseases and mortality	Myocardial infarction, Stroke, Arrhythmia	Database inception – May 2018	8	4,221,929	Random-effect meta-analysis	Included studies were of high quality No significant association was found between ADHD medications and myocardial infarction, sudden death/arrhythmia, and all-cause death Moderate heterogeneity (*I* ^2^ = 56.8%) was observed across the analysis	Further studies using robust methodology that controls for all potential confounders and estimate dose-response relationship are required	Moderate (7/9)
Kidwell et al. [142]	Determine the effects of stimulant medications on sleep in youth with ADHD using objective measurement	Stimulant medications, sleep, ADHD youths	Database inception – March 2015	9	171	Random-effect and mixed-effect meta-analysis	Included studies were of high quality Stimulant medications were significantly associated with longer sleep latencies (*d* = 0.54, 0.28–0.81), poorer sleep efficiency (*d* = −0.39, −0.69 to −0.08), and reduced sleep duration (*d* = −0.59, −0.84 to −0.35) Moderators were number of doses per day (for sleep latency), treatment duration, total sleep assessment nights, use of polysomnography or actigraphy, and gender (for sleep efficiency) Moderate heterogeneity (*I* ^2^ = 67.3%) was observed for sleep latency, while significant heterogeneity (*I* ^2^ > 82%) was observed for sleep efficiency and sleep duration	Future studies should estimate the effects of stimulants on different sleep stages	High (8/9)
DeCrescenzo et al. [143]	Evaluate the effect of MPH on sleep characteristics using actigraphy	Methylphenidate, Actigraphy, Children	Database inception – June 2013.	8	Not given	Random-effect meta-analysis	Included studies were of high quality Children with ADHD taking MPH had longer sleep onset latency (SMD = 0.82, 0.38–1.26); and worse sleep efficiency (SMD = −0.33, −0.60 to −0.06) than those with placebo, as measured by actigraphy No heterogeneity was observed across the analysis	Measures that focus on reducing sleep problems among children using MPH should be considered	High (9/9)
Pan et al. [100]	Assess the effects of ADHD medications on headache	ADHD medications Headache	Database inception – June 2020	58	12,341	Random-effect meta-analysis	Majority of the included studies were of high quality ADHD medications were associated with increased headache during treatment periods, compared to placebo: ATX (OR = 1.29, 1.06–1.56), guanfacine (OR = 1.43, 1.12–1.82), and MPH (OR = 1.33, 1.09–1.63) No heterogeneity was observed across analyses	Clinical trials with large sample size using standardized method to detect treatment-related headaches, such as the Side Effects Rating Scale for stimulants are required	High (8/9)
Holmskov et al. [144]	Evaluate the effect of MPH on the risk of gastrointestinal adverse event	Methylphenidate, Gastrointestinal adverse event, Children and adolescents	Database inception – February 2015	61	5,983	Random-effect meta-analysis	Included studies were of poor quality MPH was found to increase the risk of reduced appetite (RR = 3.66, 2.56–5.23); weight loss (RR = 3.89, 1.43–10.59), abdominal pain (RR = 1.61, 1.27–2.04), compared to placebo No significant difference was found according to type, dose, or length of treatment No heterogeneity was observed across the analysis	Further clinical trials assessing the long-term effect of MPH on the gastrointestinal adverse events are needed	High (9/9)
Chierrito de Oliveira et al. [145]	Evaluate the safety profile of ADHD medications	ADHD treatment, Adult	Database inception – September 2017	10	3,006	Pair-wise and network meta-analysis	Majority of the included studies were of high quality Compared to placebo, ATX (OR = 0.15, 0.05–0.38) and extended-release mixed AMP salts (OR = 0.06, 0.00–0.51) were significantly associated with loss of appetite	Apart from assessing the efficacy and safety of ADHD medications, research should also conduct a cost-effectiveness analysis of these medications	Moderate (7/9)
Kittel-Schneider et al. [78]	Synthesize recent evidence on the risks of stimulant and nonstimulant treatment for ADHD in pregnancy and lactation	Pregnancy, Lactation, ADHD medications	Database inception – November 2021	25	29,282 exposed and 11,452,476 controls for six medication exposures	Narrative synthesis	Majority of the included studies were of moderate quality As little safety data were available for ATX, guanfacine, and modafinil, they cannot be recommended for prescription during breastfeeding	Longitudinal large studies using robust design, for example, example sibling designs and including as many as possible confounders and variables are needed to strengthen the evidence	Moderate (6/9)
Li et al. [146]	Examine the potential effect of exposure to ADHD medication during pregnancy on pregnancy and offspring related outcome	ADHD medications, Pregnancy, Offspring	Database inception – July 2019	8	More than 2 million	Narrative synthesis	Majority of the included studies were of moderate quality No association seemed to exist between exposure to ADHD medications during pregnancy and adverse maternal or offspring outcomes Inadequate and inconsistent adjusting for potential confounders were observed across included studies, and none of the included research took into account the severity of ADHD symptoms among mothers	Future studies should account for all potential confounders and assess the effect of ADHD medication during different stages of pregnancy and on long-term outcomes in offspring	High (8/9)
Jiang et al. [147]	Identify association between exposure to ADHD medication during pregnancy and adverse outcome	Pharmacoepidemiology, Prenatal	Database inception – May 2018	8	Not given	Fixed and random-effect meta-analysis	Included studies were of high quality Exposure to ADHD medication was associated with an increased risk of admission to neonatal intensive care unit (NICU) compared with control(risk ratio (RR) = 1.88, 1.7–2.08) No association was found between the use of ADHD medication during pregnancy and any adverse maternal or neonatal outcome Moderate heterogeneity (*I* ^2^ = 52%) was observed for NICU analysis	Larger cohort studies that adjust for potential confounders are needed to gain better insight	Moderate (7/9)
**Adverse events of MPH treatment**									
Storebø et al. [148]	Identify the adverse effect of MPH in nonrandomised studies	Methylphenidate, Adverse events, Children and adolescents	Database inception – January 2016	260	>2,207,751	Random-effect meta-analysis	Very low-quality of evidence and low reliability of the evidence existed for the association between MPH use and risk of adverse events in children and adolescent	High quality, large-scale RCTs along with studies that are aimed towards identifying individuals who responds to treatment versus those who do not respond are needed	High (9/9)
**Efficacy of medication on ADHD symptoms in patients with comorbid conditions**									
Rodrigues et al. [149]	Assess the efficacy and safety of pharmacological treatment on ADHD symptoms among patients with ASD	Pharmacotherapy, ASD, Children and youth	1992–April 2018	25	2,606	Narrative synthesis and random-effect meta-analysis	Majority of the inlcuded studies were of moderate quality Compared to placebo, MPH showed moderate to greater efficacy in treating hyperactivity (SMD on parent rating = −0.63, −0.95 to −0.30 and on teacher rating = −0.81, −1.43 to −0.19), small but significant efficacy on inattention (SMD on parent rating = −0.36, −0.64 to −0.07 and on teacher rating −0.30¸ −0.49 to −0.11) ATX also showed small but significant efficacy against hyperactivity (SMD on parent rating = −0.49, −0.76 to −0.23 and on teacher rating = −0.43; −0.92 to −0.06), and inattention (SMD on parent rating = −0.54, −0.98 to −0.09 and on teacher rating = −0.38, −0.75 to −0.01) Moderate heterogeneity (*I* ^2^ > 50%) was observed across analysis	Further research on long-term effect of medication on clinical symptoms including real-life outcomes (education, employment, cognitive) are needed	Moderate (6/9)
Sun et al. [150]	Examine effects of MPH for ADHD among children with ID or borderline intellectual functioning	Methylphenidate, ADHD, Intellectual disability	Database inception – August 2018	8	423	Random-effect meta-analysis and meta-regression	Majority of the included studies were of poor quality MPH showed greater efficacy improving ADHD symptoms than placebo (Hedges’ *g* = 0.87; 0.61–1.14) Meta-regression analysis showed significant association between changes in overall ADHD severity and methylphenidate dosage However, there was no significant difference between MPH and placebo for drop-out rate or discontinuity of treatment Moderate heterogeneity (*I* ^2^ = 42%) was observed across analysis	Large-scale randomized controlled trials assessing the efficacy and tolerability of MPH in children with comorbid ADHD and ID or borderline intellectual function are needed	High (8/9)
Tarrant et al. [151]	Examine the beneficial effect of methylphenidate in reducing ADHD symptoms among individuals with intellectual disabilities	Methylphenidate, Intellectual disabilities, Children and adolescents	Database inception – August 5, 2017	15	Not given	Narrative synthesis	Small sample size and high risk of bias across studies restricted from making any firm conclusion about the effect of MPH on reducing ADHD symptoms among those with intellectual disabilities	Clinical trials with sound methodological design that are not sponsored by pharmaceutical companies and that includes both children and adult population with ID and ADHD are needed	Moderate (6/9)
Froehlich et al. [152]	Asssess the effectiveness of FDA-approved ADHD medications on ADHD and comorbid reading disorder	Methylphenidate, Atomoxetine, Pediatric	Not clear	14	371	Narrative synthesis	Included studies were of moderate quality MPH and ATX were found to have potential in improving both core ADHD symptoms as well as specific skills related to academic achievement in reading and math in children with ADHD and comorbid reading disorder	Studies assessing the long-term effect of medications are needed	Moderate (7/9)
Osland et al. [153]	Examine the effects of medications for ADHD in children with comorbid tic disorder	Medications, ADHD, Tic disorder	Database inception – September 2017	8	510	Narrative synthesis	Small number of studies, small sample size, and high risk of bias across included studies restricted from making any firm conclusion about the effects of MPH, clonidine, gunfacine, despiramine, and ATX in treating ADHD symtopms among those with comorbid tic disorder	Further studies with robust methodology including larger sample are needed	High (8/9)
Woon et al. [154]	Evaluate the effectiveness of medication on comorbid ADHD and stimulant dependence	Pharmacotherapy, Stimulant dependence, Adult	Database inception – June 2017	5	358	Narrative synthesis	Evidence about the effectiveness of pharmacotherapy for comorbid ADHD and stimulant dependence were preliminary and promising, but mixed	High-quality research with sufficient statistical power is needed to draw firm conclusion	High (8/9)
Anand et al. [155]	Evaluate the quality of evidence about the efficacy, safety, and tolerability of medications used in treating behavioral insomnia among ADHD children	ADHD, Behavioral insomnia, Pharmacological treatment	Database inception – February 2017	12	Not given	Narrative synthesis	Melatonin showed some improvement in sleep-onset latency, and total sleep duration However, small sample size, high risk of bias, and heterogeneity across study restricted from making firm conclusion for the use of medications in treating behavioral insomnia among ADHD children	Well-designed clinical trials with adequate statistical power are required	Moderate (7/9)
Tsujii et al. [156]	Summarize the efficacy and safety of treatments for children and adolescents with ADHD and common comorbidities	Comorbidities Pharmacotherapy	Database inception – October 2019	69	500–1,000	Narrative synthesis	Majority of the included studies were of high quality Limited information is available from placebo-controlled RCTs on the efficacy (by ADHD-RS-IV) or safety of medication in children with ADHD and psychiatric comorbidities (ASD, ODD, Tourette’s disorder and other tic disorders, generalized anxiety disorder, and major depressive disorder)	Further studies are required to support evidence-based drug selection for these populations	Moderate (6/9)
Thomson et al. [157]	Examine the effectiveness of amphetamine for the treatment of ADHD among those with intellectual disabilities	Amphetamine, Intellectual Disabilities	Database inception – August 2007	1	15	Narrative synthesis	Included study was of moderate quality Lack of sufficient studies restrict to make firm conclusion about the efficacy of amphetamine	–Further high-quality RCTs are urgently needed	High (9/9)
Thomson et al. [158]	Examine the effectiveness of risperidone for the treatment of ADHD among those with intellectual disabilities (ID)	Risperidone, Intellectual Disabilities	Database inception – February 2009	11 considered but none were suitable for inclusion	NA	Narrative synthesis	There is no evidence from RCTs that risperidone is effective for the treatment of ADHD in people with ID	Research into effectiveness and tolerability is urgently needed	High (9/9)
**Efficacy of medication on comorbidity and other consequences of ADHD**									
Fluyau et al. [159]	Evaluate the beneficial effect of pharmacotherapy to treat SUD in ADHD patients	Pharmaceutical intervention, SUD, ADHD	1971–2020	17	2,155	Random-effect meta-analysis	Majority of the included studies were of poor quality Pharmacotherapy induced modest reduction on substance use (SMD = 0.40, 0.25–0.55), abstinence (SMD = 0.33, 0.15–0.50), craving (SMD = 0.27,0.10–0.44), and frequency of ADHD symptoms (SMD: 0.42, 0.25–0.58) Likewise, medications were moderately efficacious in the management of substance use withdrawal symptoms (SMD = 0.57, 0.38–0.76) and reduction in ADHD severity symptoms (SMD = 0.53, 0.39–0.67) Moderate heterogeneity (*I* ^2^ = 35.3%) was observed for substance use	Further clinical trials that apply uniform outcome measurement scale and diagnostic criteria are required, in addition to focusing on the neurobiological mechanism that links or differentiate both disorders	High (8/9)
Man et al. [160]	Estimate the association between ADHD medications and physical injury	Pharmacological treatment, ADHD, Physical injuries	Database inception – May 2017	10	751,602,319	Random-effect meta-analysis	Majority of the included studies were of high quality ADHD medications significantly reduced risk of injuries For within-individual method, rate ratio was 0.76 (0.61–0.93), and for between-individual studies, rate ratio was 0.88 (0.85–0.92) Significant heterogeneity (*I* ^2^ > 90%) was observed across analysis	Further studies need to assess the causal effect of ADHD medication on risk of physical injuries	Moderate (6/9)
Zhang et al. [161]	Assess effect of ADHD medication and the risk of fracture in ADHD patients	Stimulants, Nonstimulants, Fracture	Database inception – December 2020	10	>10,000	Random-effect meta-analysis	Majority of the included studies were of high quality No significant association, but a trend toward a lower risk, was observed for stimulant-treated ADHD patients compared with nonstimulant-treated ADHD patients Nonstimulant-treated ADHD was associated with traumatic fracture (OR = 1.79, 1.54–2.08) or stress fracture (OR = 1.12, 1.04–1.20) compared to healthy control Significant heterogeneity (*I* ^2^ > 90%) were observed across analyses	Future research should comprise analyses based on an accurate definition of ADHD and fracture	High (8/9)
Schoenfelder et al. [162]	Evaluate the beneficial effect of stimulant medication for ADHD on smoking	ADHD, Psychostimulant medication, Cigarette smoking	Database inception – July 2013	17	2,804	Fixed-effect and random-effect meta-analysis and meta-regression	Majority of the included studies were of high quality Stimulant medications were significantly associated with reduced cigarette smoking rate in ADHD patients and the effect size was greater for observational studies with clinical and fewer male samples, consistent treatment, measured smoking outcomes in adolescents rather than in adults, and adjusted for comorbid conduct disorder Moderate heterogeneity (*I* ^2^ > 60%) was observed across the analysis	Research that assess causal relationship between ADHD stimulants and smoking behavior is needed	High (9/9)
Liu et al. [163]	Evaluate association between ADHD medication and risk of suicidal attempt	Pharmacoepidemiology, Central stimulants, Suicidal behavior	Database inception – February 2020	7	4,790,600	Random-effect meta-analysis	Included studies were of high quality Treatment with stimulants was associated with a lower risk of suicide attempt among children and adolescents with ADHD as shown by both population-level analysis (RR = 0.72, 0.53–0.99), and within-individual analysis (RR = 0.75, 0.66–0.84) However, the association was not significant for those who were exposed to stimulants for a short period of time, that is, 1–90 days (RR = 0.91, 0.74–1.13) Significant heterogeneity (*I* ^2^ > 90%) was observed across the analysis	Multinational studies using uniform assessment methods that adjust for all potential confounders are needed Further studies on the adult population are also warranted	Moderate (7/9)
Prasad et al. [164]	Evaluate the effectivenss of medication on improving ADHD children task-behavior and academic performance	Medication, Education, Achievement	Database inception – June 2010	43	2,110	Narrative synthesis and random-effect meta-analysis	Included studies were of moderate quality ADHD medications like MPH, dexAMP, and mixed AMP formulations have positive effects on ADHD children’s classroom task performance and academic performance. However, no significant effect was observed for ATX Significant heterogeneity (*I* ^2^ > 85%) was observed across analysis	Head-to-head trials of medications are required to compare the superiority of one medication over another in improving academic achievement among ADHD individuals	High (9/9)
Hulsbosch et al. [165]	Assess the effects of medication, on instrumental learning in children with ADHD	Instrumental learning, ADHD	Database inception – March 2020	3	Not given	Narrative synthesis	Included studies were of high quality Findings revealed a positive effect of MPH but no effect of fluoxetine compared to placebo in children with ADHD	Further studies with large sample size and high statistical power are required	High (8/9)
Pievsky et al. [166]	Assess the beneficial effect of MPH on neurocognitive performance	ADHD adults, Methylphenidate, Neurocognition	Database inception – November 2016	21	832	Random-effect meta-analysis and meta-regression	Majority of the included studies were of moderate quality MPH was associated with small but significant improvement on neurocognitive driving capacitiy, compared to placebo (Hedges’*g* = 0.17, 0.05–0.28) MPH also showed small but significant improvement on working memory (Hedges’*g* = 0.13, 0.00–0.26), reaction time variability (Hedges’*g* = 0.16, 0.03–0.28); vigilance (Hedges’*g* = 0.22, 0.11–0.33), driving (Hedges’*g* = 0.22, 0.10–0.34), and response inhibition (Hedges’*g* = 0.23, 0.10–0.36) No heterogeneity was observed across the analysis	Further research with high statistical power is needed	High (8/9)
**Effect modification of comorbid conditions and drug effects on cooccurring symptoms**									
Villas-Boas et al. [167]	Assess the effectiveness of pharmacotherapy on ADHD and comorbid AD	Drug therapy, Safety, Children and adolescents, Adults	Database inception – July 2018	5	Not given	Narrative synthesis	Quality of included studies ranged from moderate to high ATX was found to be efficacious in treating ADHD patients comorbid with AD	Larger multi-center clinical trials with long-term follow-up are needed to clarify the present findings	High (8/9)
Lenzi et al. [168]	Evaluate the effects of medications on ED in adults with ADHD	Stimulants, Emotional dysregulation, ADHD	Database inception – April 1, 2017	21	4,940	Random-effect meta-analysis	Majority of the included studies were of moderate quality MPH (SMD = −0.34, −0.45 to −0.23), ATX (SMD = −0.24, −0.34 to −0.15), and lisdexamfetamine (SMD = −0.50, −0.80 to −0.21) had small but significantly efficacious in treating ED Moderate heterogeneity (*I* ^2^ > 35%) was observed for MPH and lisdexamfetamine	Further studies assessing the effect of ADHD medications on ED are required	High (9/9)
Connor et al. [169]	Assess the efficacy of stimulants on overt and covert aggression-related behaviors in children with ADHD	ADHD, Aggression	1970–2001	28	683	Random-effect meta-analysis	Majority of the included studies were of moderate quality Stimulants were efficacious in reducing both overt (Cohen’s *d* = 0.84, 0.69–1.02) and covert aggression (*d* = 0.69,0.21–1.29) compared to placebo in children and adolescents with ADHD Comorbid conduct disorder reduced stimulant effect size for overt aggression	Additional research into the effectiveness of medications for aggression-related behaviors in clinically referred youths are needed	High (8/9)
Pringsheim et al. [170]	Assess the therapeutic effects of antipsychotics and traditional mood stabilizers for aggression and conduct problems in youth with ADHD, ODD, and CD	Antipsychotics, Mood stabilizers, Youth	Database inception – October 2013	11	500–2,000	Random-effect meta-analysis	Majority of the included studies were of moderate quality Risperidone was moderately efficacious (SMD = 0.60, 0.31–0.89) in treating conduct problems and aggression in youth with subnormal intellectual functioning and ODD, CD, or disruptive behavior disorder not otherwise specified, with and without ADHD No heterogeneity was observed across the analysis	Long-term follow-up studies are needed to assess the long-term safety and efficacy of medications	High (9/9)
**Pharmacogenetics and animal models**									
Yuan et al. [171]	Evaluate whether noradrenergic gene polymorphisms impact the efficacy of MPH in children with ADHD	Methylphenidate, Noradrenergic gene polymorphism	Database inception – March 2020	15	1,382	Random-effect meta-analysis and meta-regression	Included studies were of high quality T allele carriers of the rs28386840 polymorphism were significantly more likely to respond to MPH (OR = 2.05,1.32–3.19) Significant heterogeneity (*I* ^2^ > 75%) was observed across the analysis	Further investigations with larger samples will be needed to confirm these results	Moderate (7/9)
Soleimani et al. [172]	Evaluate the potential role of SLC6A3 polymorphisms in response to MPH in children with ADHD	MPH, Genetic, Child and adolescent psychiatry	Database inception – May 1, 2017	16	1,357	Random-effect meta-analysis and meta-regression	Quality of included studies ranged from moderate to poor Results from naturalistic studies demonstrated an association between non 10/10 repeat carriers and greater response to MPH (Cohen’s *d* = 0.44, 0.12–0.75), while results from clinical trials were insignificant for both 10/10 and 9/9 repeat polymorphism However, the quality of evidence was poor	Further research that studies gene–gene interaction and elucidate the role of potential confounders or effect modifiers are required	High (9/9)
Leffa et al. [173]	Assess the behavioral effect of MPH in spontaneously hypertensive rat (SHR)	MPH, Animal model, SHR	Database inception – February 2017	36	657	Random-effect meta-analysis and meta-regression	Majority of the included studies were of poor quality MPH did not reduce hyperactivity in SHR as an animal model of ADHD Significant heterogeneity was observed across analysis Significant heterogeneity (*I* ^2^ > 70%) was observed across the analysis	For human treatment to be more accurate, further research on animal models will need to be conducted. Such studies will include doses analogous to those commonly used in humans	High (9/9)
**Pharmacological treatment rate**									
Massuti et al. [174]	Estimate the rate of ADHD pharmacological treatment in both diagnosed and undiagnosed children and adolescents	Prevalence, Treatment, Pharmacological interventions	Database inception – April 2020	36	104,305	Random-effect meta-analysis and metaregression	Majority of the studies were of moderate quality The pooled pharmacological treatment rate for the DSM/ICD ADHD diagnosis group (18 studies; *n* = 3,311) was 0.19 (0.11–0.29) In non-ADHD group (14 studies; *n* = 29,559), the pooled ADHD medication use rate was 0.009 (0.005–0.017) In US, for each individual using medication without a formal ADHD diagnosis, there are three patients with a formal diagnosis who might benefit from medication but do not receive it In Australia, there exists 11-fold difference between under pharmacological treatment and overtreatment/misuse rates, while in Netherlands the difference is less than twofold Significant heterogeneity (*I* ^2^> 96%) was observed across analysis	Further medical and parental education on ADHD is needed worldwide, as part of public health policies	High (8/9)

Abbreviations: AMP, amphetamine; ASD, autism spectrum disorder; ATX, atomoxetine; CVD, cardiovascular disease; d-MPH, dex-methylphenidate; dex-AMP, dex-amphetamine; ED, eating disorder; HR, heart rate; IR-MPH, immediate-release methylphenidate; *k*, total number of included studies; LDX, lisdexamphetamine; MPH, methylphenidate; NA, not applicable; OR, odds ratio; OROS, osmotic release oral system; SBP, systolic blood pressure; SMD, standardized mean difference; SUD, substance use disorder; WMD, weighted mean difference.

a Total participants included in the systematic review and meta-analysis unless otherwise indicated.

b For findings from meta-analysis, if given effect estimates with 95% CI are presented or otherwise specified.

### Nonpharmacological treatment (n = 42, Table 9)

#### Children and adolescents

Behavioral interventions, including social and academic skills training, cognitive behavioral therapy (CBT), parent coaching on social skills, and stress management, were found to decrease the child’s ADHD symptoms and conduct problems and improve social skills, academic performance, and positive parenting when reported by proximal observers [175]. Effects on parenting and children’s conduct problems persisted when assessment was blinded [175]. Parental training was an efficacious intervention for reducing ADHD symptoms in preschool children (Hedges’ *g* = 0.51, 95% CI: 0.33–0.65) and negative parenting style, as based on parents’ rating [176].

In a school-based setting, combined interventions, including social skills training, behavior modification technique, study and organizations skills training, were found to improve ADHD symptoms, as rated by both teachers and parents [177]. Daily behavior report cards were associated with reductions in teacher-rated ADHD symptoms [178] and improvement in academic outcomes [177]. Peer-inclusion interventions led to moderate improvements in pre-post measure of social functioning (Hedges’ *g* = 0.58, 0.45–0.70) among those receiving treatment [179].

Results were mixed for the efficacy of cognitive interventions in reducing ADHD symptoms and for improving working memory performance [180–182]. Virtual reality-based interventions were more effective than other nonpharmacological interventions or no intervention in improving sustained attention in children and adolescents with ADHD [183]. Interventions based on mind–body therapies [184] and few-foods diet, that is diet elimination of many foods and additives, have shown positive effects on ADHD core symptoms [185]. However, homeopathy did not show positive effects in reducing ADHD symptoms [186].

Several reviews suggested that despite their wide applications, significant knowledge gaps exist regarding the effectiveness of various nonpharmacological interventions. These include mindfulness [187–189], neurofeedback [190–193], behavioral interventions [193], cognitive training [193], dietary interventions [194–201], herbal interventions [202, 203], parent and teacher training [204, 205], social skills training [206], school-based interventions [207], equine-assisted therapies [208, 209], video modeling [210], acupuncture [211], and physical exercise [212].

#### Adults

In adults, long-term, that is at least 12 months follow-up of psychotherapies (CBT, dialectical behavioral therapy, mindfulness-based cognitive therapy) showed greater improvement in self-reported total ADHD symptoms (SMD = 0.71, 95% CI: 0.22–1.21), inattention, and hyperactivity/impulsivity in intervention than control groups [213]. One systematic review suggested that CBT might improve the core symptoms of ADHD, but the evidence was of low quality [214]. CBT was also efficacious in treating comorbid-internalizing symptoms in adults with ADHD [215]. Mind–body therapies including meditation were efficacious in reducing ADHD core symptoms compared to, for example, treatment as usual, although the evidence was of low quality [216].

Evidence for behavioral intervention to improve driving skills in young train novice drivers with ADHD was inconclusive [217]. Transcranial direct current stimulation might have some effect on neuropsychological and cognitive deficits, but there was no evidence to suggest that it decreases ADHD symptoms [218].

**Table 9.** Nonpharmacological treatment

References	Objective	Keywords	Timeframe of database search	*k*	Sample size^a^	Analytical design	Main findings (Effect estimates, 95% CI)^b^	Conclusion and comments from the authors	Quality assessment
**Children and adolescents**									
Daley et al. [175]	Assess effectiveness of behavioral interventions (behavioral, social, academic skills training, CBT) in ADHD	ADHD, Parenting, Intervention	Database inception – February 2013	32	500–2,000	Random-effect meta-analysis	– Majority of included studies were of moderate quality – Behavioral interventions showed small beneficial efficacy in reducing child ADHD symptoms (SMD = 0.35, 0.19–0.50), social skills (SMD = 0.47, 0.15–0.78), academic performance (SMD = 0.28, 0.06–0.50), and positive parenting (SMD = 0.68, 0.28–1.09) – With probably blinded assessments, significant moderate effects persisted for parenting (SMD for positive parenting 0.63, SMD for negative parenting 0.43) – Significant heterogeneity (*I* ^2^ > 80%) was observed across analysis	There is a need for further analysis of the moderating effects of child’s age on intervention outcomes, and proximal outcomes such as academic performance and social skills should be confirmed by blinded assessment	Moderate (7/9)
Rimestad et al. [176]	Assess the effectiveness of parent training for preschool children with ADHD or ADHD symptoms	Parent training, early intervention	Database inception – May 2015	16;	1,003	Random-effect meta-analysis	– Included studies were of moderate quality – Based on parental report of ADHD, parental training was moderately efficacious in reducing ADHD symptoms (Hedges’s *g* = 0.51, 0.33–0.65) and negative parenting (Hedges’s *g* = 0.63, 0.32–0.93) – In depended-rated outcomes revealed small beneficial effect only for negative parenting (Hedges *g* = 0.33, 0.13–0.53) – Significant heterogeneity (*I* ^2^ > 70%) was observed across analysis	Future studies should assess the long-term effect of such interventions on ADHD	High (8/9)
Moore et al. [177]	Determine the effectiveness of school-based non -pharmacological interventions for ADHD	School-based, Children	1980–January 2018	30	1,807	Narrative synthesis and random-effect meta-analysis	– Most of the included studies were of low quality – Combined intervention (social skills training, behavior modification technique, etc.) showed greater efficacy in ADHD combined symptoms rated by both teachers (Hedges’ *g* = 0.79, 0.45–1.12) and parents (*g* = 0.97, 0.45–1.12) – School-based combined interventions showed small beneficial effect in improving both teacher-rated (Hedges’ *g* = 0.30, 0.12–0.7) and parent-rated (Hedges’ *g* = 0.37, 0.19–0.55) academic outcomes – Moderate heterogeneity (*I* ^2^ > 60%) was observed across analysis	Interventions should be evaluated across different age groups and degrees of ADHD severity	Moderate (7/9)
Iznardo et al. [178]	Synthesize the effectiveness of DBRC for ADHD children	School-based intervention, Daily Behavior Report Cards, Teachers	Database inception- November 2015	24	272	Narrative synthesis and random-effect meta-analysis	– DBRCs showed small beneficial effect in reducing ADHD symptoms in classroom (teacher-rated Hedges’s *g* = 0.36, 0.12–0.60) – No heterogeneity was observed across analysis	Methodologically rigorous studies investing mediator and moderator variables that may influence the effectiveness of this intervention need to be conducted	Moderate (6/9)
Cordier et al. [179]	Determine the impact of peer inclusion in interventions for ADHD children	Peer inclusion	Database inception – 2016	17	2,567	Random-effect meta-analysis	– Included studies were of moderate to low quality – Peer inclusion (peer involvement or peer proximity) interventions in addition to pharmacological treatment was found to be beneficial in improving social competence and peer relation compared to treatment as usual or no treatment – Moderate heterogeneity (*I* ^2^ > 45%) was observed across analysis	Trials that use peer-mediated interventions, and control for medication as potential confounder are needed	High (8/9)
Chen et al. [180]	Assess the effect of cognitive intervention on symptoms and executive function behaviors of children with ADHD	Cognitive training, Executive function	Not given	17	1,075 (904 for effect estimates)	Random-effect meta-analysis	– Majority of included studies were of low quality – When all of the training are considered together, cognitive training can improve the presentation of inattention symptoms [SMD = −0.39, (−0.67, −0.10) and executive function behaviors (SMD = −0.31, (−0.52, −0.11)] – Effects of working memory training on both presentations were not statistically significant – Significant heterogeneity (*I* ^2^ > 75%) were observed across analyses	Further research is needed to confirm and extend these psychology fields	High (8/9)
Pauli-Pott et al. [181]	Assess the effectiveness of cognitive interventions on executive function and ADHD in pre-school children	Executive function, Cognitive interventions	Database inception- April 2018	35 (29 in meta-analysis)	3,068	Narrative synthesis and random-effect meta-analysis	– Included studies were of moderate quality – Cognitive scaffolding interventions were most effective in terms of reducing ADHD symptoms	Additional well-controlled clinical trials with large sample size are required	Moderate (6/9)
Cortese et al. [182]	Investigate the effectiveness of cognitive training on ADHD	Working memory, Executive functions	Database inception – May 2015	15	826	Random-effect meta-analysis	– Included studies were of high to moderate quality – According to blinded assessment, cognitive training improved working memory but did not seem to reduce ADHD symptoms – No significant heterogeneity was observed across analysis	Long-term studies with wider range of functional outcomes are required	Moderate (7/9)
Romero-Ayuso et al. [183]	Determine the effectiveness of virtual reality-based interventions (VR based interventions) on cognitive deficits in children with ADHD	Virtual reality, Rehabilitation	Database inception – October 2020	4	125	Random-effect meta-analysis	– Included studies were of moderate to low quality – The magnitude of the effect was large for omissions (SMD = −1.38, *p* = 0.009), correct hits (SMD = −1.50, *p* = 0.004), and perceptual sensitivity (SMD = −1.07, *p* = 0.01); and moderate for commissions (SMD = −0.62, *p* = 0.002) and reaction time (SMD = −0.67, *p* = 0.03) – Significant heterogeneity (*I* ^2^ > 80%) were observed across analyses	Additional well-controlled clinical trials with large sample size are required	High (8/9)
Barranco-Ruiz et al. [184]	Synthesize the effectiveness of mind–body therapies interventions on ADHD	Mind–body therapies, Relaxation therapies, Children and adolescents	2000–2018	12	311	Narrative synthesis	– Most of the included studies were of low quality – Most of the included studies found that mind–body therapies were effective in reducing ADHD symptoms in children and adolescents – Considerable conceptual heterogeneity was observed across studies	To confirm the existing findings, RCTs should include representative samples with greater statistical power	High (8/9)
Pelsser et al. [185]	Determine the effectiveness of diet on ADHD	Dietary-based interventions, Nonpharmacological treatment, Children and adolescents	Database inception-December 2015	6	1,937	Random-effect meta-analysis	– Most of the included studies were of moderate quality – The average FFD showed greater efficacy (Parent-rated SMD = 0.80, 0.41–1.19 in reducing ADHD symptoms – The effectiveness of polyunsaturated fatty acid supplementation and artificial food color elimination on ADHD was negligible – Moderate heterogeneity (*I* ^2^ > 60%) was observed across analysis	Further FFD research should focus on establishing the underlying mechanisms of food (e.g., incrimination of gut microbiota) to simplify the FFD approach in children with ADHD	High (8/9)
Heirs et al. [186]	Assess the efficacy and safety of homeopathy	ADHD, Homeopathy	Database inception – March 2006	4	168	Random-effect meta-analysis	– Included studies were of low quality – No evidence exist about the effectiveness for homeopathy for the global symptoms, core symptoms or related outcomes of ADHD	Optimal treatment protocols needs to be developed before conducting further randomized controlled trials	High (9/9)
Oliva et al. [187]	Assess the effects of MBIs on either ADHD and associated features, associated clinical conditions, neurocognitive impairments, mindfulness skills, global functioning and quality of life	Mindfulness-based interventions	Database inception- June 2020	15	412	Narrative synthesis	Smaller number of studies, sample size, poor quality of included studies and lack of active-control studies restricted from making any firm conclusion about the efficacy of MBIs on reducing ADHD core symptoms and other concerning outcomes	The low general methodological quality highlights the need to conduct more active-controlled studies, on larger sample sizes with measurement at follow-up	High (9/9)
Evans et al. [188]	Assess the effect of meditation-based interventions on ADHD	Meditation, Children and adolescents, Parents	Database inception – March 2017	16	>416	Narrative synthesis	Low quality of included studies and high heterogeneity restricted from making any firm conclusion about the efficacy of symptoms meditation-based interventions in reducing ADHD symptoms	RCTs with adequate power are needed	Moderate (6/9)
Zhang et al. [189]	Address the effectiveness of meditation-based interventions on ADHD patients	Meditation-based interventions, Children and adolescents, Adults	Database inception – May 5, 2018	13	270	Random-effect meta-analysis	Small number of RCTs, low quality of included studies and high heterogeneity across studies restricted from making any firm conclusion about the efficacy of meditation-based interventions on ADHD	Future RCTs should be of high quality, with larger sample size, have uniform definition of control group and with long-term follow-up period	High (9/9)
SampedroBaena et al. [190]	Analyze the effects of NF interventions in children with ADHD	Neurofeedback, Treatment	2017–June 2021	9	620	Narrative synthesis	– NF showed a significant improvement of the symptoms in children with ADHD	Additional randomized controlled trials are needed to determine the significant effects	Moderate (6/9)
Goode et al. [191]	Compare the effectiveness of nonpharmacologic treatments for ADHD	Nonpharmacological interventions, Children and adolescents	January 2009–November 2016	54	353	Narrative synthesis	Low quality of included studies and significant heterogeneity restricted from making any firm conclusion about the efficacy of different nonpharmacological interventions (CBT; child or parent training, neurofeedback, herbal or dietary approach) on ADHD	Pragmatic RCTs with long-term follow up and adequate sample size are required	Moderate (6/9)
Cortese et al. [192]	Assess the effectiveness of NF on ADHD symptoms	Neurofeedback, Nonpharmacological treatment, Children and adolescents	Database inception – August 2015	13	520	Random-effect meta-analysis	Meta-analysis of blinded outcomes assessment showed no significant effect of NF on ADHD symptoms or other cognition covariates	Research should determine the most suitable electro-physiological treatment targets, develop standardized EEG and learning protocols, and identify predictors of treatment response for individual patients or group of patients	High (8/9)
Sonuga-Barke et al. [193]	Assess the impact of nonpharmacological interventions on ADHD	Dietary-based interventions, Psychological treatment, Children and adolescents	Database inception – April 2012	54	3,215	Random-effect meta-analysis	Based on probably blinded assessment, free fatty acid supplementation was found to have a significant but small effect on ADHD symptoms (SMD = 0.17, 0.01–0.34), while evidence for other nonpharmacolgical interventions (blinded assessments is required for behavioral interventions, neurofeedback, cognitive training, and restricted elimination diet) were limited	Further clinical trials using blinded assessment are required to assess the efficacy of nonpharmacological interventions like behavioral interventions, neurofeedback, cognitive training, and restricted elimination diet on ADHD symptoms	High (9/9)
Händel et al. [194]	Assess the efficacy of PUFAs in the treatment for ADHD	Fatty acids; omega 3; polyunsaturated	Database inception – June 2020	31	1,755	Random-effect meta-analysis	– Majority of included studies were of poor quality – PUFAs did not showed any effect The results showed no effect on ADHD core symptoms rated by parents (SMD = −0.17, −0.32 to −0.02) or teachers (SMD = −0.06, −0.31 to −0.19) – Moderate heterogeneity (*I* ^2^ > 50%) was observed across analysis	Further high quality RCTs are required to make firm conclusion	High (8/9)
Gillies et al. [195]	Evaluate the effectiveness of PUFA in treating ADHD symptoms	Polyunsaturated fatty acid, Nonpharmacological treatment, Children and adolescents	Database inception – August 2011	13	1,011	Fixed and random-effect meta-analysis	PUFA supplementation did not appear to be beneficial in most studies, although some studies showed some benefits with a combination of omega-3 and omega-6 supplementation	Future trials should include larger sample size and use a robust methodology to lower the risk of bias	High (8/9)
Bloch et al. [196]	Evaluate the efficacy of omega-3 fatty acid supplementation in ADHD	Polyunsaturated fatty acids, Omega-3 fatty acids, Children and adolescents	Database inception – December 2010	10	699	Random-effect meta-analysis	Omega-3 fatty acid showed a small, but a significant effect on ADHD symptoms (SMD = 0.31, 0.13–0.47). However, small sample size and low quality of included studies restricted from making any firm conclusion about its efficacy in reducing ADHD symptoms	Future trials should include larger sample size and supplements with a high concentration of EPA, an omega-3 fatty acid, to determine the dose–response relationship	Moderate (7/9)
Abdullah et al. [197]	Evaluate the efficacy of omega-3 fatty acid supplementation in ADHD	Polyunsaturated fatty acids, Omega-3 fatty acids, Children and adolescents	Database inception – February 2018	7	926	Narrative synthesis	Small number of RCTs and small sample size restricted from making any firm conclusion about the efficacy of omega-3 fatty acid supplementation in treating ADHD	Future research should include larger sample size and be based on long-term follow-up period	High (9/9)
Cooper et al. [198]	Assess the effectiveness of omega-3 polyunsaturated fatty acid supplementation on ED, oppositional behavior, conduct problems and aggression in children with ADHD	Omega-3, Emotional liability, Oppositional behavior	Database inception – 2014	12	500–2,000	Random-effect meta-analysis	PUFA supplementation did not show improvements in measures of ED, oppositional behavior, conduct problems or aggression. in children with ADHD	Future studies need to include larger sample size and high concentration of *n* − 3 PUFA supplements to identify dose–response relationship	High (9/9)
Talebi et al. [199]	Determine the efficacy of zinc supplementation on ADHD	Zinc, Clinical trials	Database inception – January 2021	6	489	Random-effect meta-analysis	– Included studies were of high quality – Zinc supplementation showed greater and significant effect on ADHD total score (SMD = −0.62, −1.24 to −0.002) but not in hyperactivity and inattention scores – The certainty of evidence was rated moderate to very low	Well-designed, large-scale randomized controlled trials are needed to establish the effect of zinc supplementation on ADHD	High (9/9)
Granero et al. [200]	Determine the effect of iron and zinc in the treatment of ADHD	Zinc, Iron, Treatment	January 2000–July 2021	9	Not given	Narrative synthesis	– Included studies were of moderate quality – The specific role of dietary nutrients with zinc and iron still seems controversial for the treatment of ADHD	Further investigations including large sample size and high quality clinical trials are needed to confirm the effects of these diet interventions	Moderate (6/9)
Gan et al. [201]	Quantify the effect of vitamin D supplementation on ADHD	Vitamin D, Supplementation	Not clear	4	256	Random-effect meta-analysis	– Most of the included studies were of moderate quality – Vitamin D supplementation showed a small, but a significant effect on ADHD symptoms – Moderate heterogeneity (*I* ^2^ > 50%) was observed across analysis	More high quality clinical trials are needed to make a definitive conclusion	High (9/9)
Bruton et al. [202]	Assess efficacy of phosphatidylserine for symptoms of ADHD	Integrative medicine	Not clear	4 (3 for meta-analysis)	344	Random-effect meta-analysis	Due to low quality of evidence no firm conclusion can be made about the efficacy of phosphatidylserine for treatment of ADHD	Additional rigorous research is warranted to investigate phosphatidylserine as a low-cost and likely low-risk intervention for children with ADHD	High (8/9)
Anheyer et al. [203]	Evaluate the impact of herbal medicines on ADHD	Herbal medicines, Nonpharmacological treatment, Children and adolescents	Database inception – July 2016	9	464	Narrative synthesis	Small number of RCTs restricted from making any firm conclusion about the efficacy of different herbal medicines on treating ADHD symptoms	Methodologically rigorous studies are needed	Moderate (7/9)
Zwi et al. [204]	Assess the effectiveness of parent training interventions for ADHD in children and adolescents	Parent training interventions, Nonpharmacological interventions, Children and adolescents	Database inception-September 2010	5	284	Narrative synthesis and random-effect meta-analysis	Small sample size and low quality of included studies restricted from making any firm conclusion about the efficacy of parental training for ADHD	Further well-designed, randomized controlled trials within this population are needed and should be reported clearly following the principles set out in the CONSORT 2010 Statement	High (9/9)
Montoya et al. [205]	Evaluate the impact of psychoeducation interventions for parents and teachers in ADHD children	Psychoeducation, Parents, Teachers	Database inception – 2010	7	2,034	Narrative synthesis	– Few studies showed positive results for improvement in patients’ behavior, parents’ and children’s satisfaction, child’s knowledge about ADHD, including their positive opinion and adherence to medical treatment – However, availability of limited data, low sample size, and variability in the concept of psychoeducation restricted from making any firm conclusion about this intervention	Future studies should include adequate sample size and use the clear concept of psychoeducation	High (8/9)
Storebø et al. [206]	Evaluate the effect of social skills training on ADHD	Social skills training, Nonpharmacological treatment, Children and adolescents	Database inception – July 2018	25	2,690	Narrative synthesis and fixed and random-effect meta-analysis	Low quality of included studies, lack of clinical significance, high heterogeneity, and low certainty restricted from making any firm conclusion about the efficacy of social skills training in reducing ADHD symptoms	Methodologically rigorous clinical trials with a large sample size are needed	High (9/9)
Richardson et al. [207]	Evaluate the effectiveness of school-based nonpharmacological interventions on ADHD	Nonpharmacological interventions, School-based settings, Children and adolescents	1980 February–August 2013	54	Not given	Narrative synthesis	Low quality of included studies and substantial heterogeneity in effect size restricted from making any definitive conclusion about the efficacy of school-based nonpharmacological interventions on ADHD	The lack of standardized tools and outcome measures for assessing ADHD behavior, as well as the lack of studies assessing possible moderators in combinations with interventions, present opportunities for future research	High (8/9)
Helmer et al. [208]	Evaluate the effectiveness of EAS in children with ADHD	Equine-assisted services and therapies, Environment	Database inception-December 2020	12	184	Narrative synthesis	– Included studies were of moderate quality – EAS may be beneficial in promoting the physiological functions of body systems for children with ADHD	Further controlled studies, with larger sample sizes, are needed to understand the specific effects of different EAS on the core symptoms and consequence of ADHD	High (8/9)
Perez -Gomez et al. [209]	Synthesize the effectiveness of equine assisted activities in children with ADHD	Animal- assisted therapy, Equine-assisted therapy, Nonpharmacological interventions	Database inception to November 2019	9	181	Narrative synthesis	Low quality of included studies restricted from making any firm conclusion about the efficacy of equine assisted activities in children with ADHD	Studies with robust methodology are needed	Moderate (6/9)
Wilkes-Gillan et al. [210]	Synthesize the evidence for video-modeling interventions for individuals with ADHD	Video-modeling	Not given	11	1–35 participants	Narrative synthesis	– Included studies were of high quality – Small number of clinical trials and sample size restricts from making any firm conclusion about the effectiveness of video-modeling interventions	Future studies need to lower the risk of bias and use larger sample sizes before the efficacy of video-modeling interventions can be fully investigated	Moderate (7/9)
Lee et al. [211]	Determine the effect of acupuncture on ADHD	Acupuncture, Nonpharmacological treatment, Children and adolescents	Database inception- October 2010	3	Not given	Random-effect meta-analysis	Small number of RCTs and low quality of included studies restricted from making any firm conclusion about the efficacy of acupuncture on treating ADHD symptoms	Future studies should include robust methodology to avoid or lower the risk of bias	High (8/9)
Cerrillo-Urbina et al. [212]	Examine the evidence for the effectiveness of exercise interventions on ADHD	Exercise, Children and adolescents	Database inception- November 2014	8	249	Random-effect meta-analysis	Small number of RCTs, low quality of included studies and heterogeneity in the outcome measures restricted from making any firm conclusion about the efficacy of physical exercise like aerobics, yoga on treating ADHD symptoms	Additional studies are needed to obtain consistent clinically relevant conclusions	High (9/9)
**Adults**									
Lopez -Pinar et al. [213]	Assess the long-term efficacy of psychosocial treatments (CBT, dialectical-behavior therapy, mindfulness-based cognitive therapy) for ADHD	Adult ADHD treatments, Psychosocial treatments	Database inception-September 2017	12	1, 073	Random-effect meta-analysis and meta-regression	– Included studies were of low quality – Compared to the control group, the treatment group as a whole reported better improvement in terms of total ADHD symptoms, inattention, and hyperactivity/impulsivity, as well as for clinical global assessment and global functioning for at least 12 months after ended treatment – Significant heterogeneity (*I* ^2^ > 75%) was observed across analysis	Further research should examine whether psychosocial interventions works on different ADHD symptom dimensions or not	High (9/9)
Lopez et al. [214]	Examine the effectiveness of cognitive-behavioral-based therapy for ADHD	Cognitive-behavioral therapy, Adults	Database inception – June 2017	14	700	Random-effect meta-analysis	Imprecision (i.e., inaccurate results), inconsistency (i.e., results differ across trials) and methodological limitations restricted from making any firm conclusion about the efficacy of CBT in treating ADHD symptoms	Multi-center long-term studies are needed to determine the effectiveness of CBT for adults with ADHD. Such studies should also include cost-effectiveness analyses	High(9/9)
Lopez-Pinar et al. [215]	Determine the efficacy of psychotherapies on comorbid internalizing symptoms in ADHD adults	Nonpharmaceutical intervention, Internalizing comorbidity, Adult ADHD treatment	Database inception – October 2018	35 in quantitative analysis and 6 in qualitative synthesis	1,389	Random-effect meta-analysis	– Included studies were of low quality – CBT was efficacious in improving comorbid anxiety and depression symptoms, quality of life, and emotional dysregulation, particularly at 3–12 months follow-up period – Moderate to significant heterogeneity (*I* ^2^ : 60–82%) was observed across analysis	Findings should be further strengthened using a large sample and comparison group, address issues of randomization and attrition bias, perform intention to treat analysis	High(9/9)
Poissant et al. [216]	Examine the effectiveness of MBI in adults with ADHD	Mindfulness-based interventions, Behavioral and cognitive impact, Adults	Database inception – July 2018	13	753	Narrative synthesis	– Included studies were of low quality – Besides improving ADHD symptoms at 3-to – 6-month posttreatment follow-up, mindfulness meditation improved executive function, emotional regulation, and post-intervention cognitive task performance	Future research should address methodological issues like lack of randomization and control groups, sample size variations, and duration of intervention	Moderate (7/9)
Bruce et al. [217]	Assess and identify the most effective behavioral interventions that improves driving outcomes in novice drivers with ADHD	Behavioral interventions, Driving, Hazard perception	Database inception – 2013	13	Not given	Narrative synthesis	Training led to significant enhancement in driving performance in two studies, but methodological issues compromised its validity. Situation awareness training, like commentary driving, was found to be useful	Long-term effect of nonpharmacological interventions (situation awareness, and particularly hazard perception training) in young drivers with ADHD are required	Moderate (6/9)
Salehinejad et al. [218]	Assess the effectiveness of tDCS on neuropsychological deficits in ADHD	Transcranial direct current stimulation, Inhibitory control, Working memory	Database inception – January 2019	10	159	Random-effect meta-analysis	tDCSs appeared as an effective technique for improving inhibitory control and working memory. However, neuropsychological deficits may not correspond directly with clinical symptoms, so the clinical application of this method cannot be yet confirmed	Further research is needed to develop optimal stimulation parameters for improving cognitive function using tDCS and implementing rigorous experimental design across different ADHD subtypes	Moderate (7/9)

Abbreviations: CBT, cognitive behavioral therapy; DBRC, daily behavior report cards; ED, emotional dysregulation; EQS, equine-assisted services; *k*, total number of included studies; MBI, mindfulness-based interventions; NF, neurofeedback; PUFA, polyunsaturated fatty acid; SMD, standardized mean difference; tDCS, transcranial direct current stimulation.

a Total participants included in the systematic review and meta-analysis unless otherwise indicated.

b For findings from meta-analysis, if given effect estimates with 95% CI are presented unless otherwise indicated.

### Pharmacological versus nonpharmacological treatment (n = 7, Table 10)

Two meta-analyses have reported the efficacy of stimulant treatment compared to nonstimulant or other interventions [219, 220]. A network meta-analysis of 190 RCTs found stimulants to be superior compared to nonpharmacological treatment in children and adolescents with ADHD [220]. While medications like extended release-MPH, amphetamine formulations, ATX, and extended release-guanfacine improved ADHD symptoms, psychosocial treatments were beneficial for academic and organizational skills in adolescents [221]. Results from head-to-head trials comparing MPH and neurofeedback were too inconsistent to conclude [222]. Similarly, findings for the efficacy of MPH versus traditional Chinese medicine were suggested by earlier review [223].

A recent systematic review revealed that stimulant treatment appeared to be cost-effective compared to other treatments or no treatment for ADHD in children and adolescents [224]. Similar findings for pharmacotherapy as a whole were suggested by earlier review [225].

**Table 10.** Pharmacological versus nonpharmacological interventions for ADHD

References	Objective	Keywords	Timeframe of database search	*k*	Sample size^a^	Analytical design	Major findings Effect estimates; 95% CI)^b^	Conclusion and comments from authors	Quality assessment
Yang [219]	Meta-regress the effect sizes of stimulant (MPH and LDX), nonstimulant (ATX and alpha-2 agonists), psychosocial therapy (PBT), combination therapy (psychostimulant plus PBT), and alternative/complementary interventions to determine the right treatment for ADHD	Behavioral therapy, Pharmacotherapy, Treatment efficacy	January 1980–July 2018	107	9,883	Random-effect meta-analysis and meta-regression	– Included studies were of high quality – Compared with the stimulant, nonstimulant and alternative or complement intervention were less effective (effect size = −0.38, −0.64 to −0.12) and (effect size = −0.41, −0.79 to −0.04) respectively – However, compared with stimulant, PBT and the combination of stimulant and PBT trials did not differ significantly	These findings will help clinicians, healthcare providers, parents, and caregivers in choosing treatment for ADHD in children and adolescents	Moderate (7/9)
Catala-Lopez et al. [220]	Compare the effectiveness of pharmacological and nonpharmacological treatment for ADHD	Pharmacological treatment, Nonpharmacological treatment, Children and adolescents	Not given	190	26,114	Network meta-analysis	– Included studies were of low quality – Stimulants appeared to be superior to behavioral treatment, cognitive training and nonstimulants, while a combination of stimulants and behavioral therapy had the best combined effect and acceptance rate	RCTs with robust methodology are urgently needed	High (9/9)
Chan et al. [221]	Assess the effectiveness of pharmacological and nonpharmcological treatment for ADHD	Pharmacological treatment, Psychosocial treatment, Adolescents	January 1999–January 2016	17	2,668	Narrative synthesis	Medications were associated with improvements in total ADHD symptoms, while psychosocial treatments led to improvements in functional outcomes like academic and organizational skills	Both pharmacological and nonpharmacological interventional studies should examine how dosage, frequency, intensity and duration affect clinical outcomes, including their effectiveness over longer-term	Moderate (6/9)
Yan et al. [222]	Compare the effectiveness of MPH and NF on ADHD	MPH, Neurofeedback, Children and adolescents	Database inception – August 2018	18	NF: 778 MPH:757	Random-effect meta-analysis	High risk of bias, inconsistency in findings between main analysis and subgroup analysis and nonfunded studies, and mixed findings at the follow-up endpoint restricted from making any firm conclusion about the efficacy of MPH versus NF in reducing ADHD symptoms in children and adolescents	High quality and larger studies are needed to compare the effectiveness	Moderate (7/9)
Lan et al. [223]	Compare the effectiveness of traditional Chinese medicine with MPH in treating ADHD symptoms	Traditional Chinese Medicine, MPH, ADHD	Database inception – June 2008	34	3,167	Random and fixed-effect meta-analysis	– Majority of included studies were of poor quality – No conclusion can be made about the efficacy of Chinese medicine compared to MPH due to lack of high-quality clinical trials	High-quality, multicenter studies are needed to make a firm conclusion	High (8/9)
Dijk et al. [224]	Present full economic evaluations of ADHD treatments	Economic evaluations, Cost-effectiveness	Not given	29	NA	Narrative synthesis	– Almost all studies that compared medication or psychosocial treatment to no treatment, placebo, or care as usual indicated that medication and psychosocial treatment were cost-effective compared to the control group. Stimulant treatment appeared to be cost-effective for the treatment of ADHD in children and adolescents	More cost-effectiveness research of higher quality is warranted to aid in the optimal use of available treatments and resources for individuals with ADHD	Moderate (7/9)
Wu et al. [225]	Assess cost-effectiveness of different pharmacological treatment to better inform payers in the allocation of limited resources	Economic evaluation, ADHD medications,	January 1990–September 2011	13	NA	Narrative synthesis	– Included studies were of moderate quality. – Pharmacological treatment was found to be cost-effective compared to placebo, no medication, or behavioral treatment among children and adolescents. – However, economic evidence comparing different medications was limited and inconclusive	Future research should study the cost effectiveness of medications on ADHD adult population and should assess the long-term cost effectiveness of pharmacological treatment	Moderate (6/9)

Abbreviations: *k*, total number of included studies; LDX, lisdexamphetamine; MPH, methylphenidate; NA, not applicable; NF, neurofeedback; PBT, parental behavioral therapy; RCTs, randomized controlled trials.

a Total participants included in the systematic review and meta-analysis unless otherwise indicated.

b For findings from meta-analysis, if given effect estimates with 95% CI are presented or otherwise specified.

### Patients’ and caregivers’ experience of ADHD beyond symptoms (n = 13, Table 11)

#### Impact on quality of life

Of the two included reviews on health-related quality of life of children and adolescents with ADHD, one found that the parents reported significantly worse health-related quality of life of their children than that reported by the children with ADHD themselves [226], while the other review suggested no such differences [227].

#### Experience with ADHD, pharmacological and nonpharmacological interventions

Individuals with ADHD experience poor academic functioning, pressure to fit in with societal rules and expectations, fear of stigma, and difficulties in being part of the workplace and performing work tasks [228–230]. However, they may also recognize the positive and rewarding aspects of having ADHD [228–230].

Patients with ADHD and their caregivers experience medication as a last resort [231] or as a coping strategy [230]. Some experience that starting medication trigger off an identity crisis [228, 231]. There were reports of concerns about the long-term side effects of medication and financial costs, and the decision of taking medication was based on a process of “pro and con” considerations [228, 231]. Educators, children, and adolescents with ADHD, and their parents felt that school-based nonpharmacological interventions can lead to stigma, but also improve relationships and attitudes [232].

Parenting a child with ADHD was felt as exhausting and emotional journey filled with feelings of guilt, frustration, although “not all bad” [229, 233]. The most commonly used coping strategy of parents seemed to be avoidant-focused coping and was linked to distress and depression [234, 235].

#### Societal and familial barriers to ADHD treatment

Reviews of qualitative studies found perceived stigma as a barrier for acknowledging ADHD by primary care practitioners [236] and for implementing nonpharmacological intervention in schools [237]. There was no sufficient evidence to conclude if poverty moderates psychosocial treatment efficacy for ADHD [238].

**Table 11.** Patients’ and caregivers’ experience of ADHD beyond symptoms

References	Objective	Keywords	Timeframe of database search	*k*	Sample size^a^	Analytical design	Main findings (Effect estimates, 95% CI)^b^	Conclusion and comments from the authors	Quality assessment
**Impact on Quality of Life**									
Lee et al. [226]	Compare children and parents rating for HRQOL for children with ADHD	Health related quality of life Informant agreement	1970–August 2017	8	4,322	Fixed and random-effect meta-analysis, meta-regression	– Included studies were of moderate to high quality – There was an small, but significant difference between parent–child rating of children and adolescents physical HRQOL (Hedge’s *g* = −0.23, −0.33 to −0.13), while moderate difference (*g* = −0.60, −0.71 to −0.48) for psychosocial HRQOL – Significant heterogeneity was observed across analysis	Future meta-analyses may include studies that recruit children with ADHD from the community to reduce selection bias	High (8/9)
Lee et al. [227]	Assess quality of life in children and adolescents with ADHD	Health related quality of life, Parent–Child agreement	1970–2014	9	8,020,867	Random-effect meta-analysis	– Included studies were of moderate to high quality – ADHD severely impaired children’s all three aspects of HRQOL: physical, emotional, and social – For HRQOL of children and adolescents with ADHD, no significant difference was observed between parent’s and children’s rating – Significant heterogeneity (*I* ^2^ > 80%) was observed across analysis	The mechanism through which HRQOL conceptualizations and subscales and family characteristics influence the parent–child agreement on ADHD children’s HRQOL, should be studied	High (8/9)
**Experience with ADHD, pharmacological and nonpharmacological interventions**									
Eccleston et al. [228]	Synthesize adolescents experiences of living with ADHD diagnosis	ADHD diagnosis, Adolescents experiences, Qualitative	Database inception – February 2017	11	166	Narrative synthesis	– Included studies were of high quality – Interpersonal conflict, stigma and rejection lowered adolescents “self-esteem” and “identity” in youths with ADHD, although positive aspects of having ADHD were also recognized	It is important to conduct research on diverse culture, ethnicity, religion and social class, to understand the experiences of different groups of people	High (8/9)
Wong et al. [229]	Synthesize perceptions of ADHD among children with ADHD and their parents	Illness perceptions, Common-sense model, Qualitative	Unclear	101	Not given	Narrative synthesis	The majority of children with ADHD displayed negative emotions, including shame, frustration, despair, and embarrassment, but some felt elated and joyful. Among parents, a much broader range of negative emotions was reported – frustration, stress, depression, guilt, helplessness, anger, loneliness; however, some of them felt relieved to find about the disorder	Research might be able to provide a more comprehensive understanding of perceptions of ADHD and their impact if they examine potential mediators and moderators and potential outcome of interactions between different level of ADHD	High (8/9)
Bjerrum et al. [230]	Synthesize adult experience of living with ADHD	Coping strategies Managing daily living, Qualitative	1995–July 2015	10	159	Narrative synthesis	– Included studies were of high quality – Adults with ADHD recognized the difference between themselves and others and hence struggle to fit into society Although adults with ADHD are creative and innovative, they found it difficult to arrange and complete daily life tasks. However, they find it to be rewarding and aimed to achieve a healthy balance in life through coping strategies	Research on the experience of ADHD adults from non-western countries are required	High (9/9)
Rashid et al. [231]	Summarize medication-taking experiences of ADHD patients and caregivers	Medications, Adherence, Qualitative	1987–October 2015	31	Not given	Narrative synthesis	– Included studies were of moderate quality – As children and adolescents with ADHD transition into adulthood, they become more autonomous and self-directed toward all aspects of medications, and their decision-making process is framed by “trade-offs” where the advantage and disadvantages of medications are considered	Future research should focus on family dynamics, including both siblings and parents, and how media portrayals impact ADHD perceptions and treatment	Moderate (7/9)
Moore et al. [232]	Synthesize attitude and experience toward school-based nonpharmacological interventions for ADHD	Nonpharmacological interventions, School, Qualitative	Unclear	33	31	Narrative synthesis	– Included studies were of high quality – Findings were categorized into four interrelated themes: “individualizing interventions”; “structure of interventions”; “barriers to effectiveness” and; “perceived moderators and impact of interventions.” In addition to ADHD symptoms, school-based interventions should consider the broader school context	Future research should also study how school-based interventions influence attitudes toward ADHD and peer-relationships	High (8/9)
Laugesen et al. [233]	Summarize the experience of parents of ADHD children	Child, Parents, Qualitative	Databases from their inception – April 2015	21	Not given	Narrative synthesis	– Included studies were of high quality – Having a child with ADHD can be an overwhelming and stressful experience, and one can feel guilty, hopeless, and frustrated. Parents were stigmatized and blamed. Their personal and family routines became chaotic and they had to fight to get professional support for their children’s at both school and health services. However, they felt that despite these challenges, living with ADHD child was not all bad	Further research needs to explore how health professionals can support families and how future interventions can improve the competencies of health professionals and assist the families	High (8/9)
Martin et al. [234]	Assess the association between ADHD child sleep problems, parenting stress and parent mental health	Sleep problem, Parenting stress, Mental Health	Database inception – May 30, 2019	4	Not given	Narrative synthesis	Sleep problems among children with ADHD led to higher parenting stress and negatively impacted parental mental health	Future research should control for potential confounders like child’s age, comorbidity to assess if the association is causal	Moderate (7/9)
Craig et al. [235]	Determine the coping strategies used by parents of ADHD children	Coping, Stress, Qualitative	Database inception – July 2018	14	3,024	Narrative synthesis	Parents of children with ADHD often resort to an “avoidant-focused” coping strategy that is basically comprised of cognitive and behavioral activities aimed at avoiding direct contact with stressful demands, and is associated with distress and depression. In comparison to mothers of typically developing children, those with ADHD tended to seek more support and employ indirect methods	Future studies should study coping strategies in parents according to different subtypes of ADHD in children and also with other mental disorder	Moderate (7/9)
**Societal and familial barriers to ADHD treatment**									
French et al. [236]	To identify barriers and facilitators in understanding ADHD in primary care	Primary care, Qualitative	Database inception – January 2018	46	Health professionals: 15,314 Parents: 134	Narrative synthesis	– Majority of the included studies were of high quality – Findings suggest that “need for education,” “misconception and stigma,” “constraint with recognition,” “management and treatment,” and “multidisciplinary approach,” are the main factors influencing the acknowledgment of ADHD by primary care practitioners. A considerable need for improved education about ADHD among primary care practitioners was also found – Significant heterogeneity were found across studies	For more specific solutions, future research should study scarcity of resources, misperceptions, and multidisciplinary approaches in health care settings	High (9/9)
Gwernan-Jones et al. [237]	Explore the influence of school-context on ADHD symptoms	School-stigma, Attributions, Qualitative	1980 – March 2013	34	Not given	Narrative synthesis	– Majority of the included studies were of high quality – Teachers and students may be blind to the role that schools play in ADHD symptoms despite the fact that the potential for stigma-based discrimination in schools is known. This is because stigma-based discrimination criteria are implicit and seem normal and appropriate to those who belong to the group. The stigmatized individuals may experience emotional pain as a result of this lack of understanding, which may worsen the symptoms of ADHD. The implementation of school-based treatments may also be aided by awareness of these potential implications of the school context	Future qualitative research could examine modifications to the school environment, routines, and expectations, including support for connections between students and their teachers and peers	Moderate (7/9)
Ogle et al. [238]	Assess efficacy of psychosocial treatment among ADHD children living in poverty	Poverty, Psychological interventions	Unclear	5	461	Narrative synthesis	Mixed evidence exists about the impact of poverty on psychosocial treatment for children with ADHD	Future research with more accuracy, precision, and quality in the reporting of income data are warranted	High (8/9)

Abbreviations: HRQOL, health related quality of life; *k*, total number of included studies.

a Total participants included in the systematic review and meta-analysis unless otherwise indicated.

b For findings from meta-analysis, if given effect estimates with 95% CI are presented unless otherwise indicated.

## Discussion

To the best of our knowledge, this is the first systematic meta-review that summarizes the main findings of 231 existing systematic reviews and meta-analyses on ADHD of moderate to high quality.

Different from earlier narrative reviews of evidence-based conclusions about ADHD [239–241], we have pre-registered the protocol, adhered to the PRISMA and JBI guidelines to perform a systematic quality assessment of the included reviews. However, our review has some limitations that should be considered. Importantly, this meta-review is not an overview of all published literature on ADHD but limited to academic publications on ADHD covered by systematic reviews and meta-analyses of moderate to high quality, indexed in the five most relevant databases or found in reference lists. Grey literature publications were not covered in our search. Secondly, our inclusion criteria and threshold for quality assessment were quite strict, thus excluding some of the pertinent systematic reviews on topics including genetics [242–249], neurobiology [250, 251], prevalence including young adults [252–254], comorbidities like dyslexia [255, 256], speech disorder [257], borderline personality disorder [258] and nonpharmacological interventions in adults [259].

Nevertheless, this meta-review will aid both researchers and clinicians to get an update on the main conclusions from ADHD research. Importantly our review may also be used as a bird’s eye view by identifying areas where there is sufficient evidence, insufficient evidence, and systematic weaknesses in reviews in various fields of ADHD. In the following, we will summarize some of our key findings with emphasis on weaknesses in the literature of systematic reviews on ADHD.

### Prevalence of ADHD

According to included meta-analyses, the prevalence of ADHD is 7.2% in children [12] and 2.5% in adults [18]. However, there is considerable variation in the reported prevalence between the original studies included in these reviews. This strong variation is not fully understood, although probably due to other factors than true phenotype differences within or between populations studied. More plausible explanations for the variation in prevalence might include: (i) There are differences in research design, applied diagnostic instruments, and source of information which may cause bias [252]. (ii) There may be variation in provider-preference, that is clinician-dependent variation in assessment and diagnostic decisions [260]. This variation may be caused by variation in clinicians’ attitudes toward ADHD diagnosis and medications, for example, from a liberal to a restrictive position, even within uniform health systems [261]. (iii) There may be variation in supply [262–264] and demand [265] of health services, which in turn ultimately will affect variation in rates of diagnosed ADHD. (iv) Finally, the rate of diagnosed ADHD has increased over the last decades, both in children [12, 17, 266] and adults [18]. This may reflect a previous under-recognition of ADHD or increasing over-diagnosis [17]. Hence, this has left the field of ADHD research with the question of how certain are we of the prevalence of ADHD? The uncertainty is not merely a question of narrowing the confidence interval in meta-analyses by including more studies. More sophisticated reviews are needed, addressing the issues of various forms of bias in original studies.

### Risk factors for ADHD: Correlations versus causality

There is evidence of a whole range of ADHD “risk factors,” including for example biological [20], maternal [21–28, 30, 31, 35], environmental [39, 41, 46], social [47], and nutritional factors [49, 50]. However, this literature is mostly based on research designs precluding conclusions on causality due to problems with confounding, and reverse causality. Adjustment for confounders usually makes a difference in such correlational studies, indicating residual confounding due to a lack of information on potential confounders or the reliability of measured confounders. As an example, maternal smoking has, and still is, consistently associated with offspring ADHD in classical epidemiological studies. However, later years’ research, combining different approaches, has shown that this association is not causal, but mainly due to genetic confounding [267–269]. Hence, we need more original studies that integrate results from different methodological approaches [270], allowing for causal inference.

### Long-term prognosis of ADHD

The included reviews indicate a rather bleak prognosis over months and years of follow-up for young patients with ADHD in terms of criminal behavior [75, 76], school dropout [61, 62], vocational challenges [61, 62], injuries [68–70], comorbidities [80, 83, 84], and welfare dependency [61]. However, ADHD is correlated with a whole range of “risk factors” which themselves may be causally linked to challenging life trajectories. Studies of prognosis in ADHD may thus be confounded, and overestimate the negative prognosis of ADHD.

Since ADHD is a very heterogeneous condition, studies focusing more on predictors for different prognostic trajectories would be more informative and fruitful both for increasing our understanding of underlying mechanisms, targeting treatment, and preventing negative outcomes. Future systematic reviews should specifically address issues of publication bias, confounding, and residual confounding, and aim to highlight studies allowing for causal inference.

### Pharmacological treatment of ADHD

There is strong evidence for ADHD symptom reduction during weeks or even months of stimulant use [113, 115, 121, 131] and also some evidence for nonstimulant medication [121, 137] in both children, adolescents, and adults. There is, however, an interesting controversy on how solid the trial evidence on ADHD medication efficacy is. Several reviews on both children and adults, and for both stimulant and nonstimulant pharmacotherapy, conclude with caution as to conclusions on efficacy [123, 124, 128, 130, 135, 139, 157, 158].

The follow-up time in trials is generally in terms of weeks, and the focus has mainly been on symptom scales rather than real-life outcomes [113, 115]. We need systematic review evidence on the efficacy of ADHD pharmacotherapy to mitigate the negative prognosis in ADHD. Can we prevent criminal behavior, school dropout, poor academic performance, vocational challenges, accidents, suicide, comorbidities, and welfare dependency, during years of follow-up, with pharmacological therapies? This is perhaps the most pressing question in the ADHD community, and it is yet to be answered. It should be noted that RCTs due to ethical and practical reasons, and conventional epidemiological studies due to the issue of residual confounding, have not been able to address this evidence gap [260]. We need evidence for efficacy based on large-scale, population-based studies with research designs allowing for causal inference as to the efficacy of ADHD medication in mitigating the rather bleak life trajectory in ADHD. These studies need to reach beyond symptom relief over weeks follow-up, but rather address outcomes regarding life-trajectories with over several years follow-up as to criminal behavior [75, 76], school dropout [61, 62], vocational challenges [61, 62], injuries [68–70], comorbidities [80, 83, 84], and welfare dependency [61, 271].

### Nonpharmacological treatment of ADHD

The evidence on the efficacy of nonpharmacological treatment for ADHD is more mixed than that of pharmacotherapies. Mixed evidence exists for almost all type of nonpharmacological interventions: behavioral intervention for children and adolescents [175, 177, 193] and for adults [213, 214], parental training [175, 176, 204, 205], dietary interventions [194–201], mindfulness [184, 187–189], and other interventions. One reason may be that these interventions are more complex both to deliver and study, for example, therapies being less standardized than drugs, and also that it is more difficult to design blinded and “placebo”-controlled conditions for these more complex intervention. Despite this mixed evidence, ADHD treatments beyond pharmacotherapies are commonly administered and recommended in clinical guidelines [7].

## Conclusion

In this meta-review, we found a large number of reviews that have reasonably well elucidated the evidence for different topics on ADHD. However, when summarizing the findings from the included reviews we still see some important knowledge gaps, for example on prevalence and risk factors. The most pressing knowledge gap is probably that of the efficacy of current ADHD treatments in mitigating the rather bleak life trajectory in ADHD. Hence, future systematic reviews and meta-analyses should address the identified knowledge gaps related to ADHD. To some extent, the lack of systematic review and meta-analysis evidence reflects lack of relevant original studies on ADHD.

## Supporting information

Chaulagain et al. supplementary material Chaulagain et al. supplementary material

## Supplementary material

The supplementary material for this article can be found at http://doi.org/10.1192/j.eurpsy.2023.2451.
