# Supplementary material for: A systematic meta-review of systematic reviews on attention deficit hyperactivity disorder
Source: Eur Psychiatry. 2023 Nov 17;66(1):e90. doi: 10.1192/j.eurpsy.2023.2451 (PMC10755583; doi:10.1192/j.eurpsy.2023.2451)
Supplement: Chaulagain et al. supplementary material [file S0924933823024513sup001.doc]

Supplementary 1

**A systematic meta-review of systematic reviews on
Attention Deficit Hyperactivity Disorder (ADHD)**

**Supplementary material**

**Methods**

Search strategy

Manual for Quality assessment and Data Extraction

**Table S1.** PRISMA Checklist

**Table S2.** List of excluded articles with reasons

***Search strategy***

AC conducted the search using the keywords for ADHD, systematic review, meta-analysis.

Example of full electronic search strategy for (PubMed):

(((((Attention Deficit Hyperactivity Disorder[Title]) OR Attention-Deficit Hyperactivity Disorder[Title]) OR ADHD[Title]) OR Hyperkinetic Disorder[Title])) AND ((Systematic review*[Title]) OR Meta-analysis*[Title]) Filters: Publication date from 2010/01/01 to 2019/12/31

Updated search

(((((Attention Deficit Hyperactivity Disorder[Title]) OR Attention-Deficit Hyperactivity Disorder[Title]) OR ADHD[Title]) OR Hyperkinetic Disorder[Title])) AND ((Systematic review*[Title]) OR Meta-analysis*[Title]) Filters: Publication date from 2019/12/4 to 2021/02/14

Updated search

(((((Attention Deficit Hyperactivity Disorder[Title]) OR Attention-Deficit Hyperactivity Disorder[Title]) OR ADHD[Title]) OR Hyperkinetic Disorder[Title])) AND ((Systematic review*[Title]) OR Meta-analysis*[Title]) Filters: Publication date from 2021/02/15 to 2021/12/31

Updated search

(((((Attention Deficit Hyperactivity Disorder[Title]) OR Attention-Deficit Hyperactivity Disorder[Title]) OR ADHD[Title]) OR Hyperkinetic Disorder[Title])) AND ((Systematic review*[Title]) OR Meta-analysis*[Title]) Filters: Database inception to 2009/12/31

Manual for quality assessment and data extraction

# Quality assessment

*We adopted the JBI guidelines and made some amendments after piloting the guideline. Among the 11 items listed in the guideline we used the first nine items that are given below. Some changes we made on the criteria for scoring are highlighted.

## 1. Is the review question clearly and explicitly stated?

No (High risk of bias)/Yes (Low risk of bias)/Unclear (Not applicable “NA”)

The review question is an essential step in the systematic review process. A well-articulated question defines the scope of the review and aids in the development of the search strategy to locate the relevant evidence. An explicitly stated question, formulated around its PICO (Population, Intervention, Comparator, Outcome) elements aids both the review team in conducting the review and the reader in determining if their review has achieved its objectives. Ideally the review question should be articulated in a published protocol; however this will not always be the case with many reviews that are located.

## 2. Were the inclusion criteria appropriate for the review question?

No (High)/Yes (Low)/Unclear (Not applicable “NA”)

The inclusion criteria should be identifiable from and match the review question. The necessary elements of the PICO should be explicit and clearly defined. The inclusion criteria should be detailed and the included reviews should clearly be eligible when matched against the stated inclusion criteria. Appraisers of meta-analyses will find that inclusion criteria may encompass criteria around the ability to conduct statistical analyses which would not be the norm for a systematic review. The types of included studies should be relevant to the review question, for example, an Umbrella Review aiming to summarize a range of effective non-pharmacological interventions for aggressive behaviors amongst elderly patients with dementia will limit itself to including systematic reviews and meta-analyses that synthesize quantitative studies assessing the various interventions; qualitative or economic reviews would not be included.

(if language criteria mentioned, this should rather be covered by #3)

Relative to #1; should match in relation to how specific/global it is for instance

## 3. Was the search strategy appropriate?

No (High)/Yes (Low)/Unclear (Not applicable “NA”)

A systematic review should provide evidence of the search strategy that has been used to locate the evidence. This may be found in the methods section of the review report in some cases, or as an appendix that may be provided as supplementary information to the review publication. A systematic review should present a clear search strategy that addresses each of the identifiable PICO components of the review question. Some reviews may also provide a description of the approach to searching and how the terms that were ultimately used were derived, though due to limits on word counts in journals this may be more the norm in online only publications. There should be evidence of logical and relevant keywords and terms and also evidence that Subject Headings and Indexing terms have been used in the conduct of the search. Limits on the search and their potential impact should also be considered; for example, if a date limit was used, was this appropriate and/or justified? If only English language studies were included, will the language bias have an impact on the review? The response to these considerations will depend, in part, on the review question.

**exclusion criteria** relevant? (e.g. language or date restrictions)

Excluding by language criteria in search is never acceptable; 🡪 **HIGH**

Excluding by language at a later time in the process may be acceptable depending on the topic being reviewed (usually **LOW**)

Date restriction- justification given -**LOW**, if not **UNCLEAR** or **HIGH** depending on the topic being reviewed

## 4. Were the sources and resources used to search for studies adequate?

No (High)/Yes (Low)/Unclear (Not applicable “NA”)

A systematic review should attempt to identify “all” the available evidence and as such there should be evidence of a comprehensive search strategy. Multiple electronic databases should be searched including major bibliographic citation databases such as MEDLINE and CINAHL. Ideally, other databases that are relevant to the review question should also be searched, for example, a systematic review with a question about a physical therapy intervention should also look to search the PEDro database, whilst a review focusing on an educational intervention should also search the ERIC. Reviews of effectiveness should aim to search trial registries. A comprehensive search is the ideal way to minimize publication bias. As a result, a well conducted systematic review should also attempt to search for gray literature, or “unpublished” studies; this may involve searching websites relevant to the review question or thesis repositories.

## 5. Were the criteria for appraising studies appropriate?

No (High)/Yes (Low)/Unclear (Not applicable “NA”)

The systematic review should present a clear statement that critical appraisal was conducted and provide details of the items that were used to assess the included studies. This may be presented in “Methods of the review”, as an appendix of supplementary information, or as a reference to a source that can be located. The tools or instruments used should be appropriate for the review question asked and the type of research conducted. For example, a systematic review of effectiveness should present a tool or instrument that addresses aspects of validity for experimental studies and randomized controlled trials such as randomization and blinding – if the review includes observational research to answer the same question, a different tool would be more appropriate. Similarly, a review assessing diagnostic test accuracy may refer to the recognized QUADAS tool.

## 6. Was critical appraisal conducted by two or more reviewers independently?

No (High)/Yes (Low)/Unclear (Not applicable “NA”)

Critical appraisal or some similar assessment of the quality of the literature included in a systematic review is essential. A key characteristic to minimize bias or systematic error in the conduct of a systematic review is to have the critical appraisal of the included studies completed by members of the review team independently and in duplicate. The systematic review should present a clear statement that critical appraisal was conducted by at least two reviewers working independently from each other and conferring where necessary to reach a decision regarding study quality and eligibility on the basis of quality.

More than one reviewer involved, even if not 100 % at any stage of review = LOW

## 7. Were there methods to minimize errors in data extraction?

No (High)/Yes (Low)/Unclear (Not applicable “NA”)

Efforts made by review authors during data extraction can also minimize bias or systematic errors in the conduct of a systematic review. Strategies to minimize bias may include conducting all data extraction in duplicate and independently, using specific tools or instruments to guide data extraction and some evidence of piloting or training around their use.

Two reviewers = acceptable (LOW)

When DE is done by one and checked by others: Low (As there has been some measures to minimize errors)

Used extraction checklist/tool = acceptable (LOW)

## 8. Were the methods used to combine studies appropriate?

No (High)/Yes (Low)/Unclear (Not applicable “NA”)

A synthesis of the evidence is a key feature of a systematic review. The synthesis that is presented should be appropriate for the review question and the stated type of systematic review and evidence it refers to. If a meta-analysis has been conducted this needs to be reviewed carefully. Was it appropriate to combine the studies? Have the reviewers assessed heterogeneity statistically and provided some explanation for heterogeneity that may be present? Often, where heterogeneous studies are included in the systematic review, narrative synthesis will be an appropriate method for presenting the results of multiple studies. If a qualitative review, are the methods that have been used to synthesize findings congruent with the stated methodology of the review? Is there adequate descriptive and explanatory information to support the final synthesized findings that have been constructed from the findings sourced from the original research?

## 9. Was the likelihood of publication bias assessed?

No (High)/Yes (Low)/Unclear (Not applicable “NA”)

As mentioned, a comprehensive search strategy is the best means by which a review author may alleviate the impact of publication bias on the results of the review. Reviews may also present statistical tests such as Egger’s test or funnel plots to also assess the potential presence of publication bias and its potential impact on the results of the review.

## 10. Overall appraisal:

Exclude (High)/Include (Low)/Seek further info (Unclear)

Exclude / include in review, i.e. go on to data extraction. Seek further info = discuss with other reviewer.

To include: at least total **6 items** = LOW

Based on this guideline, two reviewers (AC, IL) independently assessed the quality of 31% of the systematic reviews. However, for those articles where QA was not done in duplicate: If there were more than 3 items that were not LOW or if reviewer was uncertain about rating, then the article was discussed with other reviewer.

# Data extraction

1. Covidence article # “ ”
2. Intervention/phenomena of interest (“Thematic category code” )
3. Objectives/aim (from abstract) -
4. Participants (Total number and if not given categories: <500 500-2000 2000-10 000 > 10 000 )
5. Range (years) included in search (“from inception of databases” or similar copy paste text)
6. Number of studies included “..”
7. Types of studies included (“Thematic category code” )
8. Continent(s) of origin of included studies “ / interpretation
9. Appraisal instrument(s) used “
10. Appraisal rating “
11. Method of analysis ”NS” (=narrative synthesis), otherwise “ / interpretation – fixed or random effects model
12. Results/findings (from abstract/result section/ or sometimes from discussion)
13. Heterogeneity

- if meta-analysis: “ (number/general conclusion (e.g. “high”)

- if given that not meta-analysis because of heterogeneity, include “

1. Implications for clinical practice “
2. Implications for future research “
3. Authors’ conclusions “
4. Own comments on added value, topic, quality; personal opinion (interpretation)
5. Key citations “ (optional)

**Differences between protocol and review**

In the protocol ,we mentioned that quality assessment of included systematic reviews and meta-analyses will be carried out by two independent reviewers, using the AMSTAR systematic review critical appraisal tool <https://amstar.ca/Amstar-2.php>. However, as this checklist is for systematic reviews that include randomised and non-randomised studies of health care interventions, and our meta-review include other topics like prevalence, risk factors, etc, along with interventional studies. Therefore, we decided to use JBI guideline for quality assessment. Further, Two reviewers (AC, IL) independently assessed the quality of 31% of the systematic reviews to ensure consistency in the quality assessment rating. There was good agreement in quality assessment, and consequently, the remaining 69% of included studies were scored for quality by one author only. A similar process was followed for data extraction, where three reviewers were involved (AC with IL or ON).

**Table S1. PRISMA Checklist.**

| **PRISMA 2009 Checklist** | | | |
| --- | --- | --- | --- |
| **Section/topic** | **#** | **Checklist item** | **Reported on page** |
| **TITLE** | | |  |
| Title | 1 | Identify the report as a systematic review, meta-analysis, or both. | 1 (identified as “meta review”) |
| **ABSTRACT** | | |  |
| Structured summary | 2 | Provide a structured summary including, as applicable: background; objectives; data sources; study eligibility criteria, participants, and interventions; study appraisal and synthesis methods; results; limitations; conclusions and implications of key findings; systematic review registration number. | 2 |
| **INTRODUCTION** | | |  |
| Rationale | 3 | Describe the rationale for the review in the context of what is already known. | 3 |
| Objectives | 4 | Provide an explicit statement of questions being addressed with reference to participants, interventions, comparisons, outcomes, and study design (PICOS). | 3 |
| **METHODS** | | |  |
| Protocol and registration | 5 | Indicate if a review protocol exists, if and where it can be accessed (e.g., Web address), and, if available, provide registration information including registration number. | 3 |
| Eligibility criteria | 6 | Specify study characteristics (e.g., PICOS, length of follow-up) and report characteristics (e.g., years considered, language, publication status) used as criteria for eligibility, giving rationale. | 3-4 (Table 1) |
| Information sources | 7 | Describe all information sources (e.g., databases with dates of coverage, contact with study authors to identify additional studies) in the search and date last searched. | 3 |
| Search | 8 | Present full electronic search strategy for at least one database, including any limits used, such that it could be repeated. | 3 (Supplement 1) |
| Study selection | 9 | State the process for selecting studies (i.e., screening, eligibility, included in systematic review, and, if applicable, included in the meta-analysis). | 3,4 (Table 1 ) |
| Data collection process | 10 | Describe method of data extraction from reports (e.g., piloted forms, independently, in duplicate) and any processes for obtaining and confirming data from investigators. | 4, Supplement 1) |
| Data items | 11 | List and define all variables for which data were sought (e.g., PICOS, funding sources) and any assumptions and simplifications made. | 4, Supplement 1 |
| Risk of bias in individual studies | 12 | Describe methods used for assessing risk of bias of individual studies (including specification of whether this was done at the study or outcome level), and how this information is to be used in any data synthesis. | 4 , Supplement 1 |
| Summary measures | 13 | State the principal summary measures (e.g., risk ratio, difference in means). | 4-5 |
| Synthesis of results | 14 | Describe the methods of handling data and combining results of studies, if done, including measures of consistency (e.g., I^2^) for each meta-analysis. | 4-5 |
| Risk of bias across studies | 15 | Specify any assessment of risk of bias that may affect the cumulative evidence (e.g., publication bias, selective reporting within studies). | 4 |
| Additional analyses | 16 | Describe methods of additional analyses (e.g., sensitivity or subgroup analyses, meta-regression), if done, indicating which were pre-specified. | NA |
| **RESULTS** | | |  |
| Study selection | 17 | Give numbers of studies screened, assessed for eligibility, and included in the review, with reasons for exclusions at each stage, ideally with a flow diagram. | 5, Figure 1, Supplement 1Table S3 |
| Study characteristics | 18 | For each study, present characteristics for which data were extracted (e.g., study size, PICOS, follow-up period) and provide the citations. | Table 3-11 |
| Risk of bias within studies | 19 | Present data on risk of bias of each study and, if available, any outcome level assessment (see item 12). | Table 3-11 |
| Results of individual studies | 20 | For all outcomes considered (benefits or harms), present, for each study: (a) simple summary data for each intervention group (b) effect estimates and confidence intervals, ideally with a forest plot. | 5-22, Table 2-11, Figure 2 , |
| Synthesis of results | 21 | Present results of each meta-analysis done, including confidence intervals and measures of consistency. | NA |
| Risk of bias across studies | 22 | Present results of any assessment of risk of bias across studies (see Item 15). | Table 3-11, |
| Additional analysis | 23 | Give results of additional analyses, if done (e.g., sensitivity or subgroup analyses, meta-regression [see Item 16]). | NA |
| **DISCUSSION** | | |  |
| Summary of evidence | 24 | Summarize the main findings including the strength of evidence for each main outcome; consider their relevance to key groups (e.g., healthcare providers, users, and policy makers). | 23-27 |
| Limitations | 25 | Discuss limitations at study and outcome level (e.g., risk of bias), and at review-level (e.g., incomplete retrieval of identified research, reporting bias). | 22-26 |
| Conclusions | 26 | Provide a general interpretation of the results in the context of other evidence, and implications for future research. | 23-27 |
| **FUNDING** | | |  |
| Funding | 27 | Describe sources of funding for the systematic review and other support (e.g., supply of data); role of funders for the systematic review. | 28 |

**Table S2 : Quality assessment of overlapping reviews (excluded vs included)**

| Excluded overlapping review | | | Included updated latest review with more articles | | |
| --- | --- | --- | --- | --- | --- |
| Author, Year | Title | Quality assessment | Author, Year | Title | Quality assessment |
| Hanwella et al, 2011 | Comparative efficacy and acceptability of methylphenidate and atomoxetine in treatment of attention deficit hyperactivity disorder in children and adolescents: a meta-analysis | Moderate (6/9) | Rezaei et al, 2016 | Comparative efficacy of methylphenidate and atomoxetine in the treatment of attention deficit hyperactivity disorder in children and adolescents: A systematic review and meta-analysis | Moderate (7/9) (Performed test for publication bias) |
| Wang et al, 2017 | Modafinil for the treatment of attention-deficit/hyperactivity disorder: A meta-analysis | Moderate (6/9)  (Not performed test for publication bias) | Cortese et al, (2018) | Comparative efficacy and tolerability of medications for attention-deficit hyperactivity disorder in children, adolescents, and adults: a systematic review and network meta-analysis | High (8/9 )  (Performed test for publication bias) |
| Maneeton et al, 2011 | Bupropion for adults with attention-deficit hyperactivity disorder: Meta- analysis of randomized, placebo-controlled trials | High (8/9)  (Not performed test for publication) | Verbeeck et al, 2017 | Bupropion for attention deficit hyperactivity disorder (ADHD) in adults  Meta-analysis | High (9/9)  (Not performed test for publication bias) |
| Masarwa et al, 2018 | Prenatal Exposure to Acetaminophen and Risk for Attention Deficit Hyperactivity Disorder and Autistic Spectrum Disorder: A Systematic Review, Meta-Analysis, and Meta-Regression Analysis of Cohort Studies | Low (5/9)  (Not performed test for publication bias) | Gou et al , 2019 | Association of maternal prenatal acetaminophen use with the risk of attention deficit/hyperactivity disorder in offspring: A meta-analysis | Moderate (7/9)  (Performed test for publication bias) |
| Parker et al, 2013 | The long-term outcomes of interventions for the management of attention-deficit hyperactivity disorder in children and adolescents: a systematic review of randomized controlled trials | Moderate (6/9)  (Not performed test for publication bias) | Catala-Lopez et al, 2017 | The pharmacological and non-pharmacological treatment of attention deficit hyperactivity disorder in children and adolescents: A systematic review with network meta-analyses of randomised trials | High (9/9)  (Performed test for publication bias) |
| Kotsi et al, 2019 | Vitamin D levels in children and adolescents with attention-deficit hyperactivity disorder (ADHD): a meta-analysis | Moderate (6/9) (Not performed test for publication bias) | Khoshbakht et al, 2018 | Vitamin D Status and Attention Deficit Hyperactivity Disorder: A Systematic Review and Meta-Analysis of Observational Studies | High (8/9)  (Performed test for publication bias) |
| Jiang et al, 2018 | Antidepressant use during pregnancy and the risk of attention-deficit/hyperactivity disorder in the children: a meta-analysis of cohort studies | Moderate (6/9)  (Not performed test for publication bias) | Man et al, 2018 | Prenatal antidepressant exposure and the risk of attention-deficit hyperactivity disorder in children: A systematic review and meta-analysis | High (8/9)  (Not performed test for publication bias) |
| Wang et al ,2017 | Iron Status in Attention-Deficit/Hyperactivity Disorder: A Systematic Review and Meta-Analysis | High (8/9)  (Performed test for publication bias) | Tseng et al, 2018 | Peripheral iron levels in children with attention-deficit hyperactivity disorder: a systematic review and meta-analysis | High (8/9)  (Performed test for publication bias) |
| He et al ,2017 | Maternal Smoking During Pregnancy and ADHD: Results From a Systematic Review and Meta-Analysis of Prospective Cohort Studies | Moderate (6/9)  (Not performed test for publication bias) | Dong et al, 2018 | Prenatal exposure to maternal smoking during pregnancy and attention-deficit/hyperactivity disorder in offspring: A meta-analysis | High (8/9)  (Performed test for publication bias) |
| Galloway et al , 2017 | Is there a difference between child self-ratings and parent proxy-ratings of the quality of life of children with a diagnosis of attention-deficit hyperactivity disorder (ADHD)? A systematic review of the literature | Low (5/9)  (Not performed test for publication bias) | Lee et al, 2019 | Do parents and children agree on rating a child's HRQOL? A systematic review and Meta-analysis of comparisons between children with attention deficit hyperactivity disorder and children with typical development using the PedsQL(TM) | High (8/9)  (Performed test for publication bias) |
| Sampaio et al, 2021 | The cost-effectiveness of treatments for attention deficit-hyperactivity disorder and autism spectrum disorder in children and adolescents: a systematic review | Low (5/9)  (Not performed test for publication bias) | Dijik et al, 2021 | Cost-Effectiveness and Cost Utility of Treatment of Attention-Deficit/Hyperactivity Disorder: A Systematic Review | Moderate (7/9)  (Not performed test for publication bias) |
| Jensen et al , 2016 | Cognitive behavioural therapy for ADHD in adults: systematic review and meta-analyses | Moderate (6/9)  (Not performed test for publication bias) | Lopez et al, 2018 | Cognitive‐behavioural interventions for attention deficit hyperactivity disorder (ADHD) in adults | High (9/9)  (Performed test for publication bias) |
| Shareghfarid et al, 2019 | Empirically derived dietary patterns and food groups intake in relation with Attention Deficit/Hyperactivity Disorder (ADHD): A systematic review and meta-analysis | Moderate (6/9)  (Performed test for publication bias) | Del-Ponte et al, 2019 | Dietary patterns and attention deficit/hyperactivity disorder (ADHD): A systematic review and meta-analysis | Moderate (6/9)  (Performed test for publication bias yes |
| White et al, 2020 | The effect of equine-assisted therapies on behavioural, psychological and physical symptoms for children with attention deficit/hyperactivity disorder: A systematic review | Moderate (7/9)  (Not performed test for publication bias) | Helmer et al, 2021 | Equine-Assisted Services for Children with Attention-Deficit/Hyperactivity Disorder: A Systematic Review | High (8/9)  (Not performed test for publication bias) |
| Perez-Gomez et al, 2020 | Equine-assisted activities and therapies in children with attention-deficit/hyperactivity disorder: A systematic review | Moderate (6/9)  (Not performed test for publication bias) |

**Table S3 :List of excluded reviews**

| Author, Year | Title | Reason for exclusion |
| --- | --- | --- |
| Storebo et al, 2015 | Methylphenidate for attention-deficit/hyperactivity disorder in children and adolescents: Cochrane systematic review with meta-analyses and trial sequential analyses of randomised clinical trials | Same review with different title |
| Hanwella et al, 2011 | Comparative efficacy and acceptability of methylphenidate and atomoxetine in treatment of attention deficit hyperactivity disorder in children and adolescents: a meta-analysis | Overlapping review |
| Wang et al, 2017 | Modafinil for the treatment of attention-deficit/hyperactivity disorder: A meta-analysis | Overlapping review |
| Maneeton et al, 2011 | Bupropion for adults with attention-deficit hyperactivity disorder: Meta- analysis of randomized, placebo-controlled trials | Overlapping review |
| Masarwa et al, 2018 | Prenatal Exposure to Acetaminophen and Risk for Attention Deficit Hyperactivity Disorder and Autistic Spectrum Disorder: A Systematic Review, Meta-Analysis, and Meta-Regression Analysis of Cohort Studies | Overlapping review |
| Parker et al, 2013 | The long-term outcomes of interventions for the management of attention-deficit hyperactivity disorder in children and adolescents: a systematic review of randomized controlled trials | Overlapping review |
| Kotsi et al, 2019 | Vitamin D levels in children and adolescents with attention-deficit hyperactivity disorder (ADHD): a meta-analysis | Overlapping review |
| Jiang et al, 2018 | Antidepressant use during pregnancy and the risk of attention-deficit/hyperactivity disorder in the children: a meta-analysis of cohort studies | Overlapping review |
| Wang et al ,2017 | Iron Status in Attention-Deficit/Hyperactivity Disorder: A Systematic Review and Meta-Analysis | Overlapping review |
| He et al ,2017 | Maternal Smoking During Pregnancy and ADHD: Results From a Systematic Review and Meta-Analysis of Prospective Cohort Studies | Overlapping review |
| Galloway et al , 2017 | Is there a difference between child self-ratings and parent proxy-ratings of the quality of life of children with a diagnosis of attention-deficit hyperactivity disorder (ADHD)? A systematic review of the literature | Overlapping review |
| Sampaio et al, 2021 | The cost-effectiveness of treatments for attention deficit-hyperactivity disorder and autism spectrum disorder in children and adolescents: a systematic review | Overlapping review |
| Jensen et al , 2016 | Cognitive behavioural therapy for ADHD in adults: systematic review and meta-analyses | Overlapping review |
| Shareghfarid et al, 2020 | Empirically derived dietary patterns and food groups intake in relation with Attention Deficit/Hyperactivity Disorder (ADHD): A systematic review and meta-analysis | Overlapping review |
| White et al, 2020 | The effect of equine-assisted therapies on behavioural, psychological and physical symptoms for children with attention deficit/hyperactivity disorder: A systematic review | Overlapping review |
| Perez-Gomez et al, 2020 | Equine-assisted activities and therapies in children with attention-deficit/hyperactivity disorder: A systematic review | Overlapping review |
| Bjornstad et al, 2005 | Family therapy for attention‐deficit disorder or attention‐deficit/hyperactivity disorder in children and adolescents | Overlapping review |
| Godfrey et al, 2009 | Safety of therapeutic methylphenidate in adults: a systematic review of the evidence | Overlapping review |
| Gonzalez de Dios et al, 2006 | [Methylphenidate in the treatment of attention-deficit/hyperactivity disorder: are we achieving an adequate clinical practice?] | Overlapping review |
| King et al, 2006 | A systematic review and economic model of the effectiveness and cost-effectiveness of methylphenidate, dexamfetamine and atomoxetine for the treatment of attention deficit hyperactivity disorder in children and adolescents | Overlapping review |
| Klassen et al, 1999 | Attention-deficit hyperactivity disorder in children and youth: a quantitative systematic review of the efficacy of different management strategies | Overlapping review |
| Polanczyk et al, 2007 | The worldwide prevalence of ADHD: a systematic review and metaregression analysis | Overlapping review |
| Schab et al, 2004 | Do artificial food colors promote hyperactivity in children with hyperactive syndromes? A meta-analysis of double-blind placebo-controlled trials | Overlapping review |
| Schachter et al, 2001 | How efficacious and safe is short-acting methylphenidate for the treatment of attention-deficit disorder in children and adolescents? A meta-analysis | Overlapping review |
| Mahendiran et al, 2019 | Meta-Analysis of Sex Differences in Social and Communication Function in Children With Autism Spectrum Disorder and Attention-Deficit/Hyperactivity Disorder | Low quality |
| Danborg et al, 2017 | Impaired reproduction after exposure to ADHD drugs: Systematic review of animal studies | Low quality |
| Effatpanah et al, 2019 | Magnesium status and attention deficit hyperactivity disorder (ADHD): A meta-analysis | Low quality |
| Dutra et al, 2016 | Motor cortex excitability in attention-deficit hyperactivity disorder (ADHD): A systematic review and meta-analysis | Low quality |
| deCastroPaiva et al, 2019 | Temporal Reward Discounting in Children with Attention Deficit/Hyperactivity Disorder (ADHD), and Children with Autism Spectrum Disorder (ASD): A Systematic Review | Low quality |
| Donzelli et al, 2019 | Particulate Matter Exposure and Attention-Deficit/Hyperactivity Disorder in Children: A Systematic Review of Epidemiological Studies | Low quality |
| Barkla et al, 2015 | Are there any potentially dangerous pharmacological effects of combining ADHD medication with alcohol and drugs of abuse? A systematic review of the literature | Low quality |
| Holland et al, 2019 | Relative age and ADHD symptoms, diagnosis and medication: a systematic review | Low quality |
| Shaw et al, 2012 | A systematic review and analysis of long-term outcomes in attention deficit hyperactivity disorder: effects of treatment and non-treatment | Low quality |
| Arnold et al, 2015 | Effect of treatment modality on long-term outcomes in attention-deficit/hyperactivity disorder: a systematic review | Low quality |
| Suarez-Manzano et al, 2018 | Acute and chronic effect of physical activity on cognition and behaviour in young people with ADHD: A systematic review of intervention studies | Low quality |
| Muskens et al, 2017 | Medical comorbidities in children and adolescents with autism spectrum disorders and attention deficit hyperactivity disorders: a systematic review | Low quality |
| Karpouzis et al, 2010 | Chiropractic care for paediatric and adolescent Attention-Deficit/Hyperactivity Disorder: A systematic review | Low quality |
| He et al, 2019 | Low blood lead levels and attention-deficit hyperactivity disorder in children: a systematic review and meta-analysis | Low quality |
| Padilha et al, 2018 | Efficacy and safety of drugs for attention deficit hyperactivity disorder in children and adolescents: a network meta-analysis | Low quality |
| Chimiklis et al, 2018 | Yoga, Mindfulness, and Meditation Interventions for Youth with ADHD: Systematic Review and Meta-Analysis | Low quality |
| Fabiano et al, 2021 | Comprehensive Meta-Analysis of Attention-Deficit/Hyperactivity Disorder Psychosocial Treatments Investigated Within Between Group Studies | Low quality |
| Bartoli et al, 2021 | Disentangling the Association between ADHD and Alcohol Use Disorder in Individuals Suffering from Bipolar Disorder: A Systematic Review and Meta-Analysis | Low quality |
| Onandia-Hinchado et al, 2021 | Cognitive characterization of adult attention deficit hyperactivity disorder by domains: a systematic review | Low quality |
| Prentice et al, 2021 | A Systematic Review and Comparison of Neurocognitive Features of Late-Life Attention-Deficit/Hyperactivity Disorder and Dementia With Lewy Bodies | Low quality |
| Sukmajaya et al, 2021 | Systematic review of gut microbiota and attention-deficit hyperactivity disorder (ADHD) | Low quality |
| Salehinejad et al, 2020 | Transcranial Direct Current Stimulation in ADHD: A Systematic Review of Efficacy, Safety, and Protocol-induced Electrical Field Modeling Results | Low quality |
| Özgen et al, 2021 | Treatment of Adolescents with Concurrent Substance Use Disorder and Attention-Deficit/Hyperactivity Disorder: A Systematic Review | Low quality |
| Bundgaard-Nielsen et al, 2020 | Gut microbiota profiles of autism spectrum disorder and attention deficit/hyperactivity disorder: A systematic literature review | Low quality |
| Cosmo et al, 2020 | A systematic review of transcranial direct current stimulation effects in attention-deficit/hyperactivity disorder | Low quality |
| Veloso et al, 2020 | Effectiveness of Cognitive Training for School-Aged Children and Adolescents With Attention Deficit/Hyperactivity Disorder: A Systematic Review | Low quality |
| Frazier et al, 2004 | Meta-analysis of intellectual and neuropsychological test performance in attention-deficit/hyperactivity disorder | Low quality |
| Riccio et al, 2004 | The status of empirical support for treatments of attention deficits | Low quality |
| Verbeeck et al, 2009 | Antidepressants in the treatment of adult attention-deficit hyperactivity disorder: a systematic review | Low quality |
| Deault, 2010 | A systematic review of parenting in relation to the development of comorbidities and functional impairments in children with attention-deficit/hyperactivity disorder (ADHD) | Low quality |
| Lv et al, 2012 | Effectiveness and safety of methylphenidate and atomoxetine for attention deficit hyperactivity disorder: a systematic review | No clear description of article selection process |
| DenHeijer et al, 2017 | Sweat it out? The effects of physical exercise on cognition and behavior in children and adults with ADHD: a systematic literature review | No clear description of article selection process |
| Li et al, 2011 | Acupuncture for Attention Deficit Hyperactivity Disorder (ADHD) in children and adolescents | No clear description of article selection process |
| Krisanaprakornkit et al, 2010 | Meditation therapies for attention‐deficit/hyperactivity disorder (ADHD) | No clear description of article selection process |
| Shooshtary et al, 2010 | The prevalence of Attention Deficit Hyperactivity Disorder in Iran: A systematic review | No clear description of article selection process |
| Sedky et al, 2014 | Attention deficit hyperactivity disorder and sleep disordered breathing in pediatric populations: a meta-analysis | No clear description of article selection process |
| Moriyama et al, 2013 | Psychopharmacology and psychotherapy for the treatment of adults with ADHD-a systematic review of available meta-analyses | No clear description of article selection process |
| Stuckelman et al, 2017 | Risk of Irritability With Psychostimulant Treatment in Children With ADHD: A Meta-Analysis | Not performed quality assessment |
| Cook et al, 2017 | Managing attention deficit hyperactivity disorder in adults using illicit psychostimulants: A systematic review | No involvement of two independent reviewer |
| Clayton et al, 2018 | Quantifying the Relationship between Attention-Deficit/Hyperactivity Disorder and Experiences of Child Maltreatment: A Meta-Analysis | Not performed quality assessment |
| Buitelaar et al, 2000 | [Attention deficit hyperactivity disorder (ADHD): etiology, diagnosis and treatment] | Not in English |
| Davids et al, 2004 | [Atomoxetine for the treatment of attention-deficit/hyperactivity disorder] | Not in English |
| Schöffski et al, 2008 | [Overall burden to society caused by hyperkinetic syndrome (HKS) and attention deficit hyperactivity disorder (ADHD)] | Not in English |
| Simon et al, 2007 | [Detailed review of epidemiologic studies on adult Attention Deficit/Hyperactivity Disorder (ADHD)] | Not in English |
| Wohl et al, 2005 | [Meta-analysis of candidate genes in attention-deficit hyperactivity disorder] | Not in English |
| Lv et al, 2011 | [Effectiveness and safety of methylphenidate and atomoxetine for attention deficit hyperactivity disorder: a systematic review] | Not in English |
| Ni XQ et al, 2015 | A Meta-analysis on Acupuncture Treatment of Attention Deficit/Hyperactivity Disorder | Not in English |
| Rodrigo-Ruiz et al, 2017 | Emotional facial recognition difficulties as primary deficit in children with attention deficit hyperactivity disorder: a systematic review | Not in English |
| Yang et al, 2017 | [A systematic review of seizure risk and efficacy of methylphenidate treatment on epilepsy combined with attention deficit hyperactivity disorder] | Not in English |
| Velo et al, 2013 | [Quality of life of patients with attention-deficit/hyperactivity disorder: systematic review of the past 5 years] | Not in English |
| Mate et al, 2015 | [Cognitive conceptualization of adult attention deficit hyperactivity disorder: a systematic review] | Not in English |
| Racicka et al, 2013 | Correlations between attention deficit hyperactivity disorder and obesity - a systematic review of the literature] | Not in English |
| Tan et al, 2011 | Relationship between serum ferritin levels and susceptibility to attention deficit hyperactivity disorder in children: a Meta analysis | Not in English |
| Schmitt et al, 2013 | Association of atopic eczema and attention-deficit/hyperactivity disorder - meta-analysis of epidemiologic studies | Not in English |
| Balint et al, 2015 | Neurobiological correlates of cognitive flexibility in ADHD - A systematic review of the literature | Not in English |
| Park et al, 2015 | [Effects of Cognitive Behavioral Therapy on Attention Deficit Hyperactivity Disorder among School-aged Children in Korea: A Meta-Analysis] | Not in English |
| Kimko et al, 2012 | Population pharmacodynamic modeling of various extended-release formulations of methylphenidate in children with attention deficit hyperactivity disorder via meta-analysis | Did not met minimum inclusion criteria |
| Kaiser et al, 2015 | What is the evidence of impaired motor skills and motor control among children with attention deficit hyperactivity disorder (ADHD)? Systematic review of the literature | Did not met minimum inclusion criteria |
| Ng et al, 2017 | A Systematic Review of the Use of Bupropion for Attention-Deficit/Hyperactivity Disorder in Children and Adolescents | Did not met minimum inclusion criteria |
| Sánchez-Mora et al, 2011 | Exploring DRD4 and its interaction with SLC6A3 as possible risk factors for adult ADHD: a meta-analysis in four European populations | Did not met minimum inclusion criteria |
| Alderson et al, 2007 | Attention-deficit/hyperactivity disorder and behavioral inhibition: a meta-analytic review of the stop-signal paradigm | Not a systematic review |
| Barkley et al, 2008 | Unrecognized attention-deficit/hyperactivity disorder in adults presenting with other psychiatric disorders | Not a systematic review |
| Biederman et al, 2007 | Effect of comorbid symptoms of oppositional defiant disorder on responses to atomoxetine in children with ADHD: a meta-analysis of controlled clinical trial data | Not a systematic review |
| Black et al, 2009 | Sitting-meditation interventions among youth: a review of treatment efficacy | Not a systematic review |
| Charach et al, 2008 | Improving psychostimulant adherence in children with ADHD | Not a systematic review |
| Davids et al, 2005 | Attention deficit hyperactivity disorder and borderline personality disorder | Not a systematic review |
| Donnelly et al, 2004 | Cost-effectiveness of dexamphetamine and methylphenidate for the treatment of childhood attention deficit hyperactivity disorder | Not a systematic review |
| Franke et al, 2010 | Multicenter analysis of the SLC6A3/DAT1 VNTR haplotype in persistent ADHD suggests differential involvement of the gene in childhood and persistent ADHD | Not a systematic review |
| Hill et al, 1996 | Age-dependent decline of attention deficit hyperactivity disorder | Not a systematic review |
| Knouse et al, 2008 | Recent developments in the psychosocial treatment of adult ADHD | Not a systematic review |
| Maher et al, 2002 | Dopamine system genes and attention deficit hyperactivity disorder: a meta-analysis | Not a systematic review |
| Monastra et al, 2005 | Electroencephalographic biofeedback in the treatment of attention-deficit/hyperactivity disorder | Not a systematic review |
| Nigg et al, 2002 | Big five dimensions and ADHD symptoms: links between personality traits and clinical symptoms | Not a systematic review |
| Polanczyk et al, 2008 | Epidemiologic considerations in attention deficit hyperactivity disorder: a review and update | Not a systematic review |
| Polzser et al, 2007 | Meta-analysis of aggression or hostility events in randomized, controlled clinical trials of atomoxetine for ADHD | Not a systematic review |
| Rohde et al, 2004 | [Recent advances on attention deficit/hyperactivity disorder] | Not a systematic review |
| Stawicki et al, 2006 | Family psychiatric history evidence on the nosological relations of DSM-IV ADHD combined and inattentive subtypes: new data and meta-analysis | Not a systematic review |
| Walitza et al, 2010 | Prospective follow-up studies found no chromosomal mutagenicity of methylphenidate therapy in ADHD affected children | Not a systematic review |
| Wilens et al, 2006 | Do children and adolescents with ADHD respond differently to atomoxetine? | Not a systematic review |
| Wilens et al, 2006 | Long-term atomoxetine treatment in adolescents with attention-deficit/hyperactivity disorder | Not a systematic review |
| Zappitelli et al, 2001 | Pre-, peri-, and postnatal trauma in subjects with attention-deficit hyperactivity disorder | Not a systematic review |
| Mazza et al, 2013 | Drugs for attention deficit-hyperactivity disorder do not increase the mid-term risk of sudden death in children: a meta-analysis of observational studies | Not a systematic review |
| Cortese et al, 2021 | A Practical, Evidence-informed Approach to Managing Stimulant-Refractory Attention Deficit Hyperactivity Disorder (ADHD) | Not a systematic review |
| Buoli et al, 2016 | Alternative pharmacological strategies for adult ADHD treatment: a systematic review | Did not met minimum inclusion criteria |
| Uchida et al, 2021 | Assessing the Magnitude of Risk for ADHD in Offspring of Parents with ADHD: A Systematic Literature Review and Meta-Analysis | Did not met minimum inclusion criteria |
| Middeldorp et al, 2016 | A Genome-Wide Association Meta-Analysis of Attention-Deficit/Hyperactivity Disorder Symptoms in Population-Based Pediatric Cohorts | Did not met minimum inclusion criteria |
| Nikkelen et al, 2014 | Media use and ADHD-related behaviors in children and adolescents: A meta-analysis | Did not met minimum inclusion criteria |
| Zaso et al, 2015 | Treatments for Adolescents With Comorbid ADHD and Substance Use Disorder: A Systematic Review | Did not met minimum inclusion criteria |
| Craig et al, 2020 | ADHD and Exposure to Maltreatment in Children and Youth: a Systematic Review of the Past 10 Years | Did not met minimum inclusion criteria |
| Rahi et al, 2021 | Animal models of attention-deficit hyperactivity disorder (ADHD) | Not a systematic review |
| Bondopadhyay et al, 2021 | The Role of the Circadian System in Attention Deficit Hyperactivity Disorder | Not a systematic review |
| Magnus et al, 2021 | Attention Deficit Hyperactivity Disorder | Not a systematic review |
| Ota et al, 2021 | Evaluating Guanfacine Hydrochloride in the Treatment of Attention Deficit Hyperactivity Disorder (ADHD) in Adult Patients: Design, Development and Place in Therapy | Not a systematic review |
| Sanchez-Mora et al, 2010 | Meta-analysis of brain-derived neurotrophic factor p.Val66Met in adult ADHD in four European populations | Not a systematic review |
| Takahashi et al, 2021 | Peripheral biomarkers of attention-deficit hyperactivity disorder: Current status and future perspective | Not a systematic review |
| Ditrich et al, 2021 | Borderline personality disorder (BPD) and attention deficit hyperactivity disorder (ADHD) revisited - a review-update on common grounds and subtle distinctions | Not a systematic review |
| Moore et al, 2018 | Context and Implications Document for: School-based interventions for attention-deficit/hyperactivity disorder: A systematic review with multiple synthesis methods | Not a systematic review |
| Bong et al, 2021 | The Role of Quantitative Electroencephalogram in the Diagnosis and Subgrouping of Attention-Deficit/Hyperactivity Disorder | Not a systematic review |
| Powell et al, 2021 | Investigating regions of shared genetic variation in attention deficit/hyperactivity disorder and major depressive disorder: a GWAS meta-analysis | Not a systematic review |
| Rubia et al, 2021 | Neurotherapeutics for Attention Deficit/Hyperactivity Disorder (ADHD): A Review | Not a systematic review |
| Retz et al, 2021 | Attention-Deficit/Hyperactivity Disorder (ADHD), antisociality and delinquent behavior over the lifespan | Not a systematic review |
| Ornoy et al, 2021 | The Effects of Drugs used for the Treatment of Attention Deficit Hyperactivity Disorder (ADHD) on Pregnancy Outcome and Breast-feeding: A Critical Review | Not a systematic review |
| Wright et al, 2021 | Psychological and neuropsychological underpinnings of attention-deficit/hyperactivity disorder assessment | Not a systematic review |
| Pereira-Sanchez et al, 2021 | Systematic Review: Medication Effects on Brain Intrinsic Functional Connectivity in Patients With Attention-Deficit/Hyperactivity Disorder | Not a systematic review |
| Muzwagi et al, 2021 | How Are Attention-deficit Hyperactivity and Internet Gaming Disorders Related in Children and Youth? | Not a systematic review |
| Fabiano et al, 2015 | A systematic review of meta-analyses of psychosocial treatment for attention-deficit/hyperactivity disorder | Not a systematic review |
| Periyasamy et al, 2021 | Machine Learning Techniques for the Diagnosis of Attention-Deficit/Hyperactivity Disorder from Magnetic Resonance Imaging: A Concise Review | Not a systematic review |
| Prakash et al, 2021 | Adult attention-deficit Hyperactivity disorder: From clinical reality toward conceptual clarity | Not a systematic review |
| Urbano et al, 2021 | The Link between Pediatric Obstructive Sleep Apnea (OSA) and Attention Deficit Hyperactivity Disorder (ADHD) | Not a systematic review |
| Rheims et al, 2021 | Attention deficit/hyperactivity disorder and epilepsy | Not a systematic review |
| Shaw et al, 2021 | Adolescent Attention-Deficit/Hyperactivity Disorder: Understanding Teenage Symptom Trajectories | Not a systematic review |
| Edinoff et al, 2021 | Viloxazine in the Treatment of Attention Deficit Hyperactivity Disorder | Not a systematic review |
| Mattingly et al, 2021 | Individualization of attention-deficit/hyperactivity disorder treatment: pharmacotherapy considerations by age and co-occurring conditions | Not a systematic review |
| Pringsheim et al, 2015 | The pharmacological management of oppositional behaviour, conduct problems, and aggression in children and adolescents with attention-deficit hyperactivity disorder, oppositional defiant disorder, and conduct disorder: a systematic review and meta-analysis. Part 1: psychostimulants, alpha-2 agonists, and atomoxetine | Not a systematic review |
| Bonvicini et al, 2016 | Attention-deficit hyperactivity disorder in adults: a systematic review and meta-analysis of genetic, pharmacogenetic and biochemical studies | Not a systematic review |
| Chiarenza et al, 2021 | Quantitative EEG in Childhood Attention Deficit Hyperactivity Disorder and Learning Disabilities | Not a systematic review |
| Lipka et al, 2021 | Resolving heterogeneity in transcranial electrical stimulation efficacy for attention deficit hyperactivity disorder | Not a systematic review |
| Yadav et al, 2021 | Genetic variations influence brain changes in patients with attention-deficit hyperactivity disorder | Not a systematic review |
| Rivero et al, 2016 | Corrigendum: ADHD Rehabilitation through Video Gaming: A Systematic Review Using PRISMA Guidelines of the Current Findings and the Associated Risk of Bias | Not a systematic review |
| APA, 2004 | Meta-Analysis of Intellectual and Neuropsychological Test Performance in Attention-Deficit/Hyperactivity Disorder | Did not met minimum inclusion criteria |
| Arns et al, 2009 | Efficacy of neurofeedback treatment in ADHD: the effects on inattention, impulsivity and hyperactivity: a meta-analysis | Did not met minimum inclusion criteria |
| Bálint et al, 2009 | Attention deficit hyperactivity disorder (ADHD): gender- and age-related differences in neurocognition | Did not met minimum inclusion criteria |
| Bergen et al, 2007 | Age-related changes in heritability of behavioral phenotypes over adolescence and young adulthood: a meta-analysis | Did not met minimum inclusion criteria |
| Banaschewski et al, 2006 | Long-acting medications for the hyperkinetic disorders. A systematic review and European treatment guideline | Did not met minimum inclusion criteria |
| Banaschewski et al, 2008 | [Long-acting medications for the treatment of hyperkinetic disorders - a systematic review and European treatment guideline. Part 1: overview and recommendations] | Did not met minimum inclusion criteria |
| Bobb et al, 2005 | Molecular genetic studies of ADHD: 1991 to 2004 | Did not met minimum inclusion criteria |
| Boonstra et al, 2005 | Executive functioning in adult ADHD: a meta-analytic review | Did not met minimum inclusion criteria |
| Boutros et al, 2005 | A four-step approach for developing diagnostic tests in psychiatry: EEG in ADHD as a test case | Did not met minimum inclusion criteria |
| Bridgett et al, 2006 | Intellectual functioning in adults with ADHD: A meta-analytic examination of full scale IQ differences between adults with and without ADHD | Did not met minimum inclusion criteria |
| Cheuk et al, 2006 | Meta-analysis of association between a catechol-O-methyltransferase gene polymorphism and attention deficit hyperactivity disorder | Did not met minimum inclusion criteria |
| Cortese et al, 2009 | Sleep in children with attention-deficit/hyperactivity disorder: meta-analysis of subjective and objective studies | Did not met minimum inclusion criteria |
| Cortese et al, 2006 | Sleep and alertness in children with attention-deficit/hyperactivity disorder: a systematic review of the literature | Did not met minimum inclusion criteria |
| Fazel et al, 2008 | Mental disorders among adolescents in juvenile detention and correctional facilities: a systematic review and metaregression analysis of 25 surveys | Did not met minimum inclusion criteria |
| Dickstein et al, 2006 | The neural correlates of attention deficit hyperactivity disorder: an ALE meta-analysis | Did not met minimum inclusion criteria |
| Ellison-Wright et al, 2008 | Structural brain change in Attention Deficit Hyperactivity Disorder identified by meta-analysis | Did not met minimum inclusion criteria |
| Fabiano et al, 2007 | Father participation in behavioral parent training for ADHD: review and recommendations for increasing inclusion and engagement | Did not met minimum inclusion criteria |
| Faraone et al, 2002 | Efficacy of Adderall for Attention-Deficit/Hyperactivity Disorder: a meta-analysis | Did not met minimum inclusion criteria |
| Faraone et al, 2002 | Comparative efficacy of Adderall and methylphenidate in attention-deficit/hyperactivity disorder: a meta-analysis | Did not met minimum inclusion criteria |
| Faraone et al, 2006 | Comparing the efficacy of medications for ADHD using meta-analysis | Did not met minimum inclusion criteria |
| Faraone et al, 2010 | Comparing the efficacy of stimulants for ADHD in children and adolescents using meta-analysis | Did not met minimum inclusion criteria |
| Faraone et al, 2001 | Meta-analysis of the association between the 7-repeat allele of the dopamine D(4) receptor gene and attention deficit hyperactivity disorder | Did not met minimum inclusion criteria |
| Faraone et al, 2010 | A comparison of the efficacy of medications for adult attention-deficit/hyperactivity disorder using meta-analysis of effect sizes | Did not met minimum inclusion criteria |
| Faraone et al, 2004 | Meta-analysis of the efficacy of methylphenidate for treating adult attention-deficit/hyperactivity disorder | Did not met minimum inclusion criteria |
| Faraone et al, 2003 | Does stimulant treatment lead to substance use disorders? | Did not met minimum inclusion criteria |
| Gaub et al, 1997 | Gender differences in ADHD: a meta-analysis and critical review | Did not met minimum inclusion criteria |
| Gershon et al, 2002 | A meta-analytic review of gender differences in ADHD | Did not met minimum inclusion criteria |
| Gizer et al, 2009 | Candidate gene studies of ADHD: a meta-analytic review | Did not met minimum inclusion criteria |
| Hervey et al, 2004 | Neuropsychology of adults with attention-deficit/hyperactivity disorder: a meta-analytic review | Did not met minimum inclusion criteria |
| Homack et al, 2004 | A meta-analysis of the sensitivity and specificity of the Stroop Color and Word Test with children | Did not met minimum inclusion criteria |
| Huang-Pollock et al, 2003 | Searching for the attention deficit in attention deficit hyperactivity disorder: the case of visuospatial orienting | Did not met minimum inclusion criteria |
| Huizenga et al, 2009 | Task complexity enhances response inhibition deficits in childhood and adolescent attention-deficit/hyperactivity disorder: a meta-regression analysis | Did not met minimum inclusion criteria |
| Hutchinson et al, 2008 | Corpus callosum morphology in children and adolescents with attention deficit hyperactivity disorder: a meta-analytic review | Did not met minimum inclusion criteria |
| Lansbergen et al, 2007 | Stroop interference and attention-deficit/hyperactivity disorder: A review and meta-analysis | Did not met minimum inclusion criteria |
| Levinson et al, 2005 | Meta-analysis in psychiatric genetics | Did not met minimum inclusion criteria |
| Li et al, 2006 | Meta-analysis shows significant association between dopamine system genes and attention deficit hyperactivity disorder (ADHD) | Did not met minimum inclusion criteria |
| Lijffijt et al, 2005 | A meta-analytic review of stopping performance in attention-deficit/hyperactivity disorder: deficient inhibitory motor control? | Did not met minimum inclusion criteria |
| Majewicz-Hefley et al, 2007 | A meta-analysis of combined treatments for children diagnosed with ADHD | Did not met minimum inclusion criteria |
| Martinussen et al, 2005 | A meta-analysis of working memory impairments in children with attention-deficit/hyperactivity disorder | Did not met minimum inclusion criteria |
| Mészáros et al, 2009 | Pharmacotherapy of adult attention deficit hyperactivity disorder (ADHD): a meta-analysis | Did not met minimum inclusion criteria |
| Miller et al, 2008 | Direct comparisons of treatment modalities for youth disorders: a meta-analysis | Did not met minimum inclusion criteria |
| Mullane et al, 2009 | Interference control in children with and without ADHD: a systematic review of Flanker and Simon task performance | Did not met minimum inclusion criteria |
| Oosterlaan et al, 1998 | Response inhibition in AD/HD, CD, comorbid AD/HD + CD, anxious, and control children: a meta-analysis of studies with the stop task | Did not met minimum inclusion criteria |
| Pelham et al, 2007 | The economic impact of attention-deficit/hyperactivity disorder in children and adolescents | Did not met minimum inclusion criteria |
| Polanczyk et al, 2005 | Attention-deficit/hyperactivity disorder: advancing on pharmacogenomics | Did not met minimum inclusion criteria |
| Purper-Ouakil et al, 2005 | Meta-analysis of family-based association studies between the dopamine transporter gene and attention deficit hyperactivity disorder | Did not met minimum inclusion criteria |
| Raz et al, 2009 | Essential fatty acids and attention-deficit-hyperactivity disorder: a systematic review | Did not met minimum inclusion criteria |
| Romine et al, 2004 | Wisconsin Card Sorting Test with children: a meta-analytic study of sensitivity and specificity | Did not met minimum inclusion criteria |
| Schoechlin et al, 2005 | Neuropsychological performance in adult attention-deficit hyperactivity disorder: meta-analysis of empirical data | Did not met minimum inclusion criteria |
| Schwartz et al, 2008 | ADHD and Stroop interference from age 9 to age 41 years: a meta-analysis of developmental effects | Did not met minimum inclusion criteria |
| Silva et al, 1996 | Carbamazepine use in children and adolescents with features of attention-deficit hyperactivity disorder: a meta-analysis | Did not met minimum inclusion criteria |
| Simon et al, 2009 | Prevalence and correlates of adult attention-deficit hyperactivity disorder: meta-analysis | Did not met minimum inclusion criteria |
| Van der Oord et al, 2008 | Efficacy of methylphenidate, psychosocial treatments and their combination in school-aged children with ADHD: a meta-analysis | Did not met minimum inclusion criteria |
| van Mourik et al, 2005 | The Stroop revisited: a meta-analysis of interference control in AD/HD | Did not met minimum inclusion criteria |
| Wilens et al, 2003 | Drug therapy for adults with attention-deficit hyperactivity disorder | Did not met minimum inclusion criteria |
| Wilens et al, 2008 | Misuse and diversion of stimulants prescribed for ADHD: a systematic review of the literature | Did not met minimum inclusion criteria |
| Wilens et al, 1995 | Pharmacotherapy of adult attention deficit/hyperactivity disorder: a review | Did not met minimum inclusion criteria |
| Wilens et al, 2003 | Does stimulant therapy of attention-deficit/hyperactivity disorder beget later substance abuse? A meta-analytic review of the literature | Did not met minimum inclusion criteria |
| Wilens et al, 2005 | The clinical dilemma of using medications in substance-abusing adolescents and adults with attention-deficit/hyperactivity disorder: what does the literature tell us? | Did not met minimum inclusion criteria |
| Wilens et al, 2002 | A review of the pharmacotherapy of adults with attention-deficit/hyperactivity disorder | Did not met minimum inclusion criteria |
| Willcutt et al, 2005 | Validity of the executive function theory of attention-deficit/hyperactivity disorder: a meta-analytic review | Did not met minimum inclusion criteria |
| Wingo et al, 2007 | A systematic review of rates and diagnostic validity of comorbid adult attention-deficit/hyperactivity disorder and bipolar disorder | Did not met minimum inclusion criteria |
| Yang, B. et al, 2007 | A meta-analysis of association studies between the 10-repeat allele of a VNTR polymorphism in the 3'-UTR of dopamine transporter gene and attention deficit hyperactivity disorder | Did not met minimum inclusion criteria |
| Zhou et al, 2008 | Meta-analysis of genome-wide linkage scans of attention deficit hyperactivity disorder | Did not met minimum inclusion criteria |
| Lee et al, 2018 | BDNF 196 G/A and COMT Val158Met Polymorphisms and Susceptibility to ADHD: A Meta-Analysis | Did not met minimum inclusion criteria |
| Agarwal et al, 2012 | The quality of life of adults with attention deficit hyperactivity disorder: a systematic review | Did not met minimum inclusion criteria |
| Bussalb et al, 2019 | Clinical and Experimental Factors Influencing the Efficacy of Neurofeedback in ADHD: A Meta-Analysis | Did not met minimum inclusion criteria |
| Correll et al, 2021 | Systematic review of transdermal treatment options in attention-deficit/hyperactivity disorder: implications for use in adult patients | Did not met minimum inclusion criteria |
| Ramos et al, 2019 | A meta-analysis on verbal working memory in children and adolescents with ADHD | Did not met minimum inclusion criteria |
| Nakao et al, 2011 | Gray matter volume abnormalities in ADHD: voxel-based meta-analysis exploring the effects of age and stimulant medication | Did not met minimum inclusion criteria |
| Caisley et al, 2012 | Adherence to medication in adults with attention deficit hyperactivity disorder and pro re nata dosing of psychostimulants: a systematic review | Did not met minimum inclusion criteria |
| vanHulzen et al, 2017 | Genetic Overlap Between Attention-Deficit/Hyperactivity Disorder and Bipolar Disorder: Evidence From Genome-wide Association Study Meta-analysis | Did not met minimum inclusion criteria |
| Khoury et al, 2019 | Comparing Executive Functioning in Children and Adolescents With Fetal Alcohol Spectrum Disorders and ADHD: A Meta-Analysis | Did not met minimum inclusion criteria |
| Karaca et al, 2017 | Comorbidity between Behavioral Addictions and Attention Deficit/Hyperactivity Disorder: a Systematic Review | Did not met minimum inclusion criteria |
| Joseph et al, 2015 | Oxidative Stress and ADHD: A Meta-Analysis | Did not met minimum inclusion criteria |
| Ivanov et al, 2014 | Optimizing fitness for duty and post-combat clinical services for military personnel and combat veterans with ADHD-a systematic review of the current literature | Did not met minimum inclusion criteria |
| Molitor et al, 2017 | Using task performance to inform treatment planning for youth with ADHD: A systematic review | Did not met minimum inclusion criteria |
| Ravishankar et al, 2016 | The efficacy of atomoxetine in treating adult attention deficit hyperactivity disorder (ADHD): A meta-analysis of controlled trials | Did not met minimum inclusion criteria |
| Pineda-Alhucema et al, 2018 | Executive Function and Theory of Mind in Children with ADHD: a Systematic Review | Did not met minimum inclusion criteria |
| Levie et al, 2019 | Maternal Thyroid Function in Early Pregnancy and Child Attention-Deficit Hyperactivity Disorder: An Individual-Participant Meta-Analysis | Did not met minimum inclusion criteria |
| Nickel et al, 2019 | Systematic Review: Overlap Between Eating, Autism Spectrum, and Attention-Deficit/Hyperactivity Disorder | Did not met minimum inclusion criteria |
| Nageye et al, 2019 | Beyond stimulants: a systematic review of randomised controlled trials assessing novel compounds for ADHD | Did not met minimum inclusion criteria |
| Ghanizadeh et al, 2013 | Systematic review of clinical trials of aripiprazole for treating attention deficit hyperactivity disorder | Did not met minimum inclusion criteria |
| Rubia et al, 2014 | Effects of stimulants on brain function in attention-deficit/hyperactivity disorder: a systematic review and meta-analysis | Did not met minimum inclusion criteria |
| Tanaka et al, 2013 | A meta-analysis of the consistency of atomoxetine treatment effects in pediatric patients with attention-deficit/hyperactivity disorder from 15 clinical trials across four geographic regions | Did not minimum inclusion criteria |
| Fridman et al, 2015 | Predicted effect size of lisdexamfetamine treatment of attention deficit/hyperactivity disorder (ADHD) in European adults: Estimates based on indirect analysis using a systematic review and meta-regression analysis | Did not met minimum inclusion criteria |
| Grassmann et al, 2013 | Effects of low doses of polyunsaturated Fatty acids on the attention deficit/hyperactivity disorder of children: a systematic review | Did not met minimum inclusion criteria |
| Geburek et al, 2013 | Electrophysiological indices of error monitoring in juvenile and adult attention deficit hyperactivity disorder (ADHD)--a meta-analytic assessment | Did not met minimum inclusion criteria |
| McCarthy et al, 2014 | Identifying a consistent pattern of neural function in attention deficit hyperactivity disorder: a meta-analysis | Did not met minimum inclusion criteria |
| Levin et al, 2016 | Attention-deficit/hyperactivity disorder and eating disorders across the lifespan: A systematic review of the literature | Did not met minimum inclusion criteria |
| Razoki et al, 2018 | Neurofeedback versus psychostimulants in the treatment of children and adolescents with attention-deficit/hyperactivity disorder: a systematic review | Did not met minimum inclusion criteria |
| Pauli-Pott et al, 2011 | Neuropsychological basic deficits in preschoolers at risk for ADHD: a meta-analysis | Did not met minimum inclusion criteria |
| Li et al, 2021 | Polygenic Scores for ADHD: A Meta-Analysis | Did not met minimum inclusion criteria |
| Alderson et al, 2013 | Attention-Deficit/Hyperactivity Disorder (ADHD) and Working Memory in Adults: A Meta-Analytic Review | Did not met minimum inclusion criteria |
| Contini et al, 2013 | Pharmacogenetics of response to methylphenidate in adult patients with Attention-Deficit/Hyperactivity Disorder (ADHD): a systematic review | Did not met minimum inclusion criteria |
| Shahidullah et al, 2018 | Integrated care models for ADHD in children and adolescents: A systematic review | Did not met minimum inclusion criteria |
| Czerniak et al, 2013 | Areas of the brain modulated by single-dose methylphenidate treatment in youth with ADHD during task-based fMRI: a systematic review | Did not met minimum inclusion criteria |
| Graziano et al, 2016 | Attention-deficit hyperactivity disorder and children's emotion dysregulation: A meta-analysis | Did not met minimum inclusion criteria |
| Hodgson et al, 2014 | Nonpharmacological treatments for ADHD: a meta-analytic review | Did not met minimum inclusion criteria |
| Roberts et al, 2021 | Attention-deficit/hyperactivity disorder and risk-taking: A three-level meta-analytic review of behavioral, self-report, and virtual reality metrics | Did not met minimum inclusion criteria |
| Ye et al, 2016 | Two SNAP-25 genetic variants in the binding site of multiple microRNAs and susceptibility of ADHD: A meta-analysis | Did not met minimum inclusion criteria |
| Alhraiwil et al, 2015 | Systematic review of the epidemiology of attention deficit hyperactivity disorder in Arab countries | Did not met minimum inclusion criteria |
| vanEwijk et al, 2012 | Diffusion tensor imaging in attention deficit/hyperactivity disorder: a systematic review and meta-analysis | Did not met minimum inclusion criteria |
| Nikolas et al, 2010 | Genetic and environmental influences on ADHD symptom dimensions of inattention and hyperactivity: a meta-analysis | Did not met minimum inclusion criteria |
| Mulqueen et al, 2015 | Meta-analysis: parental interventions for preschool ADHD | Did not met minimum inclusion criteria |
| Ging-Jehli et al, 2021 | Improving neurocognitive testing using computational psychiatry—A systematic review for ADHD | Did not met minimum inclusion criteria |
| Pereira-Sanchez et al, 2021 | Neuroimaging in attention-deficit/hyperactivity disorder | Did not met minimum inclusion criteria |
| Mick et al, 2013 | Meta-analysis of increased heart rate and blood pressure associated with CNS stimulant treatment of ADHD in adults | Did not met minimum inclusion criteria |
| Thrower et al, 2019 | Prevalence of Autism Spectrum Disorder and Attention-Deficit Hyperactivity Disorder Amongst Individuals with Gender Dysphoria: A Systematic Review | Did not met minimum inclusion criteria |
| Uguz et al, 2018 | Maternal Antidepressant Use During Pregnancy and the Risk of Attention-Deficit/Hyperactivity Disorder in Children: A Systematic Review of the Current Literature | Did not met minimum inclusion criteria |
| Barrett et al, 2013 | To sleep or not to sleep: a systematic review of the literature of pharmacological treatments of insomnia in children and adolescents with attention-deficit/hyperactivity disorder | Did not met minimum inclusion criteria |
| Auvin et al, 2018 | Systematic review of the screening, diagnosis, and management of ADHD in children with epilepsy. Consensus paper of the Task Force on Comorbidities of the ILAE Pediatric Commission | Did not met minimum inclusion criteria |
| Poysophon et al, 2018 | Neurocognitive Deficits Associated With ADHD in Athletes: A Systematic Review | Did not met minimum inclusion criteria |
| Lee et al, 2018 | Meta-Analysis of Case-Control and Family-Based Associations Between the 5-HTTLPR L/S Polymorphism and Susceptibility to ADHD | Did not met minimum inclusion criteria |
| Adisetiyo et al, 2017 | Neuroimaging the neural correlates of increased risk for substance use disorders in attention-deficit/hyperactivity disorder-A systematic review | Did not met minimum inclusion criteria |
| Frodl et al, 2012 | Meta-analysis of structural MRI studies in children and adults with attention deficit hyperactivity disorder indicates treatment effects | Did not met minimum inclusion criteria |
| Xenaki et al, 2015 | Clinical, neuropsychological and structural convergences and divergences between Attention Deficit/Hyperactivity Disorder and Borderline Personality Disorder: A systematic review | Did not met minimum inclusion criteria |
| Kingdon et al, 2016 | Research Review: Executive function deficits in fetal alcohol spectrum disorders and attention-deficit/hyperactivity disorder - a meta-analysis | Did not met minimum inclusion criteria |
| Kasper et al, 2012 | Moderators of working memory deficits in children with attention-deficit/hyperactivity disorder (ADHD): a meta-analytic review | Did not met minimum inclusion criteria |
| Linton et al, 2013 | Antipsychotic and Psychostimulant Drug Combination Therapy in Attention Deficit/Hyperactivity and Disruptive Behavior Disorders: A Systematic Review of Efficacy and Tolerability | Did not met minimum inclusion criteria |
| Peisch et al, 2021 | Sensory processing and P300 event-related potential correlates of stimulant response in children with attention-deficit/hyperactivity disorder: A critical review | Did not met minimum inclusion criteria |
| McGrath et al, 2019 | Are there shared neural correlates between dyslexia and ADHD? A meta-analysis of voxel-based morphometry studies | Did not met minimum inclusion criteria |
| Liu et al, 2017 | The Association of SNAP25 Gene Polymorphisms in Attention Deficit/Hyperactivity Disorder: a Systematic Review and Meta-Analysis | Did not met minimum inclusion criteria |
| Lee et al, 2011 | Prospective association of childhood attention-deficit/hyperactivity disorder (ADHD) and substance use and abuse/dependence: a meta-analytic review | Did not met minimum inclusion criteria |
| Cairncross et al, 2016 | The Effectiveness of Mindfulness-Based Therapies for ADHD: A Meta-Analytic Review | Did not met minimum inclusion criteria |
| Kofler et al, 2013 | Reaction time variability in ADHD: a meta-analytic review of 319 studies | Did not met minimum inclusion criteria |
| Mihandoost et al, 2015 | Treatment Programs for Students With Attention Deficit Hyperactivity Disorder: A Meta-Analysis Study | Did not met minimum inclusion criteria |
| Micoulaud-Franchi et al, 2014 | EEG neurofeedback treatments in children with ADHD: an updated meta-analysis of randomized controlled trials | Did not met minimum inclusion criteria |
| Gao et al, 2019 | Impairments of large-scale functional networks in attention-deficit/hyperactivity disorder: a meta-analysis of resting-state functional connectivity | Did not met minimum inclusion criteria |
| Gomez et al, 2014 | ADHD and personality: a meta-analytic review | Did not met minimum inclusion criteria |
| Wylock et al, 2021 | Child attachment and ADHD: a systematic review | Did not met minimum inclusion criteria |
| Srivastav et al, 2018 | Emerging role of miRNA in attention deficit hyperactivity disorder: a systematic review | Did not met minimum inclusion criteria |
| Mohr-Jensen et al, 2019 | What Do Primary and Secondary School Teachers Know About ADHD in Children? Findings From a Systematic Review and a Representative, Nationwide Sample of Danish Teachers | Did not met minimum inclusion criteria |
| Mowinckel et al, 2015 | A meta-analysis of decision-making and attention in adults with ADHD | Did not met minimum inclusion criteria |
| Patros et al, 2016 | Choice-impulsivity in children and adolescents with attention-deficit/hyperactivity disorder (ADHD): A meta-analytic review | Did not met minimum inclusion criteria |
| Park et al, 2017 | Parental ADHD symptoms and parenting behaviors: A meta-analytic review | Did not met minimum inclusion criteria |
| Balazs et al, 2017 | Attention-deficit/hyperactivity disorder and suicide: A systematic review | Did not met minimum inclusion criteria |
| Kambeitz et al, 2014 | Meta-analysis of the association between dopamine transporter genotype and response to methylphenidate treatment in ADHD | Did not met minimum inclusion criteria |
| Rapport et al, 2013 | Do programs designed to train working memory, other executive functions, and attention benefit children with ADHD? A meta-analytic review of cognitive, academic, and behavioral outcomes | Did not met minimum inclusion criteria |
| Jackson et al, 2016 | Attention-Deficit/Hyperactivity Disorder and Monetary Delay Discounting: A Meta-Analysis of Case-Control Studies | Did not met minimum inclusion criteria |
| Hou et al, 2018 | Association of Serotonin Receptors with Attention Deficit Hyperactivity Disorder: A Systematic Review and Meta-analysis | Did not met minimum inclusion criteria |
| Lee et al, 2012 | A meta-analysis of behavioral parent training for children with attention deficit hyperactivity disorder | Did not met minimum inclusion criteria |
| Erturk et al, 2016 | Association of ADHD and Celiac Disease: What Is the Evidence? A Systematic Review of the Literature | Did not met minimum inclusion criteria |
| Wu et al, 2012 | Role of dopamine receptors in ADHD: a systematic meta-analysis | Did not met minimum inclusion criteria |
| Harpin et al, 2016 | Long-Term Outcomes of ADHD: A Systematic Review of Self-Esteem and Social Function | Did not met minimum inclusion criteria |
| Faraone et al, 2012 | Examining the comorbidity between attention deficit hyperactivity disorder and bipolar I disorder: a meta-analysis of family genetic studies | Did not met minimum inclusion criteria |
| Sebastian et al, 2014 | Frontal dysfunctions of impulse control - a systematic review in borderline personality disorder and attention-deficit/hyperactivity disorder | Did not met minimum inclusion criteria |
| Vidal-Estrada et al, 2012 | Psychological treatment of attention deficit hyperactivity disorder in adults: a systematic review | Did not met minimum inclusion criteria |
| vanLieshout et al, 2013 | Does neurocognitive functioning predict future or persistence of ADHD? A systematic review | Did not met minimum inclusion criteria |
| vanAmsterdam et al, 2018 | Causal Factors of Increased Smoking in ADHD: A Systematic Review | Did not met minimum inclusion criteria |
| Thagaard et al, 2016 | Empirical tests of natural selection-based evolutionary accounts of ADHD: a systematic review | Did not met minimum inclusion criteria |
| Tan et al, 2016 | A Meta-Analytic Review of the Efficacy of Physical Exercise Interventions on Cognition in Individuals with Autism Spectrum Disorder and ADHD | Did not met minimum inclusion criteria |
| Norman et al, 2016 | Structural and Functional Brain Abnormalities in Attention-Deficit/Hyperactivity Disorder and Obsessive-Compulsive Disorder: A Comparative Meta-analysis | Did not met minimum inclusion criteria |
| Hazell et al, 2011 | Core ADHD symptom improvement with atomoxetine versus methylphenidate: a direct comparison meta-analysis | Did not met minimum inclusion criteria |
| Hawkey et al, 2014 | Omega-3 fatty acid and ADHD: blood level analysis and meta-analytic extension of supplementation trials | Did not met minimum inclusion criteria |
| Dekkers et al, 2018 | Decision-Making Deficits in ADHD Are Not Related to Risk Seeking But to Suboptimal Decision-Making: Meta-Analytical and Novel Experimental Evidence | Did not met minimum inclusion criteria |
| Coogan et al, 2017 | A systematic review of circadian function, chronotype and chronotherapy in attention deficit hyperactivity disorder | Did not met minimum inclusion criteria |
| Vaa et al, 2014 | ADHD and relative risk of accidents in road traffic: a meta-analysis | Did not met minimum inclusion criteria |
| Treuer et al, 2016 | Factors affecting treatment adherence to atomoxetine in ADHD: a systematic review | Did not met minimum inclusion criteria |
| Willcutt et al, 2012 | The prevalence of DSM-IV attention-deficit/hyperactivity disorder: a meta-analytic review | Did not met minimum inclusion criteria |
| Whitely et al, 2019 | Attention deficit hyperactivity disorder late birthdate effect common in both high and low prescribing international jurisdictions: a systematic review | Did not met minimum inclusion criteria |
| Wallace et al, 2019 | A meta-analysis of malingering detection measures for attention-deficit/hyperactivity disorder | Did not met minimum inclusion criteria |
| Harikumar et al, 2021 | A Review of the Default Mode Network in Autism Spectrum Disorders and Attention Deficit Hyperactivity Disorder | Did not met minimum inclusion criteria |
| Ros et al, 2018 | Social Functioning in Children With or At Risk for Attention Deficit/Hyperactivity Disorder: A Meta-Analytic Review | Did not met minimum inclusion criteria |
| Lei et al, 2015 | Functional MRI reveals different response inhibition between adults and children with ADHD | Did not met minimum inclusion criteria |
| Kok et al, 2016 | Problematic Peer Functioning in Girls with ADHD: A Systematic Literature Review | Did not met minimum inclusion criteria |
| Rash et al, 2012 | Attention-deficit hyperactivity disorder and cardiac vagal control: a systematic review | Did not met minimum inclusion criteria |
| Polanczyk et al, 2014 | ADHD prevalence estimates across three decades: an updated systematic review and meta-regression analysis | Did not met minimum inclusion criteria |
| Gayleard et al, 2017 | Atomoxetine treatment for children and adolescents with Attention-Deficit/Hyperactivity Disorder (ADHD): a comprehensive meta-analysis of outcomes on parent-rated core symptomatology | Did not met minimum inclusion criteria |
| Grassmann et al, 2017 | Possible Cognitive Benefits of Acute Physical Exercise in Children With ADHD: A Systematic Review | Did not met minimum inclusion criteria |
| Bushe et al, 2014 | Systematic review of atomoxetine data in childhood and adolescent attention-deficit hyperactivity disorder 2009-2011: focus on clinical efficacy and safety | Did not met minimum inclusion criteria |
| Lau -Zhu et al, 2019 | Overlaps and distinctions between attention deficit/hyperactivity disorder and autism spectrum disorder in young adulthood: Systematic review and guiding framework for EEG-imaging research | Did not met minimum inclusion criteria |
| Nigg et al, 2012 | Meta-analysis of attention-deficit/hyperactivity disorder or attention-deficit/hyperactivity disorder symptoms, restriction diet, and synthetic food color additives | Did not met minimum inclusion criteria |
| Maloy et al, 2014 | A meta-analysis of the effectiveness of music interventions for children and adolescents with attention-deficit/hyperactivity disorder | Did not met minimum inclusion criteria |
| Wills et al, 2017 | Attention-Deficit/Hyperactivity Disorder in Looked-After Children: a Systematic Review of the Literature | Did not met minimum inclusion criteria |
| Sun et al, 2014 | Role of COMT in ADHD: a systematic meta-analysis | Did not met minimum inclusion criteria |
| Shiffrin et al, 2013 | No association between MspI allele of the ADRA2A polymorphism and ADHD: meta-analysis of family-based studies | Did not met minimum inclusion criteria |
| Nikolaidis et al, 2010 | ADHD and the DRD4 exon III 7-repeat polymorphism: an international meta-analysis | Did not met minimum inclusion criteria |
| Saletin et al, 2019 | A coordinate-based meta-analysis comparing brain activation between attention deficit hyperactivity disorder and total sleep deprivation | Did not met minimum inclusion criteria |
| Neudecker et al, 2019 | Exercise Interventions in Children and Adolescents With ADHD: A Systematic Review | Did not met minimum inclusion criteria |
| Hodgkins et al, 2011 | A systematic review of global publication trends regarding long-term outcomes of ADHD | Did not met minimum inclusion criteria |
| Leroy et al, 2021 | Emotional scene processing in children and adolescents with attention deficit/hyperactivity disorder: a systematic review | Did not met minimum inclusion criteria |
| Schulze et al, 2021 | Disentangling ADHD's Presentation-Related Decision-Making-A Meta-Analytic Approach on Predominant Presentations | Did not met minimum inclusion criteria |
| Parvataneni et al, 2020 | Perspective on Melatonin Use for Sleep Problems in Autism and Attention-Deficit Hyperactivity Disorder: A Systematic Review of Randomized Clinical Trials | Did not met minimum inclusion criteria |
| Praveena et al, 2020 | Phthalates exposure and attention-deficit/hyperactivity disorder in children: a systematic review of epidemiological literature | Did not met minimum inclusion criteria |
| Ramos et al, 2020 | A meta-analysis on verbal working memory in children and adolescents with ADHD | Did not met minimum inclusion criteria |
| Mucci et al, 2019 | ADHD with Comorbid Bipolar Disorders: A Systematic Review of Neurobiological, Clinical and Pharmacological Aspects Across the Lifespan | Full text not available |
| Danckaerts et al, 2010 | The quality of life of children with attention deficit/hyperactivity disorder: a systematic review | Not performed quality assessment of included primary studies |
| Kofler et al, 2008 | Quantifying ADHD classroom inattentiveness, its moderators, and variability: a meta-analytic review | Not performed quality assessment of included primary studies |
| Valera et al, 2007 | Meta-analysis of structural imaging findings in attention-deficit/hyperactivity disorder | Not performed quality assessment of included primary studies |
| Momany et al, 2018 | A Meta-Analysis of the Association Between Birth Weight and Attention Deficit Hyperactivity Disorder | Not performed quality assessment of included primary studies |
| Gobbo et al, 2014 | Influence of stimulant and non-stimulant drug treatment on driving performance in patients with attention deficit hyperactivity disorder: a systematic review | Not performed quality assessment of included primary studies |
| Pievsky et al, 2018 | The Neurocognitive Profile of Attention-Deficit/Hyperactivity Disorder: A Review of Meta-Analyses | Not performed quality assessment of included primary studies |
| Reichow et al, 2013 | Systematic review and meta-analysis of pharmacological treatment of the symptoms of attention-deficit/hyperactivity disorder in children with pervasive developmental disorders | Not performed quality assessment of included primary studies |
| Aoki et al, 2013 | Age-related change of neurochemical abnormality in attention-deficit hyperactivity disorder: a meta-analysis | Not performed quality assessment of included primary studies |
| Bernhard et al, 2021 | Cortisol response to acute psychosocial stress in ADHD compared to conduct disorder and major depressive disorder: A systematic review | Not performed quality assessment of included primary studies |
| Xie et al, 2021 | Effectiveness of Physical Activity Intervention on ADHD Symptoms: A Systematic Review and Meta-Analysis | Not performed quality assessment of included primary studies |
| Joshi 2021 | Pharmacotherapy of attention deficit/hyperactivity disorder in individuals with autism spectrum disorder: A systematic review of the literature | Not performed quality assessment of included studies |
| Lee et al, 2017 | The Effectiveness of Mindfulness-Based Intervention in Attention on Individuals with ADHD: A Systematic Review | Not performed quality assessment of included studies |
| Patros et al, 2019 | Planning deficits in children with attention-deficit/hyperactivity disorder (ADHD): A meta-analytic review of tower task performance | Not performed quality assessment of included studies |
| Sibley et al, 2014 | Pharmacological and psychosocial treatments for adolescents with ADHD: an updated systematic review of the literature | Not performed quality assessment of included studies |
| Schatz et al, 2015 | Systematic Review of Patients' and Parents' Preferences for ADHD Treatment Options and Processes of Care | Not performed quality assessment of included studies |
| Song et al, 2016 | Enhanced Physical Activity Improves Selected Outcomes in Children With ADHD: Systematic Review | Not performed quality assessment of included studies |
| Young et al, 2015 | Co-morbid psychiatric disorders among incarcerated ADHD populations: a meta-analysis | Not performed quality assessment of included studies |
| Brunkhorst-Kanaan et al, 2021 | ADHD and accidents over the life span - A systematic review | Not performed quality assessment of included studies |
| vanEmmerik-vanOortmerssen et al, 2012 | Prevalence of attention-deficit hyperactivity disorder in substance use disorder patients: a meta-analysis and meta-regression analysis | Not performed quality assessment of included studies |
| Hennissen et al, 2017 | Cardiovascular Effects of Stimulant and Non-Stimulant Medication for Children and Adolescents with ADHD: A Systematic Review and Meta-Analysis of Trials of Methylphenidate, Amphetamines and Atomoxetine | Not performed quality assessment of included studies |
| Dutta et al, 2022 | Phytotherapy for Attention Deficit Hyperactivity Disorder (ADHD): A Systematic Review and Meta-analysis | Not performed quality assessment of included studies |
| Vázquez et al, 2022 | Effects of Caffeine Consumption on Attention Deficit Hyperactivity Disorder (ADHD) Treatment: A Systematic Review of Animal Studies | Not performed quality assessment of included studies |
| Song et al, 2021 | Does Exposure to General Anesthesia Increase Risk of ADHD for Children Before Age of Three? | Not performed quality assessment of included studies |
| Young et al, 2015 | A meta-analysis of the prevalence of attention deficit hyperactivity disorder in incarcerated populations | Not performed quality assessment of included studies |
| Vysniauske et al, 2016 | The Effects of Physical Exercise on Functional Outcomes in the Treatment of ADHD: A Meta-Analysis | Not performed quality assessment of included studies |
| Kim et al, 2019 | Prevalence of attention deficit hyperactivity disorder symptoms in narcolepsy: a systematic review | Not performed quality assessment of included studies |
| Riesco-Matias et al, 2019 | What Do Meta-Analyses Have to Say About the Efficacy of Neurofeedback Applied to Children With ADHD? Review of Previous Meta-Analyses and a New Meta-Analysis | Not performed quality assessment of included studies |
| Cawkwell et al, 2021 | Neurodevelopmental Effects of Cannabis Use in Adolescents and Emerging Adults with ADHD: A Systematic Review | Not performed quality assessment of included studies |
| Lovett et al, 2021 | Systematic Review: Educational Accommodations for Children and Adolescents With Attention-Deficit/Hyperactivity Disorder | Not performed quality assessment of included studies |
| Agostoni et al, 2017 | The Role of Omega-3 Fatty Acids in Developmental Psychopathology: A Systematic Review on Early Psychosis, Autism, and ADHD | Not performed quality assessment of included studies |
| Tung et al, 2016 | Patterns of Comorbidity Among Girls With ADHD: A Meta-analysis | Not performed quality assessment of included studies |
| Tatlow-Golden et al, 2016 | What do general practitioners know about ADHD? Attitudes and knowledge among first-contact gatekeepers: systematic narrative review | Not performed quality assessment of included studies |
| Zhang et al, 2018 | Peripheral brain-derived neurotrophic factor in attention-deficit/hyperactivity disorder: A comprehensive systematic review and meta-analysis | Not performed quality assessment of included studies. |
| Coghill et al, 2014 | Effects of methylphenidate on cognitive functions in children and adolescents with attention-deficit/hyperactivity disorder: evidence from a systematic review and a meta-analysis | Not performed quality assessment of included studies. |
| Bushe et al, 2011 | Atomoxetine in children and adolescents with attention-deficit/hyperactivity disorder. Systematic review of review papers 2009-2011. An update for clinicians | Not performed quality assessment of included studies. |
| Allely et al, 2014 | The association of ADHD symptoms to self-harm behaviours: a systematic PRISMA review | Not performed quality assessment of included studies. |
| Metin et al, 2012 | A meta-analytic study of event rate effects on Go/No-Go performance in attention-deficit/hyperactivity disorder | Not performed quality assessment of included studies. |
| Meinzer et al, 2014 | The co-occurrence of attention-deficit/hyperactivity disorder and unipolar depression in children and adolescents: a meta-analytic review | Not performed quality assessment of included studies. |
| Schwartz et al, 2014 | Efficacy and safety of atomoxetine in children and adolescents with attention-deficit/hyperactivity disorder: results from a comprehensive meta-analysis and metaregression | Not performed quality assessment of included studies. |
| Coughlin et al, 2015 | Meta-Analysis: Reduced Risk of Anxiety with Psychostimulant Treatment in Children with Attention-Deficit/Hyperactivity Disorder | Not performed quality assessment of included studies |
| Coates et al, 2015 | Parenting Interventions for ADHD: A Systematic Literature Review and Meta-Analysis | Not performed quality assessment of included studies |
| Serra-Pinheiro et al, 2013 | Is ADHD a risk factor independent of conduct disorder for illicit substance use? A meta-analysis and metaregression investigation | Not performed quality assessment of included studies |
| Drover et al, 2019 | Maternal Thyroid Function During Pregnancy or Neonatal Thyroid Function and Attention Deficit Hyperactivity Disorder: A Systematic Review | Not performed quality assessment of included studies |
| Lunsford-Avery et al, 2016 | Sleep disturbances in adolescents with ADHD: A systematic review and framework for future research | Not performed quality assessment of included studies |
| Marx et al, 2018 | ADHD and the Choice of Small Immediate Over Larger Delayed Rewards: A Comparative Meta-Analysis of Performance on Simple Choice-Delay and Temporal Discounting Paradigms | Not performed quality assessment of included studies |
| Gao et al, 2021 | Structural and Functional Brain Abnormalities in Internet Gaming Disorder and Attention-Deficit/Hyperactivity Disorder: A Comparative Meta-Analysis | Not performed quality assessment of included studies |
| Sandstrom et al, 2021 | Prevalence of attention-deficit/hyperactivity disorder in people with mood disorders: A systematic review and meta-analysis | Not performed quality assessment of included studies |
| Craig et al, 2019 | A systematic review of comorbidity between cerebral palsy, autism spectrum disorders and Attention Deficit Hyperactivity Disorder | Not performed quality assessment of included studies |
| Weyers et al, 2019 | Impaired Interparental Relationships in Families of Children With Attention-Deficit/Hyperactivity Disorder (ADHD) A Meta-Analysis | Not performed quality assessment of included studies |
| VanDoren et al, 2019 | Sustained effects of neurofeedback in ADHD: a systematic review and meta-analysis | Not performed quality assessment of included studies |
| Cortese et al, 2012 | Toward systems neuroscience of ADHD: a meta-analysis of 55 fMRI studies | Not performed quality assessment of included studies |
| Coghill et al, 2017 | Systematic review of quality of life and functional outcomes in randomized placebo-controlled studies of medications for attention-deficit/hyperactivity disorder | Not performed quality assessment of included studies |
| Gajria et al, 2014 | Adherence, persistence, and medication discontinuation in patients with attention-deficit/hyperactivity disorder - a systematic literature review | Not performed quality assessment of included studies |
| Faraone et al, 2019 | Sleep-Associated Adverse Events During Methylphenidate Treatment of Attention-Deficit/Hyperactivity Disorder: A Meta-Analysis | Not performed quality assessment of included studies |
| Pozzi et al, 2018 | Adverse drug events related to mood and emotion in paediatric patients treated for ADHD: A meta-analysis | Not performed quality assessment of included studies |
| Parsons et al, 2019 | A Comparison of Virtual Reality Classroom Continuous Performance Tests to Traditional Continuous Performance Tests in Delineating ADHD: a Meta-Analysis | Not performed quality assessment of included studies |
| Schmitt et al, 2010 | Is atopic disease a risk factor for attention-deficit/hyperactivity disorder? A systematic review | Not performed quality assessment of included studies |
| Sibley et al, 2016 | Method of adult diagnosis influences estimated persistence of childhood ADHD: a systematic review of longitudinal studies | Not performed quality assessment of included studies |
| Samea et al, 2019 | Brain alterations in children/adolescents with ADHD revisited: A neuroimaging meta-analysis of 96 structural and functional studies | Not performed quality assessment of included studies |
| Anand et al, 2017 | Attention-Deficit/Hyperactivity Disorder And Inflammation: What Does Current Knowledge Tell Us? A Systematic Review | Not performed quality assessment of included studies |
| Cheung et al, 2018 | Maternal Depression in Families of Children with ADHD: A Meta-Analysis | Not performed quality assessment of included studies |
| Pinzone et al, 2019 | Temperament correlates in adult ADHD: A systematic review( bigstar bigstar) | Not performed quality assessment of included studies |
| Kirova et al, 2019 | Are subsyndromal manifestations of attention deficit hyperactivity disorder morbid in children? A systematic qualitative review of the literature with meta-analysis | Not performed quality assessment of included studies |
| Hirota et al, 2014 | Alpha-2 agonists for attention-deficit/hyperactivity disorder in youth: a systematic review and meta-analysis of monotherapy and add-on trials to stimulant therapy | Not performed quality assessment of included studies |
| Hall et al, 2016 | The clinical utility of the continuous performance test and objective measures of activity for diagnosing and monitoring ADHD in children: a systematic review | Not performed quality assessment of included studies |
| Maron et al, 2021 | Oculomotor deficits in attention deficit hyperactivity disorder (ADHD): A systematic review and comprehensive meta-analysis | Not performed quality assessment of included studies |
| Lohr et al, 2021 | Intentional Discontinuation of Psychostimulants Used to Treat ADHD in Youth: A Review and Analysis | Not performed quality assessment of included studies |
| Vancampfort et al, 2016 | Dropout from physical activity interventions in children and adolescents with attention deficit hyperactivity disorder: A systematic review and meta-analysis | Not performed quality assessment of included studies |
| Coghill et al, 2013 | Long-acting methylphenidate formulations in the treatment of attention-deficit/hyperactivity disorder: a systematic review of head-to-head studies | Not performed quality assessment of included studies |
| Caye et al, 2016 | Predictors of persistence of ADHD into adulthood: a systematic review of the literature and meta-analysis | Not performed quality assessment of included studies |
| Azeredo et al, 2018 | ADHD, CD, and ODD: Systematic review of genetic and environmental risk factors | Not performed quality assessment of included studies |
| Gharehgazlou et al, 2021 | Cortical Gyrification Morphology in Individuals with ASD and ADHD across the Lifespan: A Systematic Review and Meta-Analysis | Not performed quality assessment of included studies |
| Baker et al, 2021 | Polypharmacy in the Management of Attention-Deficit/Hyperactivity Disorder in Children and Adolescents: A Review and Update | Not performed quality assessment of included studies |
| Biancardi et al, 2021 | Sleep EEG microstructure in children and adolescents with attention deficit hyperactivity disorder: a systematic review and meta-analysis | Not performed quality assessment of included studies |
| Ng et al, 2017 | Managing childhood and adolescent attention-deficit/hyperactivity disorder (ADHD) with exercise: A systematic review | Not performed quality assessment of included studies |
| Theule et al, 2019 | Exploring the Relationships Between Problem Gambling and ADHD: A Meta-Analysis | Not performed quality assessment of included studies |
| Theule et al, 2018 | Children's ADHD Interventions and Parenting Stress: A Meta-Analysis | Not performed quality assessment of included studies |
| GarciaMurillo et al, 2015 | Locomotor activity measures in the diagnosis of attention deficit hyperactivity disorder: Meta-analyses and new findings | Not performed quality assessment of included studies |
| Catala-Lopez et al, 2012 | Prevalence of attention deficit hyperactivity disorder among children and adolescents in Spain: a systematic review and meta-analysis of epidemiological studies | Not performed quality assessment of included studies |
| Wright et al, 2015 | Practitioner Review: Pathways to care for ADHD - a systematic review of barriers and facilitators | Not performed quality assessment of included studies |
| Treuer et al, 2013 | A Systematic Review of Combination Therapy with Stimulants and Atomoxetine for Attention-Deficit/Hyperactivity Disorder, Including Patient Characteristics, Treatment Strategies, Effectiveness, and Tolerability | Not performed quality assessment of included studies |
| Scassellati et al, 2012 | Biomarkers and attention-deficit/hyperactivity disorder: a systematic review and meta-analyses | Not performed quality assessment of included studies |
| Danckaerts et al, 2010 | The quality of life of children with attention deficit/hyperactivity disorder: a systematic review | Not performed quality assessment of included studies |
| Bushe et al, 2016 | A network meta-analysis of atomoxetine and osmotic release oral system methylphenidate in the treatment of attention-deficit/hyperactivity disorder in adult patients | Not performed quality assessment of included studies |
| Brancati et al, 2021 | Development of bipolar disorder in patients with attention-deficit/hyperactivity disorder: A systematic review and meta-analysis of prospective studies | Not performed quality assessment of included studies |
| Goodlad et al, 2013 | Lead and Attention-Deficit/Hyperactivity Disorder (ADHD) symptoms: a meta-analysis | Not performed quality assessment of included studies |
| Miguelez-Fernandez et al, 2018 | Evaluating attention-deficit/hyperactivity disorder using ecological momentary assessment: a systematic review | Not performed quality assessment of included studies |
| Cortese et al, 2021 | Systematic Review and Meta-analysis: Resting-State Functional Magnetic Resonance Imaging Studies of Attention-Deficit/Hyperactivity Disorder | Not performed quality assessment of included studies |
| HeilskovRytter et al, 2015 | Diet in the treatment of ADHD in children - a systematic review of the literature | Not performed quality assessment of included studies |
| Koenig et al, 2017 | Resting state vagal tone in attention deficit (hyperactivity) disorder: A meta-analysis | Not performed quality assessment of included studies |
| Andersson et al, 2020 | Research Review: The strength of the genetic overlap between ADHD and other psychiatric symptoms - a systematic review and meta-analysis | Not performed quality assessment of included studies |
| Boland et al, 2020 | A literature review and meta-analysis on the effects of ADHD medications on functional outcomes | Not performed quality assessment of included studies |
| Carucci et al, 2021 | Long term methylphenidate exposure and growth in children and adolescents with ADHD. A systematic review and meta-analysis | Not performed quality assessment of included studies |
| Lan et al, 2009 | Attention deficit hyperactivity disorder in children: comparative efficacy of traditional Chinese medicine and methylphenidate | Not performed quality assessment of included studies |
| Losier et al, 1996 | Error patterns on the continuous performance test in non-medicated and medicated samples of children with and without ADHD: a meta-analytic review | Not performed quality assessment of included studies |
| Paloyelis etal, 2007 | Functional MRI in ADHD: a systematic literature review | Not performed quality assessment of included studies |
| Fazel et al, 2008 | Mental disorders among adolescents in juvenile detention and correctional facilities: a systematic review and metaregression analysis of 25 surveys | No involvement of two independent reviewers |
| Machado-Nascimento et al, 2016 et al, | Speech-language pathology findings in Attention Deficit Hyperactivity Disorder: a systematic literature review | No involvement of two independent reviewers |
| Morales et al, 2018 | Antidepressant use during pregnancy and risk of autism spectrum disorder and attention deficit hyperactivity disorder: systematic review of observational studies and methodological considerations | No involvement of two independent reviewers |
| Salem et al, 2018 | ADHD is associated with migraine: a systematic review and meta-analysis | No involvement of two independent reviewers |
| Wong et al, 2012 | Traditional Oriental Herbal Medicine for Children and Adolescents with ADHD: A Systematic Review | No involvement of two independent reviewers |
| Chang et al, 2018 | Omega-3 Polyunsaturated Fatty Acids in Youths with Attention Deficit Hyperactivity Disorder: a Systematic Review and Meta-Analysis of Clinical Trials and Biological Studies | No involvement of two independent reviewers |
| Donzelli et al, 2019 | The Association between Lead and Attention-Deficit/Hyperactivity Disorder: A Systematic Review | No involvement of two independent reviewers |
| Jeyanthi et al, 2019 | Effect of physical exercises on attention, motor skill and physical fitness in children with attention deficit hyperactivity disorder: a systematic review | No involvement of two independent reviewers |
| Grunblatt et al, 2019 | Association study and a systematic meta-analysis of the VNTR polymorphism in the 3'-UTR of dopamine transporter gene and attention-deficit hyperactivity disorder | No involvement of two independent reviewers |
| Romani et al, 2018 | Face memory and face recognition in children and adolescents with attention deficit hyperactivity disorder: A systematic review | No involvement of two independent reviewers |
| Ching et al, 2019 | Evaluation of Methylphenidate Safety and Maximum-Dose Titration Rationale in Attention-Deficit/Hyperactivity Disorder: A Meta-analysis | No involvement of two independent reviewers |
| Hellstrom et al, 2019 | A Systematic Review of Polyvictimization among Children with Attention Deficit Hyperactivity or Autism Spectrum Disorder | No involvement of two independent reviewers |
| Deault et al, 2010 | A systematic review of parenting in relation to the development of comorbidities and functional impairments in children with attention-deficit/hyperactivity disorder (ADHD) | No involvement of two independent reviewers |
| Zang et al, 2019 | Impact of physical exercise on children with attention deficit hyperactivity disorders Evidence through a meta-analysis | No involvement of two independent reviewers |
| Cook et al, 2014 | The self-esteem of adults diagnosed with attention-deficit/hyperactivity disorder (ADHD): a systematic review of the literature | No involvement of two independent reviewers |
| Willis D et al, 2019 | Stand-Alone Social Skills Training for Youth with ADHD: A Systematic Review | No involvement of two independent reviewers |
| Porter et al, 2019 | Low-moderate prenatal alcohol exposure and offspring attention-deficit hyperactivity disorder (ADHD): systematic review and meta-analysis | No involvement of two independent reviewers |
| Hariri et al, 2015 | Magnesium, Iron, and Zinc Supplementation for the Treatment of Attention Deficit Hyperactivity Disorder: A Systematic Review on the Recent Literature | No involvement of two independent reviewers |
| Adeyemo et al, 2014 | Mild traumatic brain injury and ADHD: a systematic review of the literature and meta-analysis | No involvement of two independent reviewers |
| Young et al, 2016 | The Efficacy of Cognitive Behavioral Therapy for Adults With ADHD: A Systematic Review and Meta-Analysis of Randomized Controlled Trials | No involvement of two independent reviewers |
| StrahlerRivero et al, 2015 | ADHD Rehabilitation through Video Gaming: A Systematic Review Using PRISMA Guidelines of the Current Findings and the Associated Risk of Bias | No involvement of two independent reviewers |
| Fusar-Poli et al, 2012 | Striatal dopamine transporter alterations in ADHD: pathophysiology or adaptation to psychostimulants? A meta-analysis | No involvement of two independent reviewers |
| Sarris et al, 2011 | Complementary medicines (herbal and nutritional products) in the treatment of Attention Deficit Hyperactivity Disorder (ADHD): a systematic review of the evidence | No involvement of two independent reviewers |
| Gomez-Cano et al, 2021 | The role of psychosocial adversity in the aetiology and course of attention deficit hyperactivity disorder | No full text available |
| Ghanizadeh et al, 2013 | A systematic review of the efficacy and safety of desipramine for treating ADHD | No full text available |
| Tong et al, 2013 | Prevalence of ADHD in children of China: a systematic review and meta analysis | No full text available |
| MacLean et al, 2021 | Attention-Deficit/Hyperactivity Disorder and Sports: A Lifespan Perspective | No full text available |
| Momin et al, 2011 | Attention-deficit hyperactivity disorder symptoms and lead exposure in children: A systematic review of literature | No full text available |
| Spencer et al, 2016 | Examining the association between posttraumatic stress disorder and attention-deficit/hyperactivity disorder: a systematic review and meta-analysis | No full text available |
| Chau et al, 2017 | Oral Health of Children With Attention Deficit Hyperactivity Disorder: Systematic Review and Meta-Analysis | No full text available |
| Fox et al, 2020 | Effectiveness of Social Skills Interventions Incorporating Peer Interactions for Children With Attention Deficit Hyperactivity Disorder: A Systematic Review | No full text available |
| Valmiki et al, 2021 | Reinforcement and Compensatory Mechanisms in Attention-Deficit Hyperactivity Disorder: A Systematic Review of Case-Control Studies | Only one database searched |
| Chang et al, 2021 | Cortisol and inflammatory biomarker levels in youths with attention deficit hyperactivity disorder (ADHD): evidence from a systematic review with meta-analysis | Only one database searched |
| Rocco et al, 2021 | Time of onset and/or diagnosis of ADHD in European children: a systematic review | Only one database searched |
| Benner-Davis et al, 2007 | Attention deficit and hyperactivity disorder: controversies of diagnosis and safety of pharmacological and nonpharmacological treatment | Only one database searched |
| Biederman et al, 2004 | How informative are parent reports of attention-deficit/hyperactivity disorder symptoms for assessing outcome in clinical trials of long-acting treatments? A pooled analysis of parents' and teachers' reports | Only one database searched |
| Bloch et al, 2009 | Meta-analysis: treatment of attention-deficit/hyperactivity disorder in children with comorbid tic disorders | Only one database searched |
| Consoli et al, 2007 | Comorbidity with ADHD decreases response to pharmacotherapy in children and adolescents with acute mania: evidence from a meta-analysis | Only one database searched |
| Cortese et al, 2008 | Attention-deficit/hyperactivity disorder (ADHD) and obesity: a systematic review of the literature | Only one database searched |
| Forero et al, 2009 | Candidate genes involved in neural plasticity and the risk for attention-deficit hyperactivity disorder: a meta-analysis of 8 common variants | Only one database searched |
| Huey et al, 2008 | Evidence-based psychosocial treatments for ethnic minority youth |  |
| Jin et al, 2004 | Methylphenidate treatment of attention-deficit/hyperactivity disorder secondary to traumatic brain injury: a critical appraisal of treatment studies | Only one database searched |
| Kebir et al, 2009 | Candidate genes and neuropsychological phenotypes in children with ADHD: review of association studies | Only one database searched |
| Miller et al, 1999 | Appropriateness of psychostimulant prescription to children: theoretical and empirical perspectives | Only one database searched |
| Snyder et al, 2006 | A meta-analysis of quantitative EEG power associated with attention-deficit hyperactivity disorder | Only one database searched |
| Cornell et al, 2018 | A systematic review of play-based interventions for students with ADHD: implications for school-based occupational therapists | Database search not including Medline or Pubmed |
| Lambez et al, 2020 | Non-pharmacological interventions for cognitive difficulties in ADHD: A systematic review and meta-analysis | Database search not including Medline or Pubmed |
| Nielsen et al, 2017 | Occupational Therapy Interventions for Children with Attention Deficit Hyperactivity Disorder: A Systematic Review | Database search not including Medline or Pubmed |
| Castells et al, 2021 | Relationship Between Treatment Duration and Efficacy of Pharmacological Treatment for ADHD: A Meta-Analysis and Meta-Regression of 87 Randomized Controlled Clinical Trials | Database search not including Medline or Pubmed |
| Gaastra et al, 2016 | The Effects of Classroom Interventions on Off-Task and Disruptive Classroom Behavior in Children with Symptoms of Attention-Deficit/Hyperactivity Disorder: A Meta-Analytic Review | Database search not including Medline or Pubmed |
| Fabiano et al, 2009 | A meta-analysis of behavioral treatments for attention-deficit/hyperactivity disorder | Database search not including Medline or Pubmed |
| Lundahl et al, 2006 | A meta-analysis of parent training: moderators and follow-up effects | Database search not including Medline or Pubmed |
| Salehinejad et al, 2019 | Correction: Transcranial direct current stimulation in attention-deficit hyperactivity disorder: A meta-analysis of neuropsychological deficits | Part of previous included article |
| Kofler et al, 2016 | Is hyperactivity ubiquitous in ADHD or dependent on environmental demands? Evidence from meta-analysis | Did not minimum inclusion criteria |
| Zhang et al, 2020 | Chronic Physical Activity for Attention Deficit Hyperactivity Disorder and/or Autism Spectrum Disorder in Children: A Meta-Analysis of Randomized Controlled Trials | Lacked sufficient information about ADHD |
| Aarnoudse-Moens et al, 2009 | Meta-analysis of neurobehavioral outcomes in very preterm and/or very low birth weight children | Lacked sufficient information about ADHD |
| Hammad et al, 2006 | Suicidality in pediatric patients treated with antidepressant drugs | Lacked sufficient information about ADHD |
| Thaler et al, 2009 | Bach Flower Remedies for psychological problems and pain: a systematic review | Lacked sufficient information about ADHD |
